# Isolation of a Square Pyramidal Bis(amidophenolate)‐Supported As(III)‐Cation: Coordination‐Induced Electromerism at As

**DOI:** 10.1002/anie.202501439

**Published:** 2025-04-04

**Authors:** Simon B. H. Karnbrock, Jan F. Köster, Christopher Golz, Ricardo A. Mata, Manuel Alcarazo

**Affiliations:** ^1^ Institut für Organische und Biomolekulare Chemie Georg‐August‐Universität Göttingen Tammannstraße 2 37077 Göttingen Germany; ^2^ Institut für Physikalische Chemie Georg‐August‐Universität Göttingen Tammannstraße 6 37077 Göttingen Germany

**Keywords:** Arsenic, Electromerism, Main group, Redox active ligand, Valence tautomerism

## Abstract

The synthesis and structural characterization of a slightly distorted square pyramidal arsenium cation is reported. Spectroscopic, crystallographic, and computational evidence indicate that coordination of typical Lewis bases to the As(III)‐center induces its oxidation to As(V) with concomitant reduction of the supporting, redox‐active bis(amidophenolate) ligand. That reaction is reversible and represents a well‐defined example of base‐induced electromerism in an As‐complex. Single electron reduction of the same As(III) species initially generates a ligand‐centered radical, which is persistent enough to be observed by EPR but slowly decays to a heterodimer. Preliminary experiments demonstrate the utility of the As(III)‐cation as initiator for transfer hydrogenation reactions.

## Graphical abstract

When a bis(amidophenolate)‐supported As(III)‐center is treated with typical two‐electron donors, a coordination‐induced electromerism induces the oxidation of the central atom to As(V) with concomitant reduction of the redox‐active ligand. Due to this triggerable electronic reorganization, the As‐center remains a remarkable Lewis acid and can be used as initiator for transfer hydrogenation reactions.

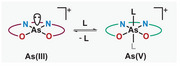

The facile and reversible interconversion between stable oxidation states of a metal is the fundamental cornerstone that enables the gearing of most synthetically useful catalytic cycles; however, and despite being tremendously successful, the protocols based on this approach often require the use of well‐behaved, low‐abundant, and noble metals.^[^ ^1^, ^2^, ^3^ ^]^ A strategy to overcome this limitation consists of the use of redox‐active ligands that are themselves also capable of participating in reduction and/or oxidation events.^[^ ^4^, ^5^, ^6^, ^7^, ^8^, ^9^ ^]^ Cooperation with such ligands confers nobility to base metals, leading to complexes/catalysts that are able to undergo well‐defined redox cycles even when the valence electrons of the central metal are inaccessible.^[^ ^10^ ^]^

A phenomenon intrinsically related to the assembly of transition metal complexes using redox‐active ligands is valence isomerism, also called electromerism, which is associated with the electronic redistribution between distinguishable redox‐active centers in a molecule (Scheme 1a).^[^ ^11^, ^12^, ^13^, ^14^, ^15^, ^16^ ^]^ Discrete electromers are only observed if a substantial barrier hinders their interconversion.^[^ ^17^ ^]^ This is usually the case when a geometrical rearrangement at the metal coordination sphere occurs and is often induced by the coordination or dissociation of a ligand;^[^ ^18^, ^19^, ^20^ ^]^ yet, it can also be triggered by other stimuli such as temperature,^[^ ^21^, ^22^, ^23^, ^24^, ^25^, ^26^ ^]^ light,^[^ ^27^, ^28^, ^29^, ^30^ ^]^ or pressure.^[^ ^31^, ^32^, ^33^ ^]^ The significantly different physicochemical properties that usually characterize electromers make them attractive compounds for the development of display devices, sensors, and data storage materials.^[^ ^11^, ^12^, ^13^, ^14^, ^15^, ^16^, ^34^, ^35^, ^36^, ^37^, ^38^ ^]^ Catalytic cycles capitalizing on electromers have also been engineered.^[^ ^39^, ^40^, ^41^ ^]^

**Scheme 1:**
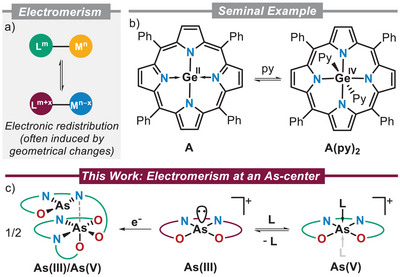
a) Concept of electromerism; b) Vaid's seminal work on p‐block coordination‐induced electromerism; c) Schematic overview of this work.

Examples of electromerism involving p‐block elements still remain scarce,^[^ ^42^, ^43^, ^44^, ^45^, ^46^, ^47^, ^48^, ^49^, ^50^, ^51^ ^]^ with the pyridine‐induced Ge(II) to Ge(IV) oxidation in porphyrin complex **A** being the seminal example (Scheme 1b).^[^ ^52^ ^]^ In our efforts to understand the effects of redox non‐innocent ligands in main group chemistry, and specifically, their implications for the design of pnictogen‐based catalysts,^[^ ^53^ ^]^ we became interested in the intricacies of electromerism. The following study describes the synthesis of the As(III) cation **5** supported by a tetradentate bis(amidophenolate) ligand, which undergoes well‐defined As(III)/(V) electromerism upon ligand coordination (Scheme 1c). Interestingly, single‐electron reduction of **5** delivers the mixed As(III)/(V) dimer **10**. Theoretical calculations suggest that electromerism also facilitates the dimerization process.^[^ ^54^ ^]^

Our research started with the treatment of ligand **1** with AsCl_3_ in the presence of triethylamine, which results in the isolation of arsine complex **2** as a white crystalline solid in multigram scale and excellent yield (Scheme 2a). Note that under otherwise identical reaction conditions, **1** reacts with PCl_3_ to afford the corresponding hydridophosphorane (10‐P‐5 species);^[^ ^55^, ^56^ ^]^ however, already from the nonsymmetric ^1^H NMR spectrum of **2**, and subsequently through X‐ray crystallography, it was confirmed that the final N–H oxidative addition step that would lead to the corresponding hydridoarsorane does not occur. Arsine **2** was converted into the arsoranide salt **3** by deprotonation with potassium hexamethyldisilazane, or alternatively, to chloroarsorane **4** through oxidation with *N*‐chlorosuccinimide. In the solid state, the anionic component of **3** exhibits a distorted disphenoidal structure with N1─As1─O2 and O1─As1─N2 angles of 151.0(1)° and 115.4(1)°, respectively, while in **4**, the tetradentate ligand imposes a pronounced square pyramidal geometry (*τ*_5_ = 0.08).^[^ ^57^ ^]^ Both structures compare well with the values obtained from gas‐phase calculations at the B3LYP‐D3(BJ)/def2‐TZVP level of theory^[^ ^58^, ^59^, ^60^, ^61^, ^62^, ^63^, ^64^ ^]^ and exclude crystal packing as the primary reason for the distortion in **3**.

**Scheme 2:**
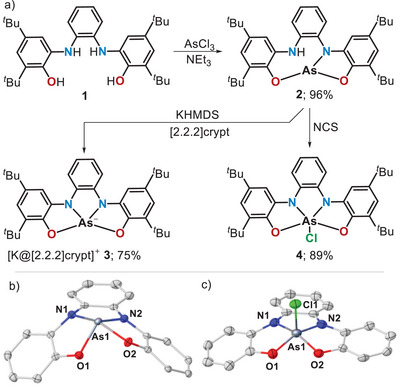
a) Synthesis of potassium arsoranide **3** and chloroarsorane **4**; b) and c) Molecular structures of **3** and **4** in the solid state. Cation, solvent molecules, *tert*‐butyl groups, and protons removed for clarity. Thermal ellipsoids are shown at 50% probability. Selected metrical parameters: **3**; O1─As1 = 1.886(1) Å, O2─As1 = 1.944(1) Å, N1─As1 = 2.006(1) Å, N2─As1 = 1.913(1) Å, O1─As1─N2 = 151.0(1)°, O2─As1─N1 = 115.4(1)°; **4**; O1─As1 = 1.782(6) Å, O2─As1 = 1.782(6) Å, N1─As1 = 1.824(7) Å, N2─As1 = 1.812(8) Å, O1─As1─N2 = 153.5(3)°, O2─As1─N1 = 158.3(3)°.^[^ ^65^ ^]^

Next, the chloride abstraction from **4** was pursued by reaction with TMSOTf. Interestingly, the mixture immediately turned dark green, which is a first hint pointing to the oxidation of the supporting ligand to its diimine form.^[^ ^66^, ^67^ ^]^ In fact, the UV/vis spectrum of a solution of the newly formed **5** exhibits a broad band at 898 nm (*ε* = 15 100 M^−1^ cm^−1^) responsible for its color, and further characteristic transitions at 683 nm (*ε* = 5400 M^−1^ cm^−1^) and 448 nm (*ε* = 4700 M^−1^ cm^−1^), while that of **4** is dominated by high‐energy transitions with maxima at 253 nm and 297 nm (Scheme 3a). Compound **5** was subsequently isolated as a crystalline solid, and its connectivity was established by X‐ray crystallography. In **5**, the As‐center adopts a square pyramidal geometry with *trans*‐basal angles O1─As1─N2 and N1─As1─O2 of 137.1(1)° and 127.0(1)°, respectively, and a sum of basal angles O1─As1─N1, N1─As1─N2, O2─As1─N1, and O1─As1─O2 of 322.8° (Figure 1a). Moreover, the As1─O1, As1─O2, As1─N1, and As1─N2 bonds (1.858(1), 1.857(1), 1.980(2), and 2.044(2) Å, respectively) are substantially elongated in comparison to those in **4**; this indicates the reduction of the As(V)‐center to As(III) upon chloride dissociation. The analysis of the bond length at the bis(amidophenolate) ligand in **5** supports this conclusion as well. The N1─C15 and N2─C16 bond distances (1.340(2) and 1.332(2) Å, respectively) denote a partial double bond between these atoms, while the pronounced alternation of the C─C bond lengths in the central phenylene is indicative of the partial dearomatization of that moiety (Figure 1a).^[^ ^68^ ^]^ Finally, electronic structure calculations at the B3LYP‐D3(BJ)/def2‐TZVP level of theory and natural bond orbital (NBO) analysis support this case of electromerism induced by chloride abstraction. The NBO charge at arsenic is calculated to be +2.25 for the pentacoordinated As(V) species **4**, but unintuitively, it is reduced to only +1.66 in tetracoordinated **5** despite the net positive charge that is generated in the complex by removal of the chloride anion (Figure 2).^[^ ^69^ ^]^

**Scheme 3:**
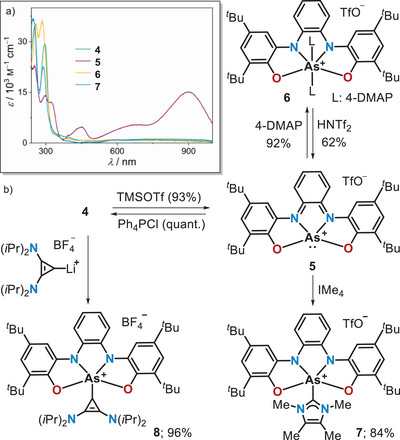
a) UV–vis spectra of **4**–**7**; b) Synthesis and reactivity of arsenium cation **5** toward typical two‐electron donors.

**Figure 1:**
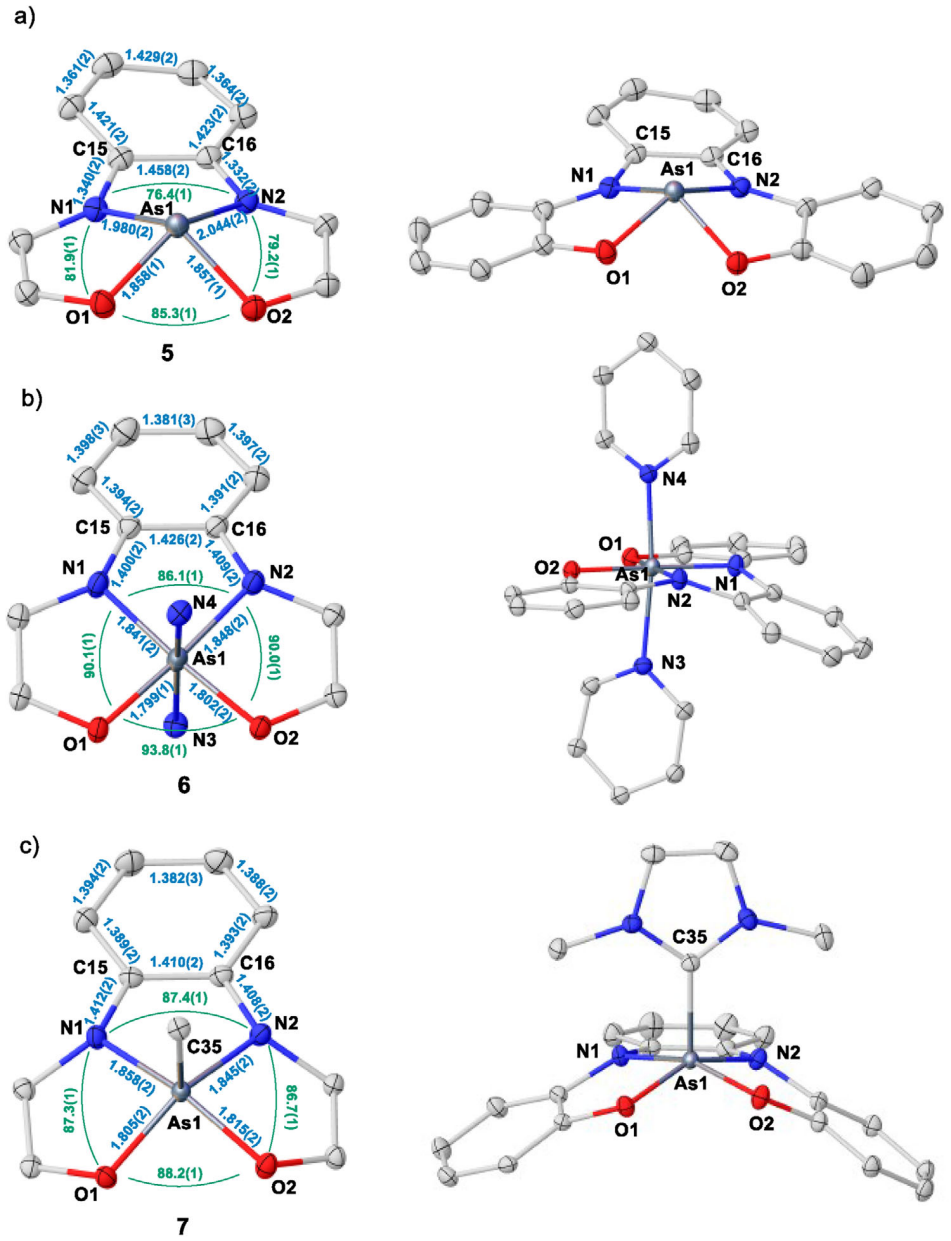
a)–c) Solid‐state structures of **5**, **6**, and **7**, respectively. Anions, solvent molecules, *tert*‐butyl, Me_2_N‐, NHC‐methyl, and protons were removed for clarity. Thermal ellipsoids are shown at 50% probability. Selected bond length (blue) and angles (green).^[^ ^65^ ^]^

**Figure 2:**
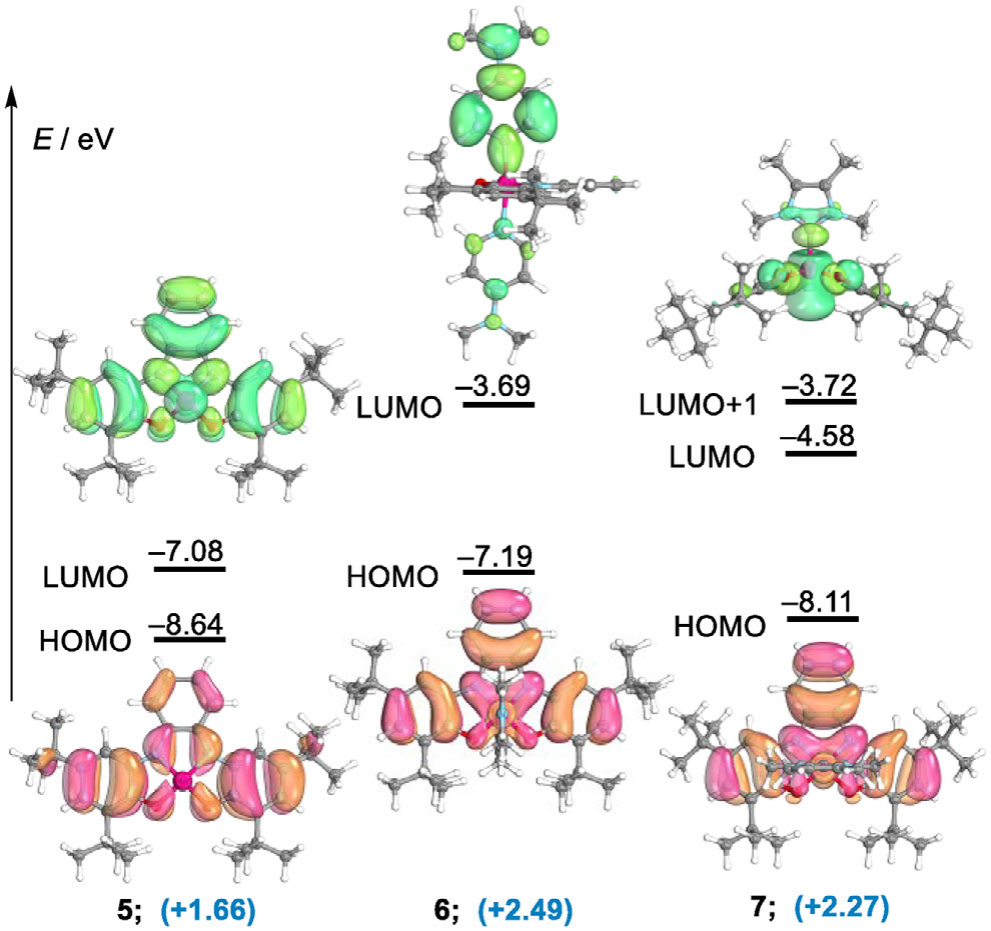
Frontier orbital energies and Kohn–Sham HOMOs and LUMOs of **5**, **6**, and **7** at the B3LYP‐D3(BJ)/def2‐TZVP level. NBO charges at the As‐center are depicted in blue.

With **5** available in substantial amounts, we set out to investigate whether archetypical neutral ligands trigger the shuttle of electrons between the As‐center and the bis(amidophenolate) scaffold as well. Initially, we checked the reaction of **5** with 4‐dimethylaminopyridine (4‐DMAP), obtaining a discrete product only when two equivalents of 4‐DMAP were employed. In that case, compound **6** was isolated as a light‐yellow solid (Scheme 3b; see Scheme S1 for a thermochemical discussion). Subsequently, crystals of **6** were grown from *o‐*DCB/pentane and analyzed by X‐ray diffraction, confirming the coordination of two 4‐DMAP ligands in apical positions (Figure 1b). In **6**, the As‐center is exactly located in the plane defined by the bis(amidophenolate) ligand (sum of equatorial angles: 360.0°) and exhibits a pseudo‐octahedral coordination geometry with an almost straight angle between the two axial pyridines (N3─As1─N5 = 172.1(1)°). The bond metrics of the bis(amidophenolate) ligand confirm its completely reduced nature, and the As1─O1, As1─O2, As1─N1, and As1─N2 bonds (1.799(1), 1.802(1), 1.841(1), and 1.848(1) Å, respectively) get shortened in comparison to those in **5** despite the increased coordination number at As from four to six. In addition, our computational analysis shows that the NBO charge at As in **6** is +2.49 (Figure 2). These cumulative evidences verify the As(III) → As(V) electromerism as a result of pyridine coordination. The reaction is reversible, and the addition of HNTf_2_ to **6** releases **5** by protonation of the 4‐DMAP ligands.

Additional proof of the proposed electromerism was obtained by analysis of the geometrical parameters of compounds **7** and **8**, both carbene adducts of **5** (Figures 1c, S16, and S18). Note that in these species only one carbene ligand coordinates the As‐center, and as a consequence, they adopt a square pyramidal geometry (as chloroarsorane **4**). This is due to the strong donor character of the carbenes that push the σ*(As‐C) orbital (LUMO + 1 in **7** and **8**) to relatively high levels of energy, reducing the appetence of As to coordinate a second carbene (see Figure S23 to compare the LUMO + 1 in **7** and **8** with those of the non‐isolated **5^.^py** and **5^.^DMAP** adducts). All structural parameters in **7** and **8** certificate again the reduction of the bis(amidophenolate) unit and the concomitant oxidation of the central atom to As(V) (Figures 1c and S18). It is also significant that the LUMO of **5** and the HOMOs of **6**–**8** are basically identical in shape, confirming the transfer of electron density from As to the bis(imidophenolate) upon coordination of the external ligand(s) (Figures 2 and S23).

Having isolated arsoranide **3** and arsenium cation **5**, we set out to complete the redox series connecting both structures. Cyclic voltammetry of a DCM solution of **3** showed two quasi‐reversible oxidation waves at *E*_ox,1_ = –0.48 V and *E*_ox,2_ = –0.02 V versus Fc^+/0^, which are indicative of limited stability of the neutral open‐shell species (Figure 3b). The solutions obtained by mixing equimolar amounts of Ag[CB_11_H_6_Cl_6_] and **3** in *o‐*DCB are EPR active, yet the intensity of the spectrum rapidly decays after several minutes at r.t. An identical observation was made when making react **3** and **5** under otherwise identical conditions. The recorded X‐band isotropic spectrum exhibits a characteristic quartet of quintet splitting that has been simulated with hyperfine constants of 147.6 and 6.1 G to one ^75^As(I = 3/2) and two ^14^N(I = 1) nuclei, respectively, and a *g*‐value of 2.0085 (Figure 3c). DFT calculations at the UB3LYP‐D3(BJ)/def2‐TZVP level of theory for radical **9** reproduce these features (isotropic Fermi contact couplings of 158.1 G to the central As nucleus and 5.1 G to the ligand‐based ^14^N nuclei) and provide a quantitative picture of the spin distribution in that radical. The SOMO of **9** is mainly delocalized on the bis(amidophenolate) ligand, and the Mulliken spin density at As is only 4.8%; the As NBO charge is estimated to be +1.60, nearly identical to that of **5**. This cumulative experimental and calculated evidence is consistent with the formulation of **9** as an As(III) center surrounded by an open‐shell mono‐oxidized ligand (Figure 3a–c). Note that a similar analysis for the phosphorus analogous of **9** concluded that such species possess a remarkable P(IV) character (spin density at *P* = 45.8%).^[^ ^70^ ^]^

**Figure 3:**
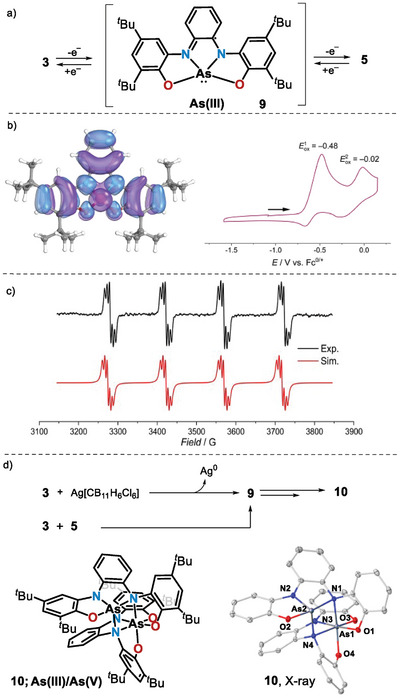
a) Redox series from arsoranide **3** to arsenium **5** via transient radical **9**, b) SOMO of **9** at the UB3LYP‐D3(BJ)/def2‐TZVP level and CV of **3**; c) X‐band EPR spectrum of **9**, d) Generation and X‐ray solid‐state structure of heterodimer **10**. Hydrogen atoms, solvent molecules, and *tert*‐butyl groups were omitted for clarity. Ellipsoids are set at 50% probability. Selected metrical parameters: O1─As1 = 1.826(1) Å, N1─As1 = 1.966(2) Å, N3─As1 = 1.860(2) Å, N4─As1 = 2.037(2) Å; O3─As1 = 1.791(1) Å; O4─As1 = 1.853(1) Å, O2─As2 = 1.823(1) Å; N1─As2 = 2.356(2) Å, N2─As2 = 1.854(2) Å, N4─As2 = 2.064(2) Å.^[^ ^65^ ^]^

In an attempt to isolate **9**, compounds **3** and **5** were allowed to react for 30 min. at r.t. The yellow precipitate formed was filtered, washed, and dried; yet, the isolated solid was not radical **9** but its heterodimer **10** (68%), whose connectivity was unambiguously established by X‐ray diffraction (Figure 3d). In **10**, a disphenoidal As(III) and an octahedral As(V) center are bridged through two *μ*‐amido donors; moreover, the bond metrics indicate a fully reduced tetraanionic state of both bis(amidophenolate) ligands. Clean and well‐defined ^1^H and ^13^C NMR spectra of **10** are recorded at −40 °C, but upon warming an additional set of signals appear, and the sample becomes EPR‐active, showing the characteristic spectrum of **9**, albeit with very weak intensity. This process is reversible; the relative ratio of **10** increases again when the sample is cooled down, and clean **10** is recovered as a solid by evaporation of the solvent. In addition, diffusion‐ordered NMR (DOSY) experiments indicated that the additional diamagnetic species detected by NMR is also a dimer of **9** (Figure S4).^[^ ^71^, ^72^ ^]^ Willing to find an explanation for these observations, the dimerization process leading to **10** was evaluated through computational methods. The reaction intermediates and transition states were optimized at the PBE‐D3(BJ)/def2‐SVP level of theory,^[^ ^73^ ^]^ making use of the D3 Grimme dispersion correction with Becke–Johnson damping.^[^ ^61^ ^]^ For computational efficiency, truncated geometries were used in which the *tert*‐butyl moieties were substituted by methyl groups (Figure 4).

**Figure 4:**
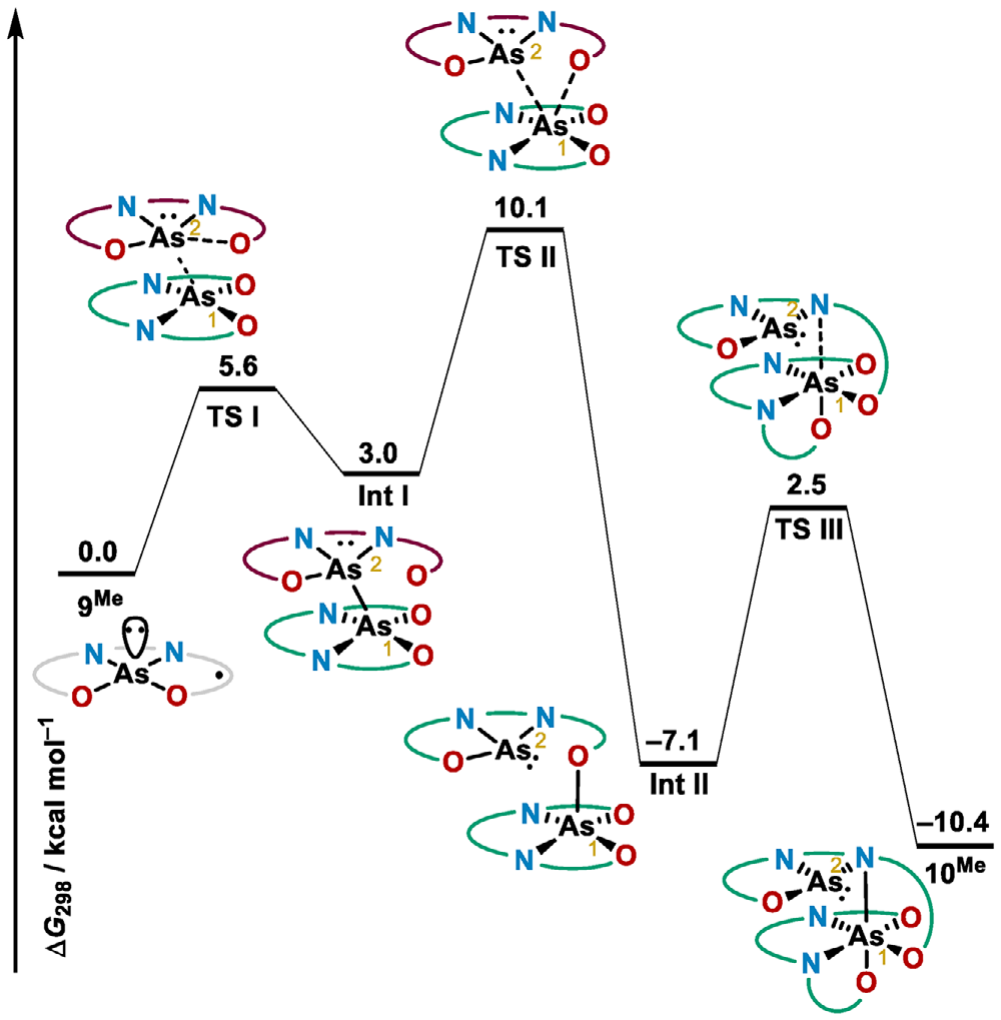
Free energy profile for the formation of dimer **10** calculated at the (U)B3LYP‐D3(BJ)/def2‐TZVP(CPCM)//(U)PBE‐D3(BJ)/def2‐SVP level of theory.

Three main steps have been identified for the transformation of **9** into **10**. Initially, the As‐As bond is formed with concomitant dissociation of one of the phenolate arms from As(2) to deliver **IntI** through **TSI**. In **IntI** the As(2) atom exhibits a distorted disphenoidal structure, and the bis(amidophenolate) ligand originally surrounding As(2) gets oxidized. In a second step, the free phenolate arm coordinates the neighboring As(1); this occurs with simultaneous cleavage of the As─As bond forming **IntII**. Note that in **IntII** both bis(amidophenolate) ligands are at their reduced state again, and the disproportionation between the As‐centers has already occurred. Finally, migration of a phenolate ligand in As(1) to the apical position facilitates the incorporation of an additional nitrogen atom to the coordination sphere of As(1), delivering **10**. This mechanistic picture suggests that the additional dimeric species detected by NMR at r.t. is probably **IntII,** albeit the reversible isomerization of **10** to a diastereomer cannot be ruled out.

As result of the remarkable hydride ion affinity calculated for **5** (838 kJ mol^−1^; see the Supporting Information), it was hypothesized that this compound might serve as an efficient hydride abstractor, and consequently, it could be used as an initiator for transfer hydrogenation reactions. The dihydrogen transfer between 1,4‐cyclohexadiene **11** and 1,1‐diphenylethylene **12** was chosen as model to evaluate this possibility (Scheme 4).^[^ ^74^ ^]^ To our delight, alkane **14** was formed in nearly quantitative yield after 8 h at 60 °C, whereas compound **7**, in which the Lewis acidity is quenched by the coordinated NHC ligand, is not able to promote the reaction.

**Scheme 4:**
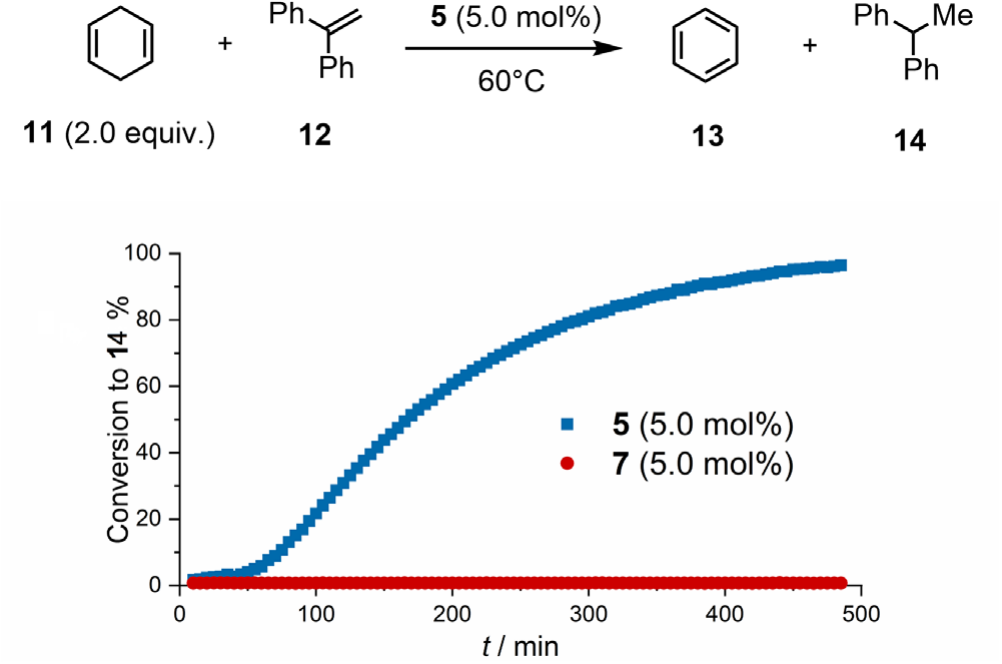
Transfer hydrogenation to 1,1‐diphenyl ethane using cyclohexa‐1,4‐diene as the dihydrogen source. The reaction progress is monitored by quantitative ^1^H NMR spectroscopy. [Correction added on 09 April 2025 after first online publication: Scheme 4 has been updated.]

In summary, we describe herein the synthesis and characterization of a square pyramidal arsenium cation **5** supported by a redox‐active bis(amidophenolate) ligand. Upon addition of a Lewis base, the cation undergoes a well‐defined As(III)/(V) electromerism, as supported by X‐ray crystallography, UV–vis spectroscopy, and electronic structure calculations. The process is reversible and can be reverted by acid treatment. In addition, the single‐electron reduction of **5** generates a mixed As(III)/(V) dimer **10**, which is in thermal equilibrium with a second diamagnetic species, probably **IntII**. The triggerable electronic reorganization observed in **5** might find applications in p‐block element redox‐catalysis; actually, we found this compound to be an appropriate initiator for transfer hydrogenation reactions.

## Conflict of Interests

The authors declare no conflict of interest.

## Supporting information



Supporting Information

Supporting Information

## Data Availability

The data that support the findings of this study are available in the supplementary material of this article.
