# Supplementary material for: Isolation of a Square Pyramidal Bis(amidophenolate)‐Supported As(III)‐Cation: Coordination‐Induced Electromerism at As
Source: Angew Chem Int Ed Engl. 2025 Apr 4;64(23):e202501439. doi: 10.1002/anie.202501439 (PMC12124349; doi:10.1002/anie.202501439)
Supplement: Supplementary file 1 — Supporting Information [file ANIE-64-e202501439-s002.pdf]

Supporting Information

"Isolation of a Square Pyramidal Bis(amidophenolate)-supported As(III)-Cation: Coordination-Induced Electromerism at As"

Contents

General Information 4

Experimental Procedures 6

- Synthesis of Arsine 2 6
- Synthesis of Potassium Arsoranide 3 6
- Synthesis of Chloroarsorane 4 7
- Synthesis of Arsenium triflate 5 7
- Synthesis of 4-DMAP-adduct 6 8
- Synthesis of NHC-adduct 7 8
- Synthesis of BAC-adduct 8 9
- Generation of radical 9 10
- Synthesis of As(III)/(V) heterodimer 10 10
- Identification of 10 from mixtures of radical 9 11
- Detection of 9 in solutions of 10 12
- Variable Temperature NMR of 10 13
- DOSY NMR of 10 14
- Attempted Trapping of 9 15
- Reaction of 5 with Ph_4PCl 16
- Reaction of 6 with $HNTf_2$ 16
- Transfer Hydrogenation 17

Crystallographic supplement 21

- General Information 21
- Refinement details for 2 22
- Refinement details for 3 23
- Refinement details for 4 24
- Refinement details for 5 25
- Refinement details for 6 26
- Refinement details for 7 27
- Refinement details for 8 28
- Refinement details for 10 (two solvates) 29
- Metrical Analysis of Compounds 4-8 31

Computational Details 32

- General Information 32
- Thermochemistry for Formation of 6 from 5 and 4-DMAP 32
- Selected Molecular Orbitals and Natural Bond Orbitals of 5 33
- Frontier Molecular Orbital Diagram of Lewis Base adducts of 5 33

Natural Resonance Theory 34
Hydride and Fluoride Ion Affinities 34
Energetic Landscape for the Dimerization of Radical 9 35
Cartesian Coordinates of Optimized Geometries 36
NMR Spectra 71
References 82

General Information

Working methods:

Unless otherwise stated, all manipulations were carried out under a nitrogen atmosphere in flame-dried glassware on a Schlenk line or under nitrogen atmosphere in an MBraun UNIlab plus glovebox. Dry solvents were obtained using an MBraun MB-SPS-7 solvent purification system (THF, diethyl ether, toluene, pentane, dichloromethane, acetonitrile), deoxygenated in a nitrogen stream and stored over 3 Å molecular sieves under a nitrogen atmosphere. Benzene was distilled over Na/benzophenone and stored over a Na-mirror. 1,2-Dichlorobenzene (*o*DCB), $CHCl_3$ and fluorobenzene were deoxygenated in a nitrogen stream and dried over 3 Å molecular sieves under a nitrogen atmosphere.

Starting materials:

Unless otherwise specified, all reagents were used as received from commercial suppliers (ABCR, AcrosOrganics, Alfa Aesar, Chempur GmbH, J and K Scientific, Sigma Aldrich, Thermo Fisher Scientific, Tokyo Chemical Industry). Triethylamine was dried over 3 Å molecular sieves and deoxygenated in a nitrogen stream. 4-Dimethylaminopyridine (4-DMAP) was purified under nitrogen atmosphere by sublimation at 80 °C and 10^{-3} mbar and stored in a nitrogen-filled glovebox. *N,N'*-bis(3,5-di-*tert*-butyl-2-hydroxyphenyl)-1,2-phenylenediamine [1] (**1**), 1,3,4,5-tetramethyl-1,3-imidazol-2-ylidene[2] (IMe_4) and Weiss-Yoshida reagent bis(diisopropylamino)cyclopropenylidene · 1.25 $LiBF_4$[3] (**S1**) were synthesized according to the published procedures.

NMR spectroscopy:

The NMR spectra were recorded on a Bruker Avance III 300 MHz, Bruker Avance III HD 300 MHz, Bruker Avance III 400, Bruker Avance III HD 400 MHz or Bruker Avance Neo 400 MHz at 298 K. ^{1}H and ^{13}C chemical shifts are given in ppm relative to TMS, using the solvent signals as references and converting the chemical shifts to the TMS scale. ^{11}B and ^{19}F chemical shifts are given in ppm relative to $BF_3{\cdot}OEt_2$ and $CFCl_3$, respectively (external standard). The chemical shifts are given in parts per million (ppm), and the coupling constants (*J*) in Hertz (Hz). Solvents for NMR spectroscopy were deoxygenated in a nitrogen stream or alternatively by three freeze-pump-thaw cycles and stored over 3 Å molecular sieves in a glove box.

Mass spectrometry:

Mass spectrometry analyses were performed using the following equipment: Bruker Daltronik microTOF (ESI), Bruker Daltronik maXis (ESI), Joel AccuTOF (LIFDI).

Infrared spectroscopy:

Neat samples were measured on a JASCO FT/IR-4100 or JASCO FT/IR-4600 at room temperature. The vibrational frequencies are reported in wavenumbers (cm^{-1}).

UV/vis spectroscopy:

UV/vis spectroscopy was performed at room temperature in a nitrogen-filled glovebox with dry and degassed solvents. The Avantes AvaLight-DHc Full-range Compact Light Source was used in combination with an Avantes AvaSpec-UL S2048 UV/Vis Fiber-Optic Spectrometer. Spectra were measured using the Pine Research AfterMath Software package.

EPR spectroscopy:

EPR-spectra were measured on a Bruker EMX mikro X-Band EPR from BRUKER Biospin with the Bruker Xenon Software. The spectra were measured in dry and degassed solvents at room temperature unless otherwise stated.

Cyclic voltammetry:

Cyclic voltammetry was performed with a VersaSTAT 4 potentiostat from Princeton Applied Research using the VersaStudio software (version 2.44.4). A standard three electrodes setup was used with a glassy carbon working electrode, a platinum counter electrode and a silver/silver chloride pseudo-reference electrode. Internal referencing was performed against $[Co(Cp)_2]^{0/+}$.

Elemental analyses:

Elemental analyses were obtained from the Analytisches Labor, Georg-August-Universität, Göttingen, using an Elementar Vario EL 3 analyzer.

Experimental Procedures

Synthesis of Arsine **2**

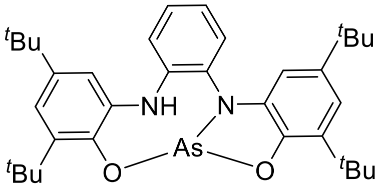

A Schlenk flask equipped with a magnetic stirring bar was charged with ligand **1** (5.00 g, 9.68 mmol, 1.00 equiv.). Et_2O (50 mL) and triethylamine (4.05 mL, 29.0 mmol, 3.00 equiv.) were added subsequently and the solution was cooled to –78 °C. $AsCl_3$ (0.81 mL, 9.7 mmol, 1.0 equiv.) was slowly added *via* syringe and the reaction mixture was allowed to warm up to ambient temperature over the course of 16 h. The formed suspension was concentrated to half of its volume, filtered and the residue was extracted with Et_2O (2 × 10 mL). Drying of the filtrate *in vacuo* gave the title compound as a colorless solid (5.47 g, 9.29 mmol, 96%).

^{1}H NMR (400 MHz, CD_2Cl_2): δ (ppm) = 7.58 (t, *J* = 1.9 Hz, 1H), 7.56 (t, *J* = 1.7 Hz, 1H), 7.30 – 7.23 (m, 2H), 7.22 (d, *J* = 2.4 Hz, 1H), 7.15 (d, *J* = 2.4 Hz, 1H), 6.97 (td, *J* = 7.7, 1.3 Hz, 1H), 6.89 (d, *J* = 2.1 Hz, 1H), 5.04 (s, 1H), 1.53 (s, 9H), 1.27 (s, 9H), 1.27 (s, 9H), 1.13 (s, 9H).

^{13}C{^{1}H} NMR (101 MHz, CD_2Cl_2): δ (ppm) = 153.7, 148.4, 142.7, 142.2, 140.3, 139.6, 136.2, 135.3, 132.5, 129.7, 128.0, 125.1, 123.9, 121.2, 121.2, 117.3, 115.6, 106.3, 35.67, 35.2, 35.0, 34.7, 32.0, 31.7, 30.0, 29.5.

IR (ATR, neat) [cm−1] $\tilde{\nu}$ = 3317, 2952, 2904, 2866, 1599, 1574, 1491, 1442, 1421, 1358, 1339, 1308, 1276, 1231, 1201, 1166, 1118, 997, 904, 857, 825, 739, 712, 674, 653, 624, 584, 541, 512, 491, 463.

HR-MS-ESI(+) *m*/*z* calcd. for $C_{34}H_{45}N_2O_2As^+$ [M]$^+$ 588.2692, found 588.2697.

Anal. calcd. for $C_{34}H_{45}N_2O_2As$: C 69.37, H 7.71, N 4.76, found: C 69.07, H 7.65, N 4.76.

m. p. 235 °C (decomp.).

Synthesis of Potassium Arsoranide **3**

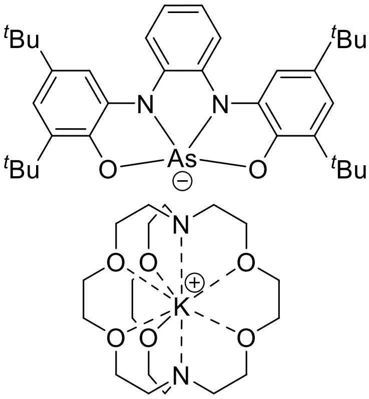

In a Schlenk flask containing a magnetic stirring bar, arsine **2** (200 mg, 315 µmol, 1.00 eq.) and [2.2.2]-cryptand (128 mg, 315 µmol, 1.00 eq.) were dissolved in Et_2O (5 mL). KHMDS (62.8 mg, 315 µmol, 1.00 eq.) in Et_2O (1.5 mL) was added dropwise *via* syringe under rigorous stirring resulting in precipitation of a white solid. The mixture was stirred for 20 min at room temperature. Afterwards the mixture was filtered and washed with Et_2O (2 x 3 mL). The residue was dried *in vacuo* to yield the target compound as a colorless solid (295.3 mg, 281.2 µmol, 89%).

^{1}H NMR (300 MHz, CD_3CN): δ (ppm) = 7.44 – 7.53 (m, 2H), 7.31 (d, *J* = 2.3 Hz, 2H), 6.79 – 6.69 (m, 4H), 3.51 (s, 12H), 3.46 (t, *J* = 4.6 Hz, 12H), 2.46 (t, *J* = 4.6 Hz, 12H), 1.39 (s, 18H), 1.31 (s, 18H).

^{13}C{^{1}H} NMR (101 MHz, CD_3CN): δ (ppm) = 149.3, 140.6, 138.7, 138.0, 132.9, 119.4, 115.0, 113.9, 111.4, 71.2, 68.4, 54.7, 35.5, 35.0, 32.3, 30.0.

IR (ATR, neat) [cm^{-1}] $\tilde{\nu}$ = 2949, 2884, 2816, 1557, 1478, 1421, 1354, 1281, 1257, 1220, 1132, 1100, 1080, 991, 949, 931, 856, 832, 733, 690, 650, 593, 542, 520, 498, 458.

HR-MS-ESI(–) *m/z* calcd. for $C_{34}H_{44}N_2O_2As$ [M–K-crypt]$^-$ 587.2624 found 587.2621.

Anal. calcd. for $C_{52}H_{80}AsKN_4O_8$: C 62.25, H 8.04, N 5.58, found: C 61.96, H 8.01, N 5.48.

m. p. 223 °C (decomp.).

Synthesis of Chloroarsorane **4**

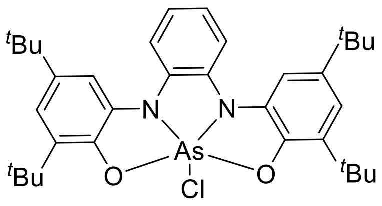

A Schlenk flask equipped with a magnetic stirring bar was charged with arsine **2** (1.00 g, 1.70 mmol, 1.00 equiv.) and *N*-chlorosuccinimide (229 mg, 1.72 mmol, 1.01 equiv.). DCM (5 mL) was added and the deep yellow-orange reaction mixture was stirred at ambient temperature for 2.5 h. The solvent was evaporated and the residue was extracted with hot pentane (6 × 5 mL) until the residue was colorless. The filtrate was evaporated and the formed solid was washed with cold (–40 °C) pentane (5 mL). Drying of the residue in high vacuum provided the title compound as a yellow solid (940 mg, 1.51 mmol, 89%).

^{1}H NMR (300 MHz, $CDCl_3$): δ 7.92 – 7.80 (m, 2H), 7.71 (d, *J* = 2.1 Hz, 2H), 7.25 – 7.15 (m, 2H), 7.12 (d, *J* = 2.1 Hz, 2H), 1.54 (s, 18H), 1.44 (s, 18H).

^{13}C{^{1}H} NMR (75 MHz, $CDCl_3$): δ 143.7, 140.3, 134.8, 128.6, 127.4, 121.2, 116.7, 111.5, 107.9, 35.2, 35.2, 31.9, 29.7.

IR (ATR, neat) [cm^{-1}]: $\tilde{\nu}$ = 2954, 2906, 2869, 1582, 1480, 1424, 1357, 1269, 1213, 1123, 995, 926, 893, 818, 730, 629, 554.

HR-MS-ESI(+) *m/z* calcd. for $C_{34}H_{44}AsN_2O_2^+$ [M-Cl]$^+$ 587.2613, found 587.2603.

Anal. calcd. for $C_{34}H_{44}ClN_2O_2As$: C 65.54, H 7.12, N 4.50, found: C 66.03, H 7.46, N 4.26.

m.p. 149 °C (decomp.).

Synthesis of Arsenium triflate **5**

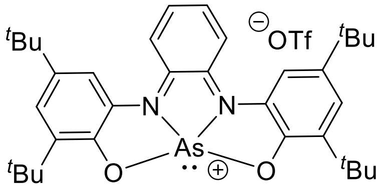

In a Schlenk flask equipped with a magnetic stirring bar, chloroarsorane **4** (500 mg, 802 µmol, 1.00 equiv.) was dissolved in DCM (3 mL). To this mixture TMSOTf (196 mg, 883 µmol, 1.10 equiv.) in DCM (2 mL) was added leading to an immediate color change to dark green. After stirring for 30 min, all volatiles were removed under reduced pressure and the residue was washed with pentane (2 × 3 mL). Drying of the remaining solid in high vacuum gave the title compound as a dark green solid (549 mg, 746 µmol, 93%).

^{1}H NMR (400 MHz, $CDCl_3$): δ 8.34 – 8.25 (m, 2H), 7.87 (d, *J* = 1.9 Hz, 2H), 7.86 – 7.81 (m, 2H), 7.61 (d, *J* = 1.9 Hz, 2H), 1.55 (s, 18H), 1.43 (s, 18H).

^{13}C{^{1}H} NMR (101 MHz, $CDCl_3$): δ 158.0, 149.2, 141.8, 140.0, 135.8, 131.6, 130.3, 120.6, 120.6 (q, *J* = 321.7 Hz), 111.9, 35.8, 35.6, 31.2, 29.5.

^{19}F NMR (377 MHz, $CDCl_3$): δ –78.1.

IR (ATR, neat) [cm^{-1}]: $\tilde{\nu}$ = 2955, 2906, 2869, 1505, 1349, 1262, 1138, 1030, 991, 913, 755, 634, 607, 544, 515.

HR-MS-ESI(+) *m/z* calcd. for $C_{34}H_{44}AsN_2O_2^+$ [M-OTf]$^+$ 587.2613, found 587.2607.

Anal. calcd. for $C_{35}H_{44}N_2O_5F_3SAs$: C 57.06, H 6.02, N 3.80, found: C 57.04, H 6.10, N 3.71.

m.p. 192 °C (decomp.).

Synthesis of 4-DMAP-adduct **6**

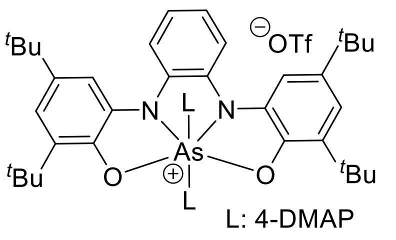

In a nitrogen filled glovebox, arsenium triflate **5** (50.0 mg, 67.9 µmol, 1.00 equiv.) was dissolved in $CHCl_3$ (0.5 mL). A solution of 4-DMAP (16.6 mg, 136 µmol, 2.00 equiv.) in $CHCl_3$ was added slowly resulting in a color change from dark green to yellow. The solution was layered with pentane and left for crystallization. Collection of the yellow crystals by filtration and drying of the material *in vacuo* gave the title compound as a yellow solid (61.1 mg, 62.3 µmol, 92%).

^{1}H NMR (400 MHz, $CDCl_3$): δ 7.81 (d, *J* = 7.0 Hz, 4H), 7.68 – 7.58 (m, 2H), 7.47 (d, *J* = 2.1 Hz, 2H), 7.15 – 7.07 (m, 2H), 6.89 (d, *J* = 2.1 Hz, 2H), 6.35 (d, *J* = 7.0 Hz, 4H), 3.03 (s, 12H), 1.44 (s, 18H), 1.33 (s, 18H).

^{13}C{^{1}H} NMR (101 MHz, $CDCl_3$): δ 155.5, 143.4, 141.4, 140.1, 134.1, 130.3, 127.7, 120.6, 120.4 (q, *J* = 320.5 Hz), 115.5, 110.2, 106.5, 105.9, 40.0, 34.9, 34.9, 31.6, 29.9.

^{19}F NMR (377 MHz, $CDCl_3$): δ –78.9.

IR (ATR, neat) [cm^{-1}]: $\tilde{\nu}$ = 2956, 2907, 2868, 1626, 1556, 1480, 1425, 1357, 1262, 1151, 1029, 810, 723, 634, 516.

HR-MS-ESI(+) *m/z* calcd. for $C_{41}H_{54}AsN_4O_2^+$ [M-DMAP-OTf]$^+$ 709.3457, found 709.3456.

Anal. calcd. for $C_{49}H_{64}N_6O_5F_3SAs$: C 59.99, H 6.58, N 8.57, found: C 59.80, H 6.55, N 8.46.

m.p. 132 °C (decomp.).

Synthesis of NHC-adduct **7**

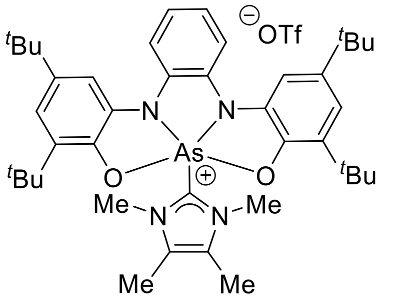

In a nitrogen-filled glovebox, a 4 mL-vial was charged with arsenium triflate **5** (100 mg, 136 µmol, 1.00 equiv.) and fluorobenzene (1 mL) was added. While stirring, a solution of IMe_4 (16.9 mg, 136 µmol, 1.00 equiv.) in fluorobenzene (1 mL) was added dropwise over 5 min resulting in a color change from dark green to dark yellow/brown. The solvent was removed under reduced pressure and the residue crystallized from a benzene/DCM mixture by slow evaporation. Afterwards the supernatant was removed and the residue washed with benzene (2 × 1 mL). Co-crystallized benzene was removed by azeotropic distillation with chloroform and subsequent drying in high vacuum provided the title compound as a yellow solid (98.3 mg, 114 µmol, 84%).

^{1}H NMR (300 MHz, CD_3CN): δ 8.12 – 8.02 (m, 2H), 7.83 (d, *J* = 2.1 Hz, 2H), 7.35 – 7.26 (m, 2H), 7.17 (d, *J* = 2.1 Hz, 2H), 3.75 (s, 6H), 2.13 (s, 6H), 1.50 (s, 18H), 1.39 (s, 18H).

^{13}C{^{1}H} NMR (126 MHz, CD_3CN): δ 144.3, 141.2, 138.9, 135.2, 132.4, 132.3, 131.5, 123.5, 122.2 (d, *J* = 320.9 Hz), 118.9, 113.8, 112.0, 35.7, 35.6, 35.2, 31.9, 30.2, 9.20.

^{19}F NMR (377 MHz, CD_3CN): δ –79.3.

IR (ATR, neat) [cm^{-1}]: $\tilde{\nu}$ = 2957, 2906, 2870, 1581, 1480, 1419, 1266, 1152, 1030, 993, 818, 732, 681, 637, 553, 518, 500, 406.

HR-MS-ESI(+) *m/z* calcd. for $C_{41}H_{56}AsN_4O_2^+$ [M-OTf]$^+$ 711.3614, found 711.3615.

Anal. calcd. for $C_{42}H_{56}AsF_3N_4O_5S$: C 58.60, H 6.56, N 6.51 found: C 58.57, H 6.69, N 6.17.

m.p. 136 °C (decomp.).

Synthesis of BAC-adduct **8**

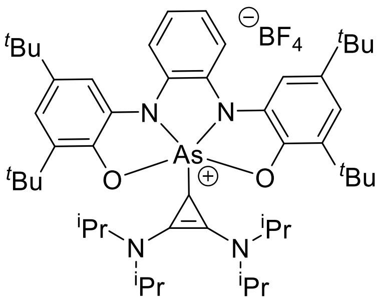

In a nitrogen-filled glovebox, a 4 mL-vial was charged with chloroarsorane **4** (100 mg, 160 µmol, 1.00 equiv.) and benzene (1 mL) was added. A solution of Weiss-Yoshida reagent **S1** (56.7 mg, 160 µmol, 1.00 equiv.) in benzene (1 mL) was added. After stirring for 1 min, the suspension was filtered and the filtrate immediately freeze-dried. The resulting solid was washed with pentane (3 × 2 mL) and dried in high vacuum. The title compound was obtained as a light-yellow solid (91.2 mg, 100 µmol, 62%).

^{1}H NMR (400 MHz, $CDCl_3$): δ 7.89 – 7.82 (m, 2H), 7.66 (d, *J* = 2.0 Hz, 2H), 7.31 – 7.25 (m, 2H), 7.09 (d, *J* = 1.8 Hz, 2H), 4.17 (hept, *J* = 6.9 Hz, 2H), 3.77 (hept, *J* = 6.8 Hz, 2H), 1.46 (s, 18H), 1.40 – 1.32 (m, 30H), 1.07 (d, *J* = 6.7 Hz, 12H).

^{11}B{^{1}H} NMR (96 MHz, $CDCl_3$): δ –1.0.

^{13}C{^{1}H} NMR (101 MHz, $CDCl_3$): δ 144.0, 140.0, 135.7, 134.4, 130.6, 130.6, 122.7, 117.5, 114.5, 107.8, 99.6, 54.5, 53.9, 35.1, 35.0, 31.8, 29.9, 21.5, 20.3.

^{19}F NMR (282 MHz, $CDCl_3$): δ –153.8.

IR (ATR, neat) [cm^{-1}]: $\tilde{\nu}$ = 2955, 2906, 2871, 1864, 1569, 1480, 1418, 1359, 1265, 1052, 886, 818, 732, 551, 506, 405.

HR-MS-ESI(+) *m/z* calcd. for $C_{49}H_{72}AsN_4O_2^+$ [M-BF_4]$^+$ 823.4866, found 823.4865.

Anal. calcd. for $C_{49}H_{72}AsBF_4N_4O_2$: C 64.61, H 7.97, N 6.15 found: C 64.39, H 8.16, N 6.05.

m.p. 140 °C (decomp.).

Generation of radical **9**

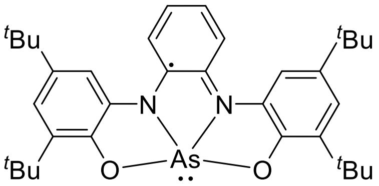

In a nitrogen-filled glovebox, potassium arsoranide **3** (10.0 mg, 9.97 µmol, 1.00 equiv.) was dissolved in *o*DCB (1 mL) and $AgHCB_{11}H_5Cl_6$ (4.5 mg, 9.8 µmol, 0.99 equiv.) was added. The mixture was stirred for 5 min at room temperature and filtered. The resulting filtrate was analyzed by X-band EPR spectroscopy at ambient temperature.

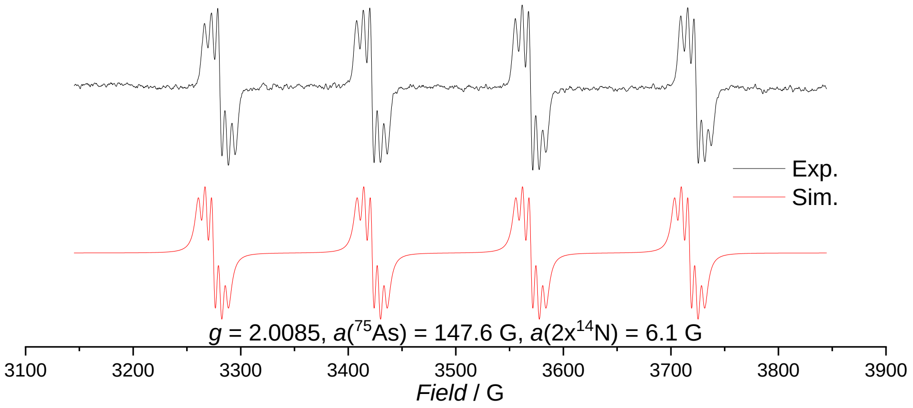

Figure S1 EPR spectrum and simulation parameters of **9** (*o*-DCB, 298 K).

Synthesis of As(III)/(V) heterodimer **10**

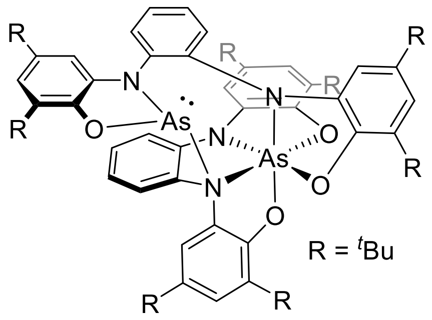

In a nitrogen-filled glovebox, arsenium triflate **5** (100 mg, 136 µmol, 1.00 equiv.) was dissolved in MeCN (1.5 mL) and a suspension of potassium arsoranide **3** (136 mg, 136 µmol, 1.00 equiv.) in MeCN (1.5 mL) was added. The resulting mixture was stirred for 30 min at ambient temperature. Afterwards, the formed suspension was filtered and the residue washed with MeCN (5 × 1 mL). Drying of the residue *in vacuo* delivered the title compound as a fine yellow solid (108 mg, 92.0 µmol, 68%).

^{1}H NMR (400 MHz, CD_2Cl_2, 233 K): δ 8.04 (s, 1H), 7.54 (d, *J* = 8.3 Hz, 1H), 7.27 (s, 1H), 7.11 – 6.97 (m, 3H), 6.79 (s, 1H), 6.77 – 6.64 (m, 5H), 6.50 (d, *J* = 8.1 Hz, 1H), 6.42 (t, *J* = 7.8 Hz, 1H), 6.21 (s, 1H), 5.90 (t, *J* = 7.8 Hz, 1H), 1.52 (s, 9H), 1.34 (s, 9H), 1.27 (s, 9H), 1.23 (s, 9H), 1.21 (s, 9H), 1.19 (s, 9H), 1.14 (s, 9H), 1.05 (s, 9H).

^{13}C{^{1}H} NMR (101 MHz, CD_2Cl_2, 233 K): δ 150.4, 148.4, 143.9, 143.4, 141.9, 141.6, 141.6, 141.5, 140.9, 135.6, 135.6, 133.7, 133.7, 132.8, 132.7, 132.0, 131.8, 131.6, 129.7, 129.2, 128.9, 126.5, 125.2, 124.3, 122.4, 121.0, 117.4, 115.9, 115.3, 115.1, 115.0, 113.5, 108.2, 107.8, 107.6, 106.3, 34.8, 34.6, 34.5, 34.5, 34.5, 34.4, 34.3, 34.3, 31.2 (*superposition of two signals*), 31.2, 31.0, 29.6, 29.2, 28.5, 28.4.

IR (ATR, neat) [cm^{-1}]: $\tilde{\nu}$ = 2953, 2905, 2868, 1581, 1481, 1417, 1360, 1226, 1102, 996, 824, 741, 687, 612, 547, 499, 450.

MS-LIFDI(toluene**)** *m*/*z* = 587.4 (100%, $[0.5\cdot M]^+$), 1174.3 (29%, $[M]^+$).

Anal. calcd. for $C_{68}H_{88}As_2N_4O_4$: C 69.49, H 7.55, N 4.77 found: C 68.90, H 7.52, N 4.66.

m.p. 208 °C (decomp.).

Identification of **10** from mixtures of radical **9**

By oxidation of Arsoranide **3**:

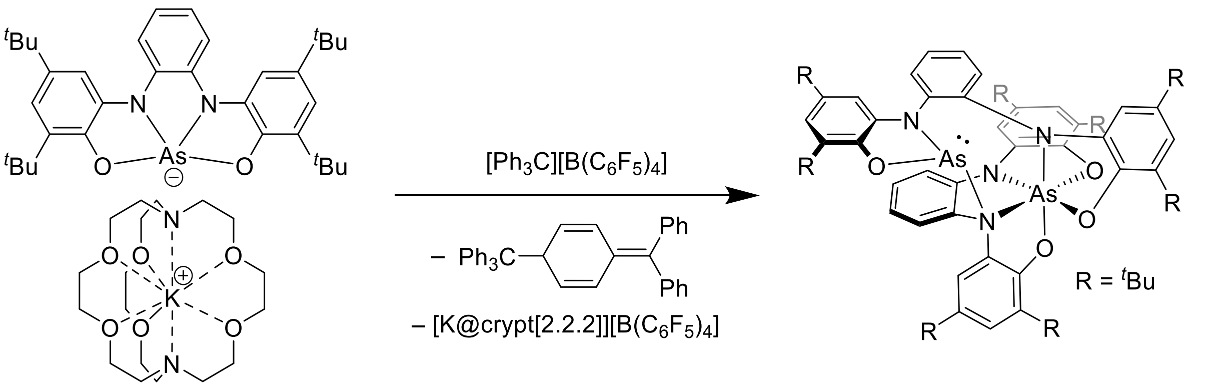

In a nitrogen-filled glovebox, **3** (10.0 mg, 10.0 µmol, 1.00 equiv.) was dissolved in DCM (0.5 mL) and a solution of trityl tetrakis(pentafluorophenyl)borate (9.2 mg, 10 µmol, 1.0 equiv.) in DCM (0.5 mL) was added. After stirring for 10 min, the solvent was evaporated and the residue extracted with pentane (3 × 0.5 mL). The filtrate was evaporated and the residue redispersed in MeCN. Et_2O was added dropwise until a homogeneous solution was obtained. Slow evaporation of this solution led to crystallization of **10** as its Et_2O solvate.

By reduction of Arsenium triflate **5**:

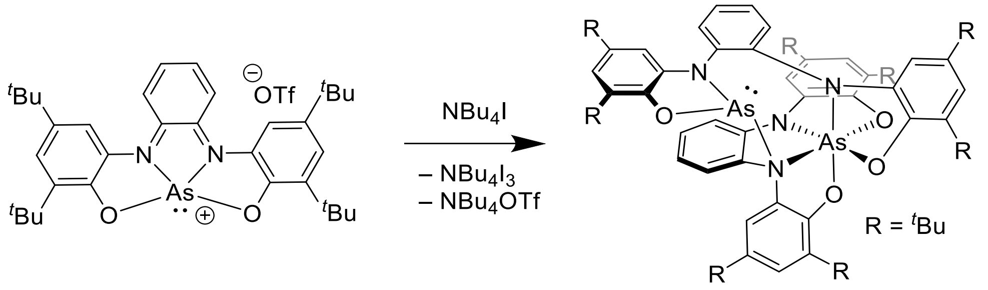

In a nitrogen-filled glovebox, **5** (50.0 mg, 67.9 µmol, 1.00 equiv.) was dissolved in DCM (1 mL). A solution of NBu_4I (37.6 mg, 102 µmol, 1.50 equiv.) in DCM (1 mL) was added leading to an immediate color change to deep-orange. After stirring for 10 min, the solution was evaporated and the residue was extracted with pentane (3 × 1 mL). The yellow filtrate was evaporated and the residue redispersed in MeCN. DCM was added dropwise until a homogeneous solution was obtained. Slow evaporation of this solution led to crystallization of **10** as its MeCN solvate.

Detection of **9** in solutions of **10**

In a nitrogen-filled glovebox, **10** (1.3 mg, 1.1 µmol) was dissolved in 1,2-dichlorobenzene (0.1 mL). The solution was transferred to an EPR tube fitted with a Teflon cap. The sample was analyzed by X-band EPR spectroscopy at ambient temperature. The obtained spectrum validates the presence of **9**.

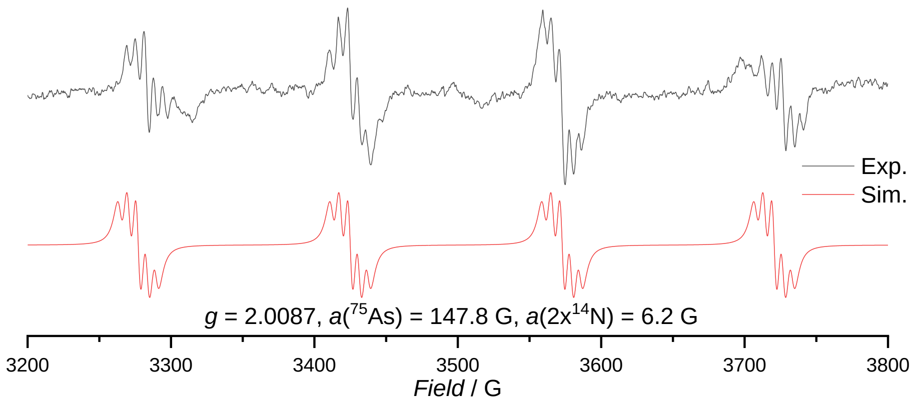

Figure S2 EPR spectrum of a solution of **10** (*o*DCB, 298 K).

Variable Temperature NMR of **10**

In a nitrogen-filled glovebox, **10** (15.0 mg, 12.8 µmol) was dissolved in tetrachlorethane-d_2 (0.6 mL) and hexamethylbenzene was added as an internal standard. The mixture was allowed to equilibrate at room temperature for 16 h and inserted into a 400 MHz NMR spectrometer. The relaxation delay was set to d1 = 10 s to ensure quantitative integration of the obtained ^1H NMR signals. A series of 5 ^1H NMR spectra were recorded at the following temperatures: 25 °C, 45 °C, 65 °C, 45 °C, 25 °C. Before measuring each spectrum, the sample was allowed to equilibrate at the given temperature for at least 60 min. The obtained spectra attest that **10** is in a reversible equilibrium with at least another species (Figure S3).

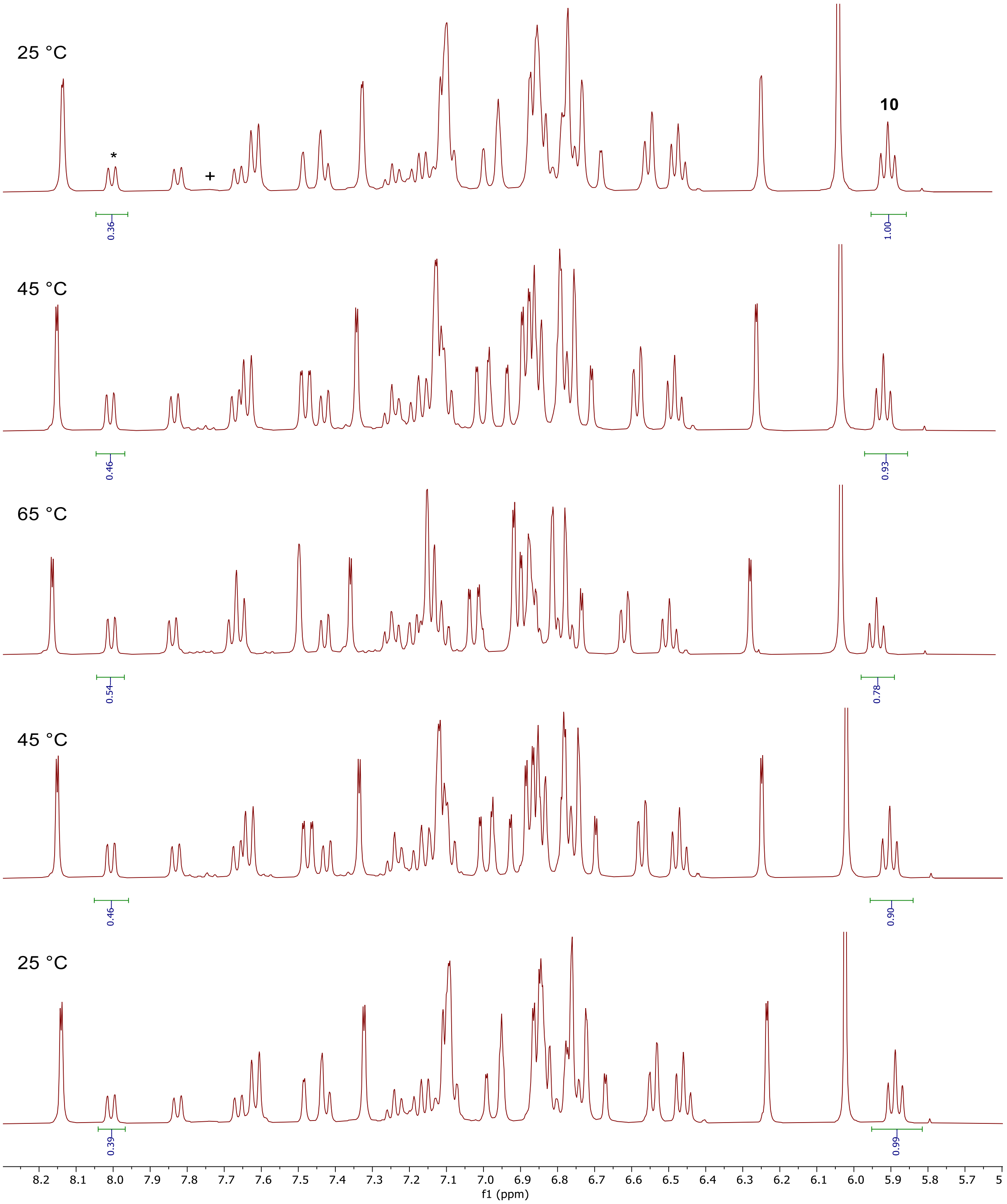

Figure S3 Variable Temperature ^1H NMR spectra of **10**. Only diagnostic region is shown. *, + indicate unidentified species. Integrals referenced against the internal standard C_6Me_6.

DOSY NMR of **10**

In a nitrogen-filled glovebox, **10** (15.0 mg, 12.8 µmol) was dissolved in dichloromethane-d_2 (0.6 mL) and adamantane was added as an internal standard. The mixture was allowed to equilibrate at room temperature for 16 h and submitted to a diffusion-ordered NMR spectroscopy (DOSY) experiment. Diffusion experiments were performed with the pulse program dstebpgp3s[4] (double-stimulated echo sequence in combination with bipolar gradient pulses) employing a linear gradient ramp incremented from 2 to 98% of the maximum gradient strength. To achieve ideal signal attenuation the gradient pulses were adjusted by recording and comparing 1D spectra (dstebpgp3s1d). The spectra were processed using MestreNova 14.1 and TopSpin 4.0.7.

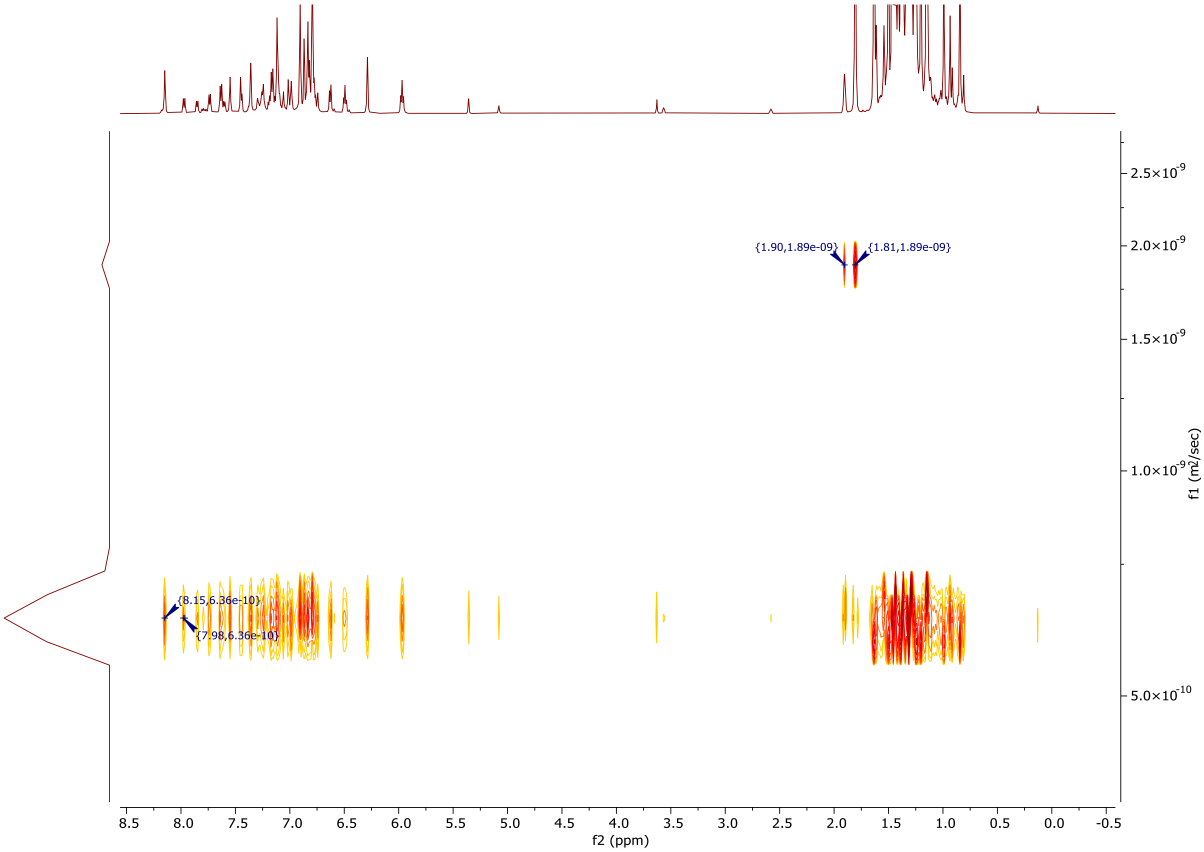

Figure S4 DOSY ^{1}H NMR spectra of **10** with the internal standard adamantane in CD_2Cl_2.

The diffusion coefficients of **10** and the species (isomer) it is in equilibrium with are determined as $6.36 \cdot 10^{-10}$ m^2s^{-1}, the one of adamantane as $1.89 \cdot 10^{-9}$ m^2s^{-1}. Exchange Spectroscopy (EXSY) shows that the equal diffusion coefficients of **10** and its isomer are not a result of dynamic interconversion on the NMR timescale by the absence of off-diagonal exchange peaks.

The diffusion coefficients were used to determine the molecular weight of the species under investigation using the ECC-MW estimation software by Stalke and co-workers.[5] By using the merged external calibration curve, a molecular weight of 1131 g mol^{-1} was determined which agrees well with the molecular weight of **10** (1175 g mol^{-1}, 4% relative deviation).

Attempted Trapping of **9**

In a nitrogen-filled glovebox, a precooled (–40 °C) solution of **3** (20.0 mg, 19.9 µmol, 1.00 equiv.) in CD_2Cl_2 (0.6 mL) was added to solid 2,2,6,6-tetramethyl-1-oxopiperidin-1-ium tetrafluoroborate (4.8 mg, 20 µmol, 1.0 equiv.). After warming to room temperature, a ^{1}H NMR spectrum was recorded. No TEMPO-adduct of **9** could be detected, instead the formation of **10** (and isomers) is observed. This is in agreement with the computed thermodynamics (*vide infra*).

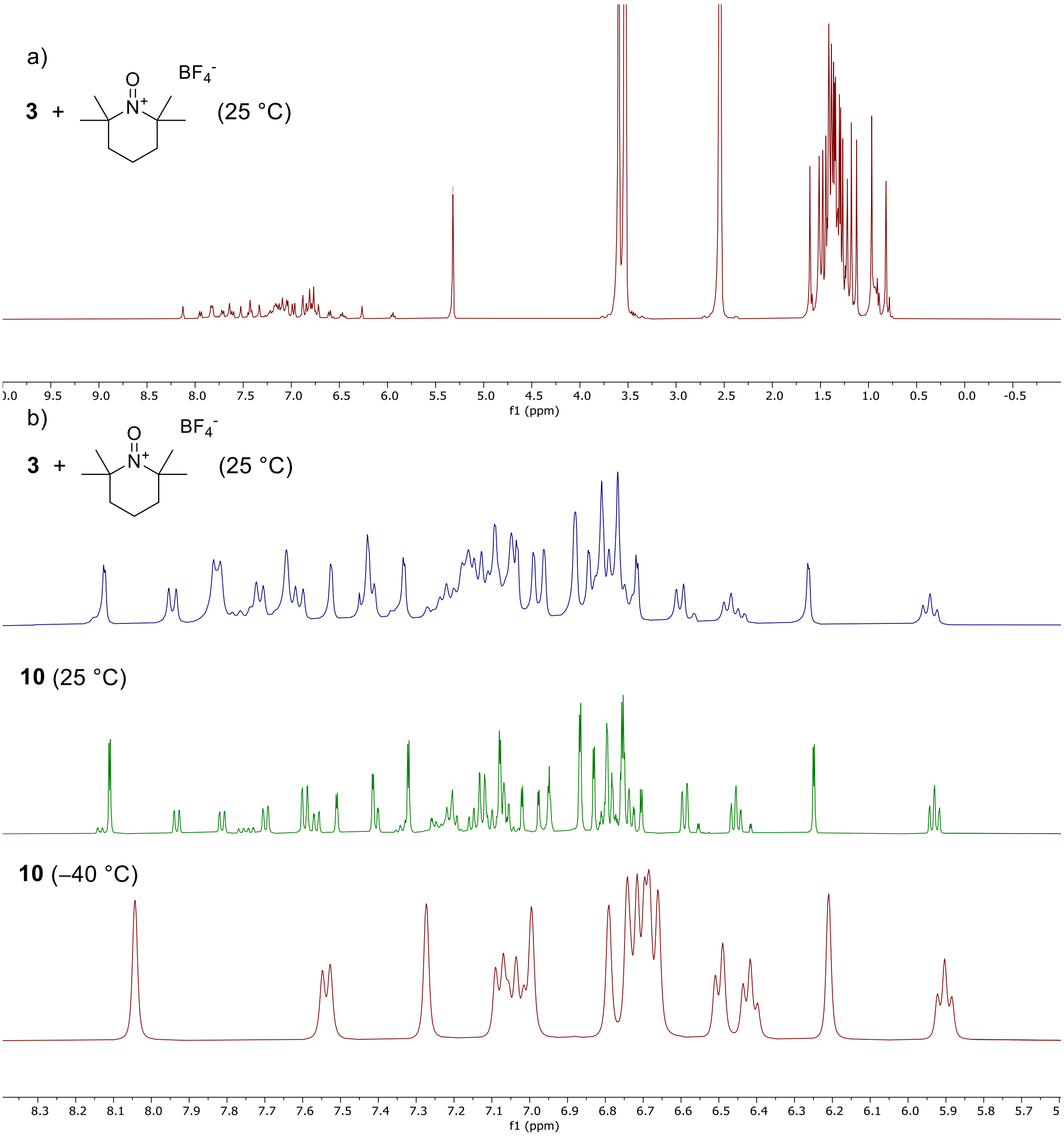

Figure S5 a) Crude 1H NMR spectrum of the described reaction. b) Comparison of the diagnostic aromatic region.

Reaction of **5** with Ph_4PCl

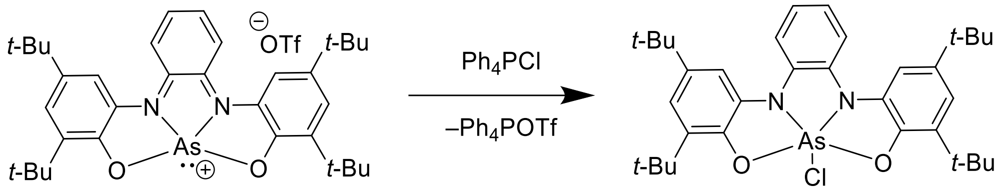

In a Schlenk flask equipped with a magnetic stirring bar, arsenium triflate **5** (20.0 mg, 27.2 µmol, 1.00 equiv.) was dissolved in DCM (0.5 mL). Tetraphenylphosphonium chloride (11.2 mg, 29.9 µmol, 1.10 equiv.) in DCM (0.5 mL) was added resulting in an immediate color change from dark green to yellow-orange. After stirring for 5 min at ambient temperature, the solvent was removed and the residue extracted with pentane (3 × 1 mL). Drying of the filtrate yielded chloroarsorane **4** as a yellow solid (16.9 mg, 27.1 µmol, >99%).

Reaction of **6** with $HNTf_2$

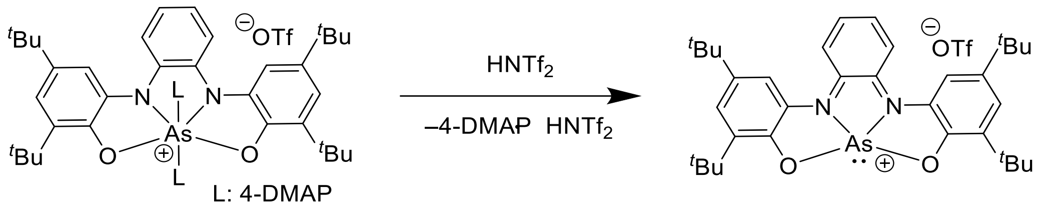

In a nitrogen-filled glovebox, an NMR tube fitted with a Teflon cap was charged with a solution of **6** (10.0 mg, 10.2 µmol, 1.00 equiv.) in $CDCl_3$ (0.5 mL) and the internal standard 1,2-DCE. After recording an ^1H NMR spectrum, a solution of $HNTf_2$ (14.3 mg, 50.9 µmol, 4.99 equiv.) in $CDCl_3$ (0.5 mL) was added resulting in a color change from yellow to deep-green. After 20 min, ^1H NMR analysis of the mixture evidenced the formation of **5** in a spectroscopic yield of 62%.

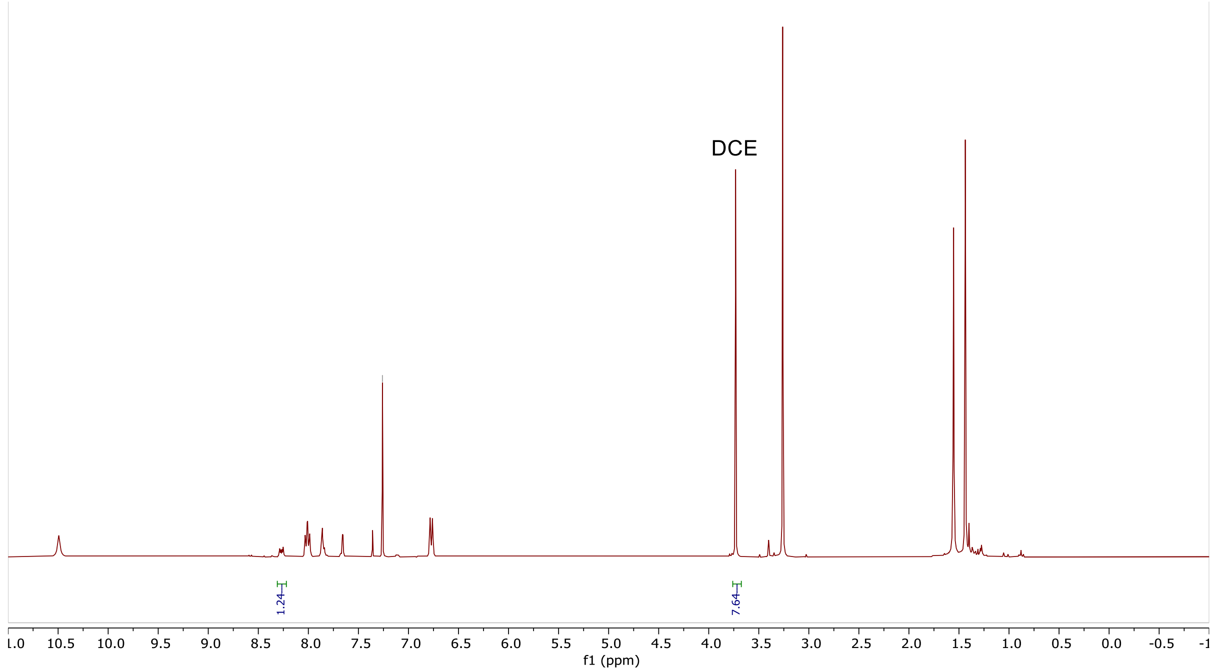

Figure S6 Crude ^1H NMR spectrum of the reaction of **6** with $HNTf_2$ after 20 min.

Transfer Hydrogenation

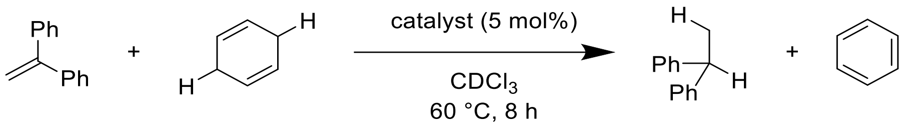

A solution of 1,1-diphenylethylene (18.0 mg, 100 µmol, 1.00 equiv.), 1,4-cyclohexadiene (16.0 mg, 200 µmol, 2.00 equiv.) and the internal standard 1,1,2,2-tetrachloroethane in $CDCl_3$ (0.6 mL) were added to the corresponding catalyst (5.0 µmol, 5.0 mol%). The sample was transferred to an NMR tube fitted with a Teflon cap and inserted into an NMR spectrometer preheated to 60 °C. The reaction progress was monitored by quantitative ^{1}H NMR spectroscopy over the course of 8 h. The yield was determined by integration of the 1,1-diphenylethane signals which are literature reported.[6]

catalyst	*m*(catalyst) / mg	Yield after 8h / %
**5**	3.7	96
**7**	4.3	<1
$HNTf_2$	1.4	>99

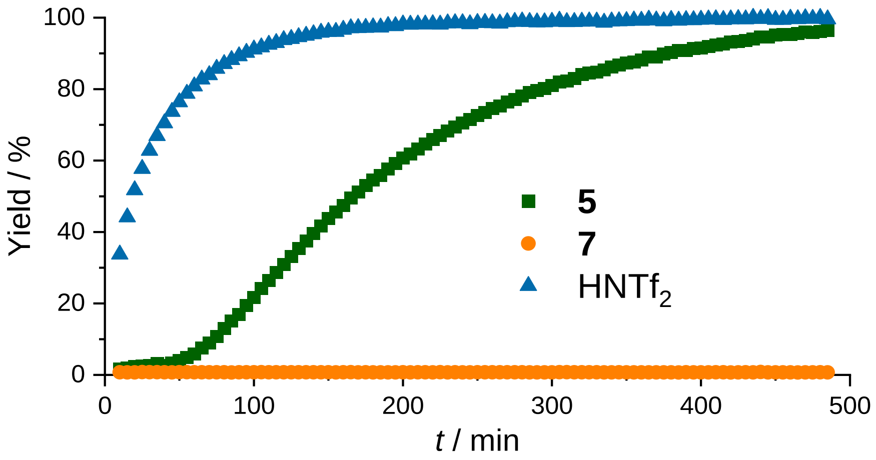

Figure S7 Yield-time profile of the described transfer hydrogenation using different catalysts.

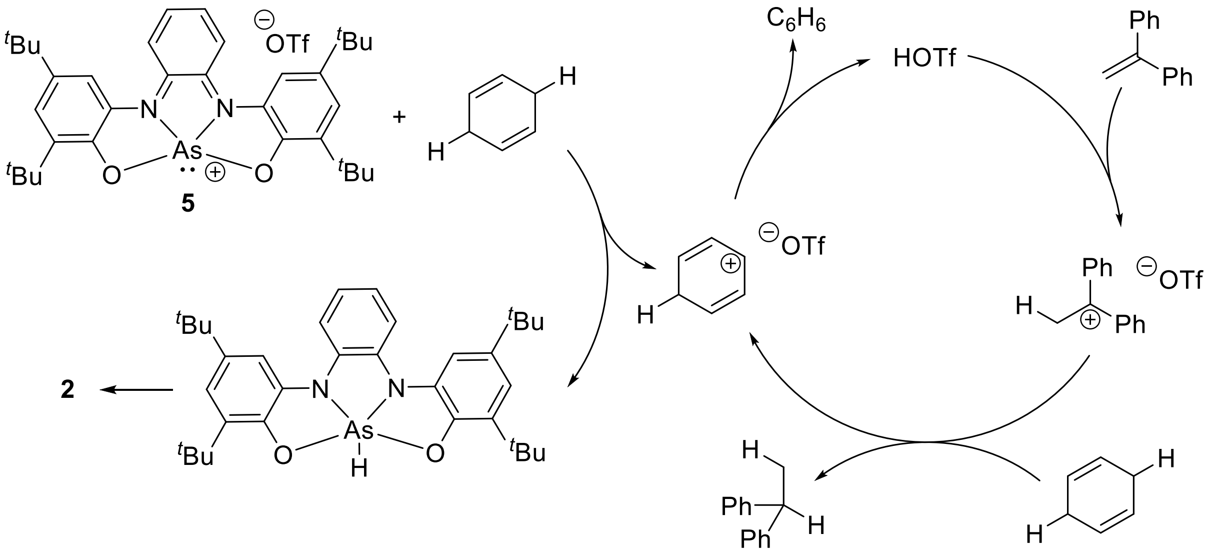

Scheme S1 Transfer hydrogenation: Literature-based mechanistic rational.[7]

Cyclic Voltammetry

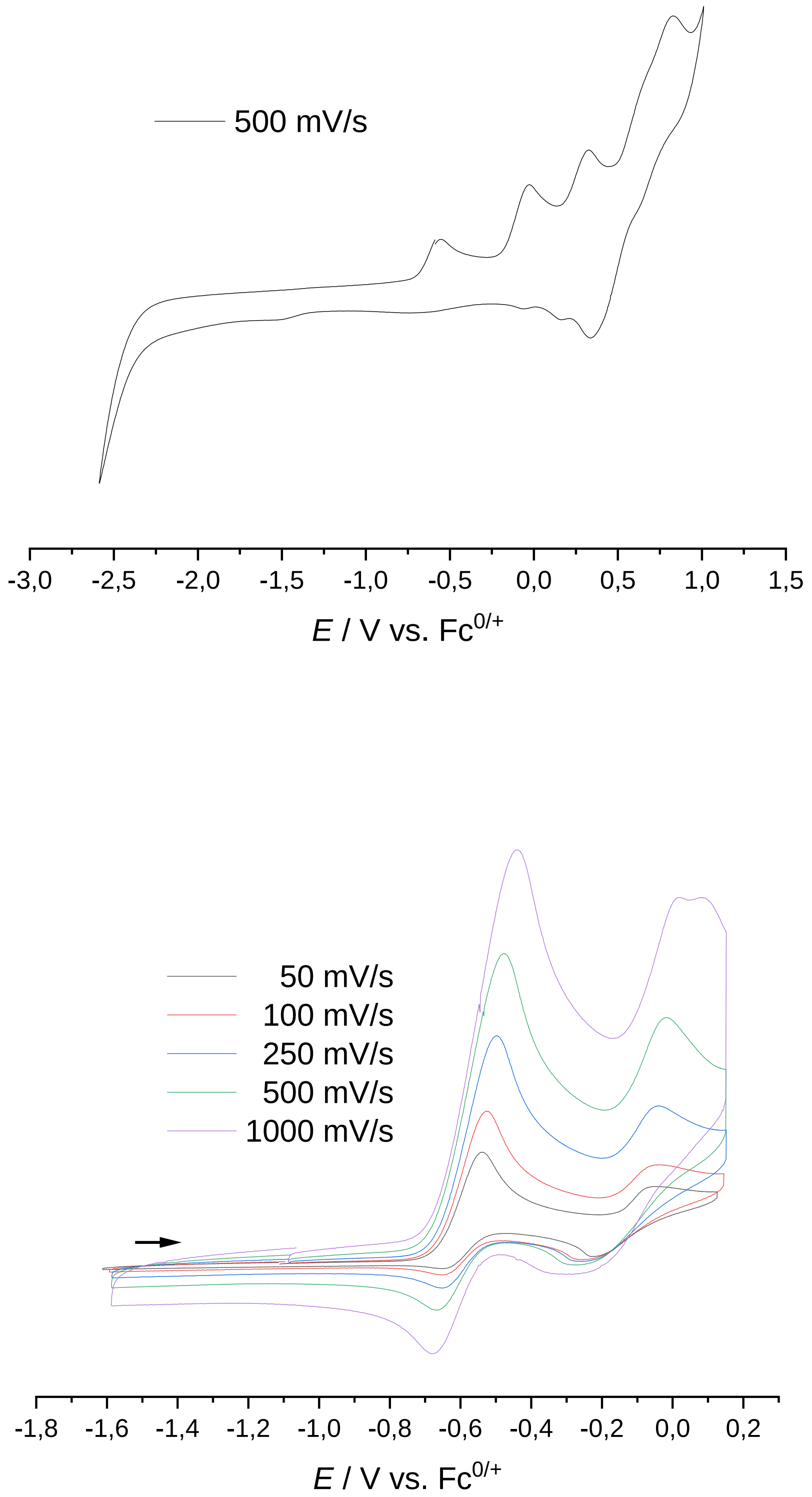

Figure S8 Cyclic voltammograms of arsoranide **3** (DCM, 0.1 M NBu_4OTf).

UV/vis Spectroscopy

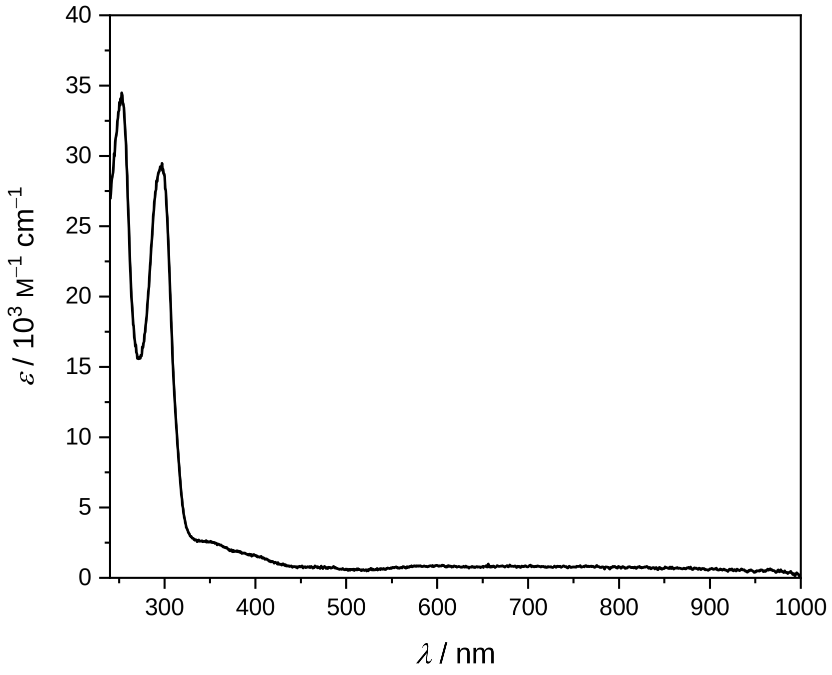

Figure S9 UV/vis spectrum of **4** in DCM (0.1 mM).

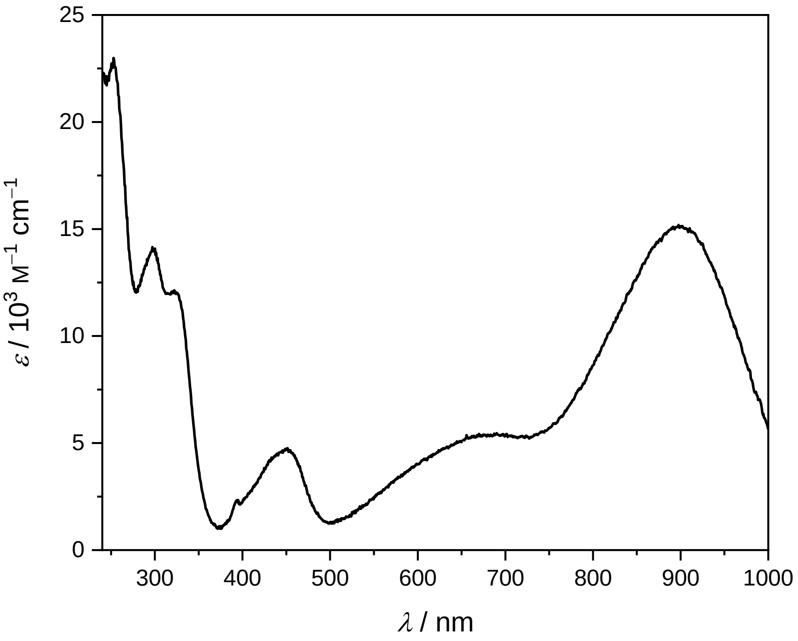

Figure S10 UV/vis spectrum of **5** in DCM (0.1 mM).

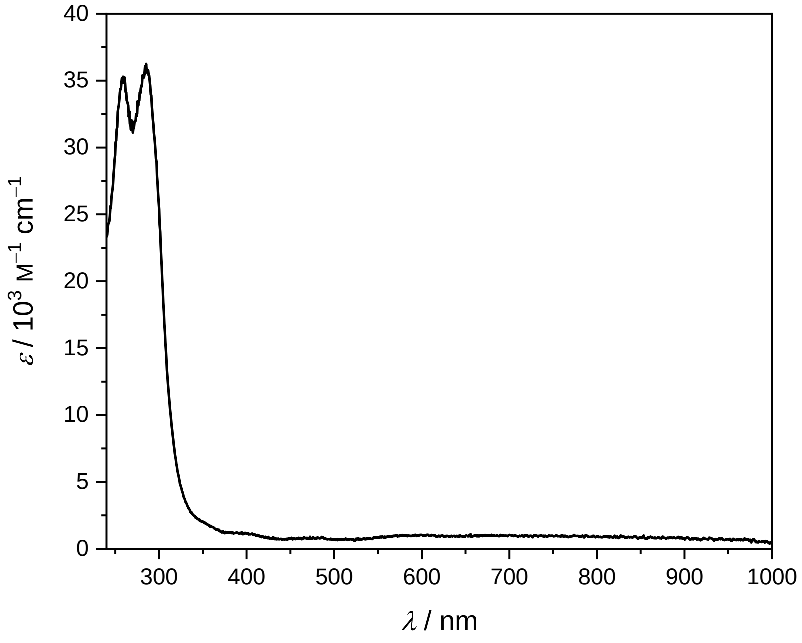

Figure S11 UV/vis spectrum of **6** in DCM (0.1 mM).

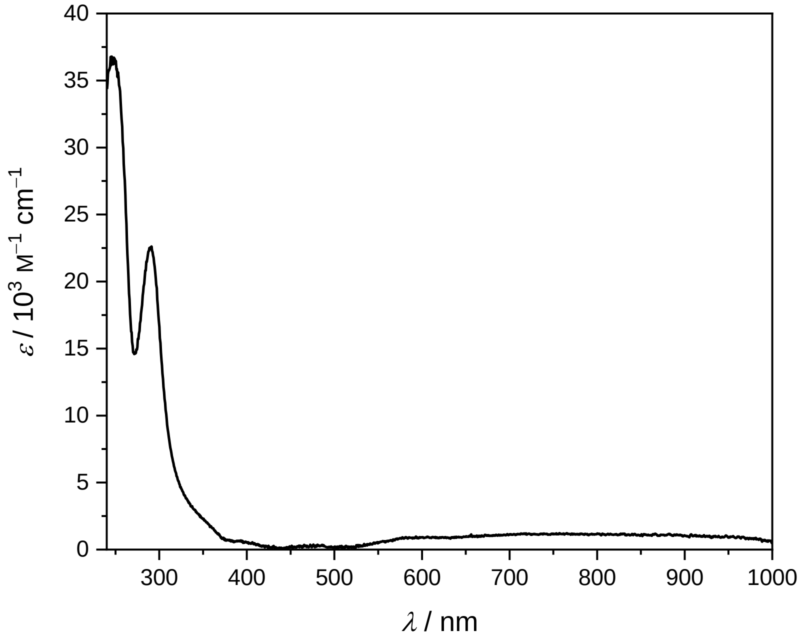

Figure S12 UV/vis spectrum of **7** in DCM (0.1 mM).

	λ_{max} / nm (ε / 10^3 m^{-1} cm^{-1})
**4**	253 (34.5), 297 (29.2)
**5**	252 (22.7), 298 (14.0), 323 (12.1), 448 (4.7), 683 (5.4), 898 (15.1)
**6**	259 (35.3), 286 (35.8)
**7**	248 (36.7), 290 (22.5)

Crystallographic supplement

General Information

Data collection was done on two dual source equipped *Bruker D8 Venture* four-circle-diffractometer from *Bruker AXS GmbH*; used X-ray sources: microfocus *IµS 2.0* Cu/Mo and microfocus *IµS 3.0* Ag/Mo from *Incoatec GmbH* with mirror optics *HELIOS* and single-hole collimator from *Bruker AXS GmbH;* used detector: *Photon III CE14* (Cu/Mo) and *Photon III HE* (Ag/Mo) from *Bruker AXS GmbH*.

Used programs: *APEX4 Suite* (v2022.1-1) for data collection and therein integrated programs *SAINT* V8.40A (Integration) and *SADABS* 2016/2 (Absorption correction) from *Bruker AXS GmbH*; structure solution was done with *SHELXT*, refinement with *SHELXL*-2018/3[8]; OLEX2[9] and FinalCif[10] were used for data finalization.

Special Utilities: *SMZ1270* stereomicroscope from *Nikon Metrology GmbH* was used for sample preparation; crystals were mounted on *MicroMounts* or *MicroLoops* from *MiTeGen* in NVH oil; crystals were cooled to given temperature with *Cryostream 800* from *Oxford Cryosystems.*

This supplement contains in the following the refinement detail tables, a figure of the complete asymmetric unit and a microscope picture of the crystal used for data collection. The picture of the single crystals was cropped and auto-adjusted for brightness, graduation curve and white balance in Adobe Photoshop (Version 24.1.0). Further crystallographic details can be obtained from the crystallographic information files (CIFs) uploaded to the *Cambridge Crystallographic Data Centre* (CCDC), where they can be obtained free of charge.

Identifier	CCDC number	Identifier	CCDC number
**2**	2416702	**7**	2416707
**3**	2416703	**8**	2416708
**4**	2416704	**10◦MeCN**	2416709
**5**	2416705	**10◦Et_2O**	2416710
**6**	2416706		

Refinement details for **2**

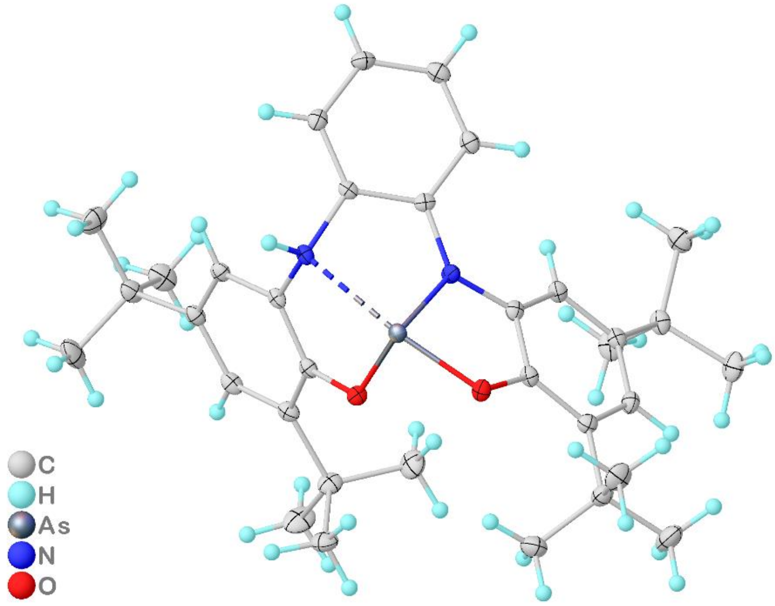

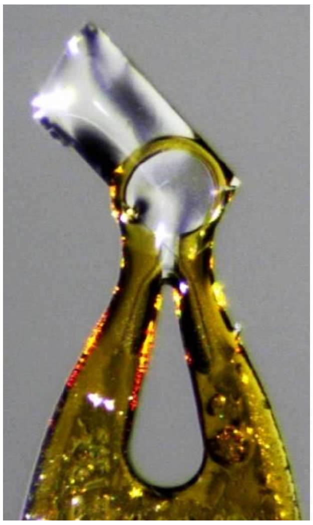

Figure S 13: Full asymmetric unit of **2**. Anisotropic displacement ellipsoids drawn at 50% probability level. The HN···As contact is drawn as stippled bond. Single crystals were obtained from a solution in pentane.

CCDC number	2416702
Empirical formula	$C_{34}H_{45}AsN_2O_2$
Formula weight	588.64
Temperature [K]	100.00
Crystal system	Triclinic
Space group (number)	$P\bar{1}$ (2)
a [Å]	10.216(5)
b [Å]	11.324(6)
c [Å]	15.373(7)
α [°]	109.210(14)
β [°]	101.629(18)
γ [°]	102.679(12)
Volume [Å^3]	1564.5(13)
Z	2
ρ_{calc} [gcm^{-3}]	1.250
μ [mm^{-1}]	1.118
F(000)	624
Crystal size [mm^3]	0.663×0.216×0.046
Crystal color	Colorless
Crystal shape	Plate
Radiation	MoK_α (λ=0.71073 Å)

2θ range [°]	4.00 to 57.53 (0.74 Å)
Index ranges	−13 ≤ h ≤ 12<br>−14 ≤ k ≤ 15<br>−20 ≤ l ≤ 20
Reflections collected	39907
Independent reflections	8093<br>R_{int} = 0.0521<br>R_{sigma} = 0.0399
Completeness to θ = 25.242°	99.9 %
Data / Restraints / Parameters	8093/0/368
Goodness-of-fit on F^2	1.093
Final R indexes [I≥2σ(I)]	R_1 = 0.0366<br>wR_2 = 0.0809
Final R indexes [all data]	R_1 = 0.0450<br>wR_2 = 0.0842
Largest peak/hole [eÅ^{-3}]	0.61/-0.66

Refinement details for **3**

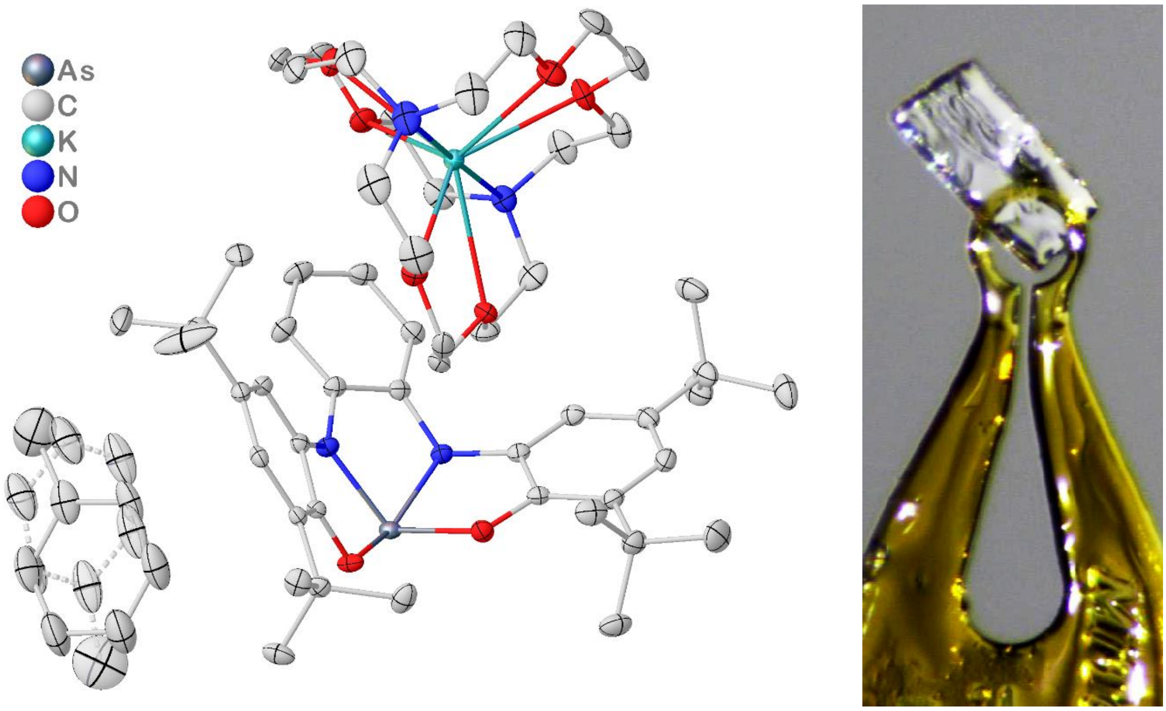

Figure S14: Full asymmetric unit of **3**◦toluene. Anisotropic displacement ellipsoids drawn at 50% probability level. Hydrogen atoms omitted for clarity. The co-crystalized toluene exhibits positional disorder with the minor part having an occupancy of 0.367(5) and being drawn translucent with stippled bonds. Single crystal of **3**◦toluene were obtained from a solution in toluene. It should be noted, that another monoclinic polymorph of this compound was observed after crystallization from toluene that actually forms the majority of the crystalline material. However, due to the poor crystal quality no reliable data could be obtained for them.

CCDC number	2416703
Empirical formula	$C_{59}H_{88}AsKN_4O_8$
Formula weight	1095.35
Temperature [K]	100.00
Crystal system	Orthorhombic
Space group (number)	$Pbca$ (61)
a [Å]	17.8184(9)
b [Å]	21.4034(11)
c [Å]	31.6665(18)
α [°]	90
β [°]	90
γ [°]	90
Volume [Å^3]	12076.8(11)
Z	8
ρ_{calc} [gcm^{-3}]	1.205
μ [mm^{-1}]	0.685
F(000)	4688
Crystal size [mm^3]	0.366×0.174×0.064
Crystal color	Colorless
Crystal shape	Plate
Radiation	MoK_α (λ=0.71073 Å)

2θ range [°]	3.93 to 61.11 (0.70 Å)
Index ranges	−23 ≤ h ≤ 25<br>−27 ≤ k ≤ 30<br>−44 ≤ l ≤ 45
Reflections collected	233136
Independent reflections	18441<br>R_{int} = 0.0589<br>R_{sigma} = 0.0269
Completeness to θ = 25.242°	99.9 %
Data / Restraints / Parameters	18441/118/736
Goodness-of-fit on F^2	1.087
Final *R* indexes [*I*≥2σ(*I*)]	R_1 = 0.0409<br>wR_2 = 0.0958
Final *R* indexes [all data]	R_1 = 0.0511<br>wR_2 = 0.1005
Largest peak/hole [eÅ^{-3}]	0.55/-1.04

Refinement details for **4**

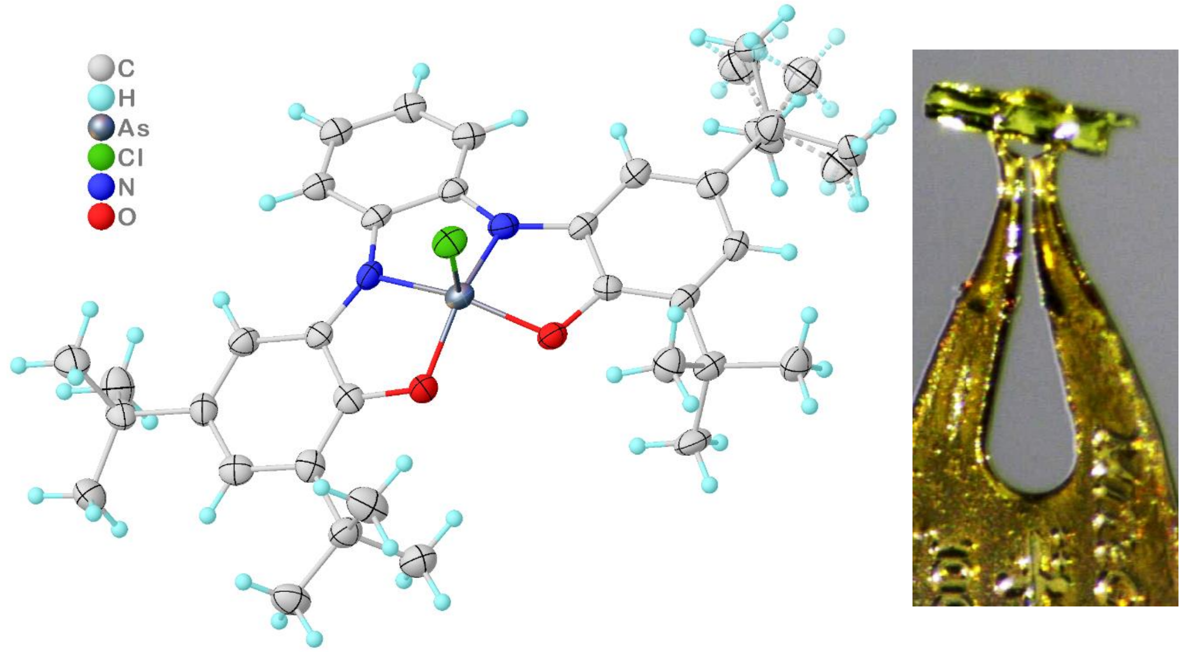

Figure S15: Molecular structure of **4** in the solid state. The asymmetric unit consists of five molecules of **4** with variably disordered *tert*-butyl groups and two molecules of pentane of which one also exhibits positional disorder. Minor disorder part drawn translucent with stippled bonds. Anisotropic displacement ellipsoids drawn at 50% probability level. Furthermore, non-merohedral twinning was found for the crystal. Two unit cells were indexed, which relate by the operation (1 0 0 | 0.017 -0.999 0.086 | 0 0 1) and the refinement against hklf5 data yielded a batch scale factor of 0.4014(10). Crystals were obtained from a solution in pentane.

CCDC number	2416704
Empirical formula	$C_{36}H_{48.80}AsClN_2O_2$
Formula weight	651.94
Temperature [K]	100.00
Crystal system	Triclinic
Space group (number)	$P1$ (1)
a [Å]	16.545(3)
b [Å]	16.722(2)
c [Å]	17.379(3)
α [°]	87.845(3)
β [°]	89.700(4)
γ [°]	65.373(4)
Volume [Å^3]	4367.5(12)
Z	5
ρ_{calc} [gcm^{-3}]	1.239
μ [mm^{-1}]	1.081
F(000)	1724
Crystal size [mm^3]	0.42×0.092×0.078
Crystal color	Yellow
Crystal shape	Plate
Radiation	MoK_{α} (λ=0.71073 Å)

2θ range [°]	3.63 to 55.99 (0.76 Å)
Index ranges	−21 ≤ h ≤ 21<br>−22 ≤ k ≤ 22<br>−22 ≤ l ≤ 22
Reflections collected	41444
Independent reflections	41444<br>R_{int} = 0.0808<br>R_{sigma} = 0.0626
Completeness to θ = 25.242°	99.9 %
Data / Restraints / Parameters	41444/131/2190
Goodness-of-fit on F^2	1.116
Final *R* indexes [I≥2σ(I)]	R_1 = 0.0625<br>wR_2 = 0.1520
Final *R* indexes [all data]	R_1 = 0.0787<br>wR_2 = 0.1648
Largest peak/hole [eÅ^{-3}]	1.04/-1.08
Flack X parameter	-0.019(6)

Refinement details for **5**

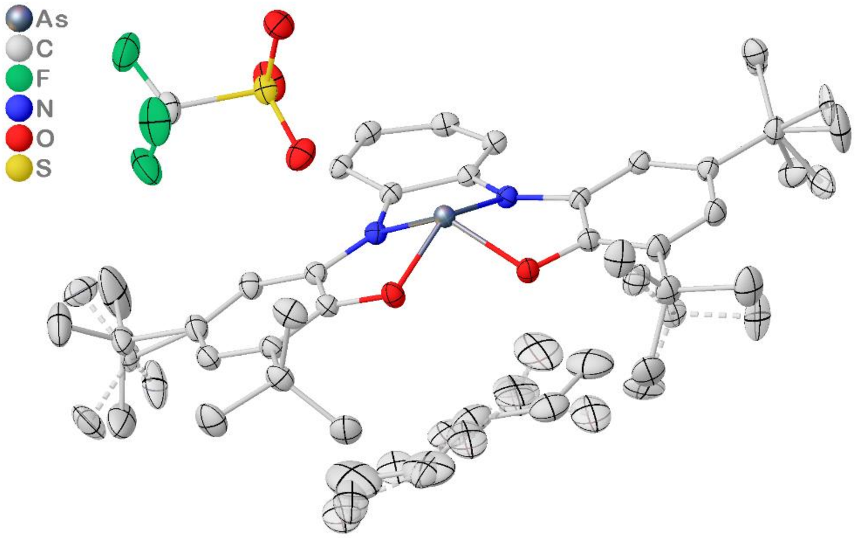

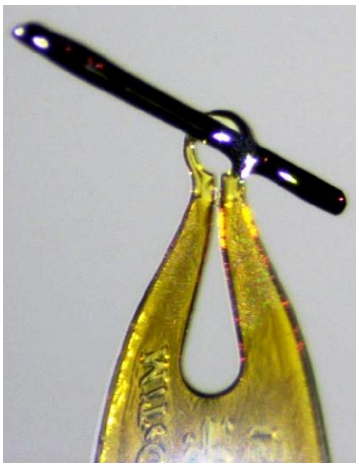

Figure S16: Full asymmetric unit of **5**◦pentane. Anisotropic displacement ellipsoids drawn at 50% probability level. Hydrogen atoms omitted for clarity. Three *tert*-butyl groups exhibit rotational disorder and the co-crystallized pentane is disordered over (at least) three positions. Minor disorder parts are drawn translucent with stippled bonds. Single crystals of **5**◦pentane were obtained from a mixture of dichloromethane and pentane.

CCDC number	2416705
Empirical formula	$C_{40}H_{56}AsF_3N_2O_5S$
Formula weight	808.84
Temperature [K]	100.00
Crystal system	Monoclinic
Space group (number)	$P2_1/c$ (14)
a [Å]	8.8339(9)
b [Å]	18.5422(19)
c [Å]	25.291(2)
α [°]	90
β [°]	98.855(3)
γ [°]	90
Volume [$Å^3$]	4093.2(7)
Z	4
ρ_{calc} [gcm^{-3}]	1.313
μ [mm^{-1}]	0.938
F(000)	1704
Crystal size [mm^3]	1.255×0.087×0.073
Crystal color	Green
Crystal shape	Needle
Radiation	MoK_α (λ=0.71073 Å)

2θ range [°]	3.93 to 61.12 (0.70 Å)
Index ranges	−12 ≤ h ≤ 11<br>−26 ≤ k ≤ 26<br>−36 ≤ l ≤ 35
Reflections collected	143037
Independent reflections	12547<br>R_{int} = 0.0573<br>R_{sigma} = 0.0245
Completeness to θ = 25.242°	100.0 %
Data / Restraints / Parameters	12547/540/687
Goodness-of-fit on F^2	1.042
Final *R* indexes [$I \geq 2\sigma(I)$]	R_1 = 0.0316<br>wR_2 = 0.0779
Final *R* indexes [all data]	R_1 = 0.0398<br>wR_2 = 0.0824
Largest peak/hole [$eÅ^{-3}$]	0.57/-0.52

Refinement details for **6**

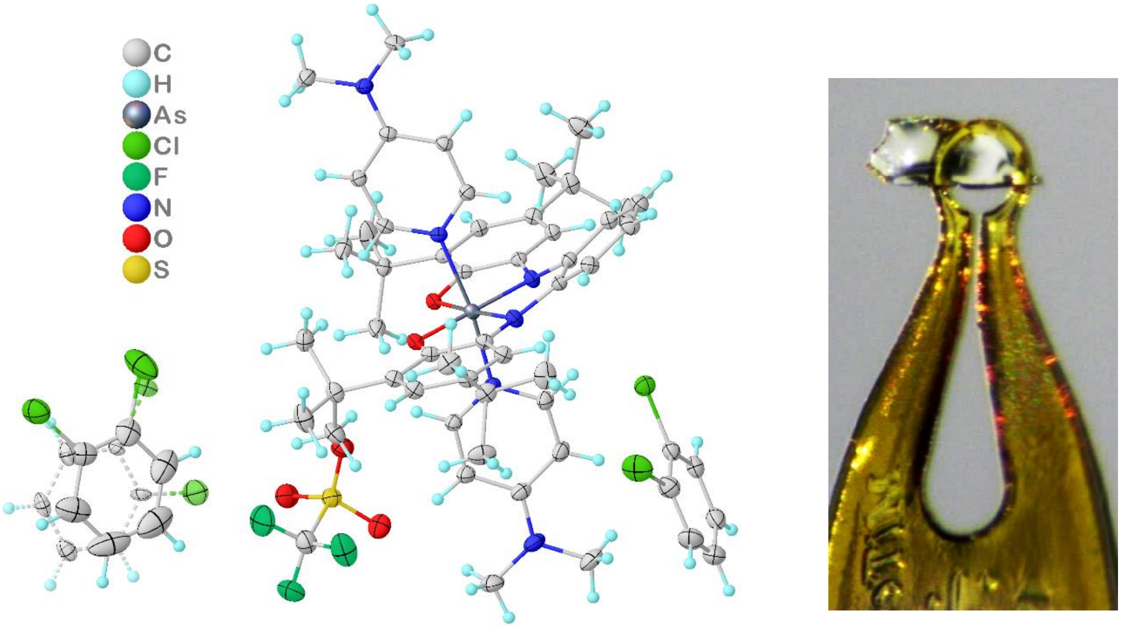

Figure S17: Full asymmetric unit of **6**◦2 dichlorobenzene. Anisotropic displacement ellipsoids drawn at 50% probability level. One of the two molecules of co-crystallized dichlorobenzene exhibits positional disorder. The minor disorder part is drawn translucent with stippled bonds. Single crystals of **6**◦2 dichlorobenzene were obtained from a solution in dichlorobenzene.

CCDC number	2416706
Empirical formula	$C_{61}H_{72}AsCl_4F_3N_6O_5S$
Formula weight	1275.02
Temperature [K]	100.00
Crystal system	Triclinic
Space group (number)	$P\overline{1}$ (2)
a [Å]	14.087(3)
b [Å]	15.217(3)
c [Å]	15.703(4)
α [°]	66.163(7)
β [°]	79.280(7)
γ [°]	85.812(6)
Volume [Å^3]	3025.1(13)
Z	2
ρ_{calc} [gcm^{-3}]	1.400
μ [mm^{-1}]	0.836
F(000)	1328
Crystal size [mm^3]	0.316×0.155×0.06
Crystal color	Colorless
Crystal shape	Plate
Radiation	MoK_α (λ=0.71073 Å)
2θ range [°]	3.75 to 59.30 (0.72 Å)

Index ranges	−19 ≤ h ≤ 19<br>−21 ≤ k ≤ 21<br>−21 ≤ l ≤ 21
Reflections collected	211342
Independent reflections	17049<br>R_{int} = 0.0678<br>R_{sigma} = 0.0288
Completeness to θ = 25.242°	99.9 %
Data / Restraints / Parameters	17049/66/795
Absorption correction T_{min}/T_{max} (method)	0.6127/0.7459 (multi-scan)
Goodness-of-fit on F^2	1.047
Final *R* indexes [I≥2σ(I)]	R_1 = 0.0389<br>wR_2 = 0.1002
Final *R* indexes [all data]	R_1 = 0.0494<br>wR_2 = 0.1067
Largest peak/hole [eÅ^{-3}]	0.68/−0.87

Refinement details for **7**

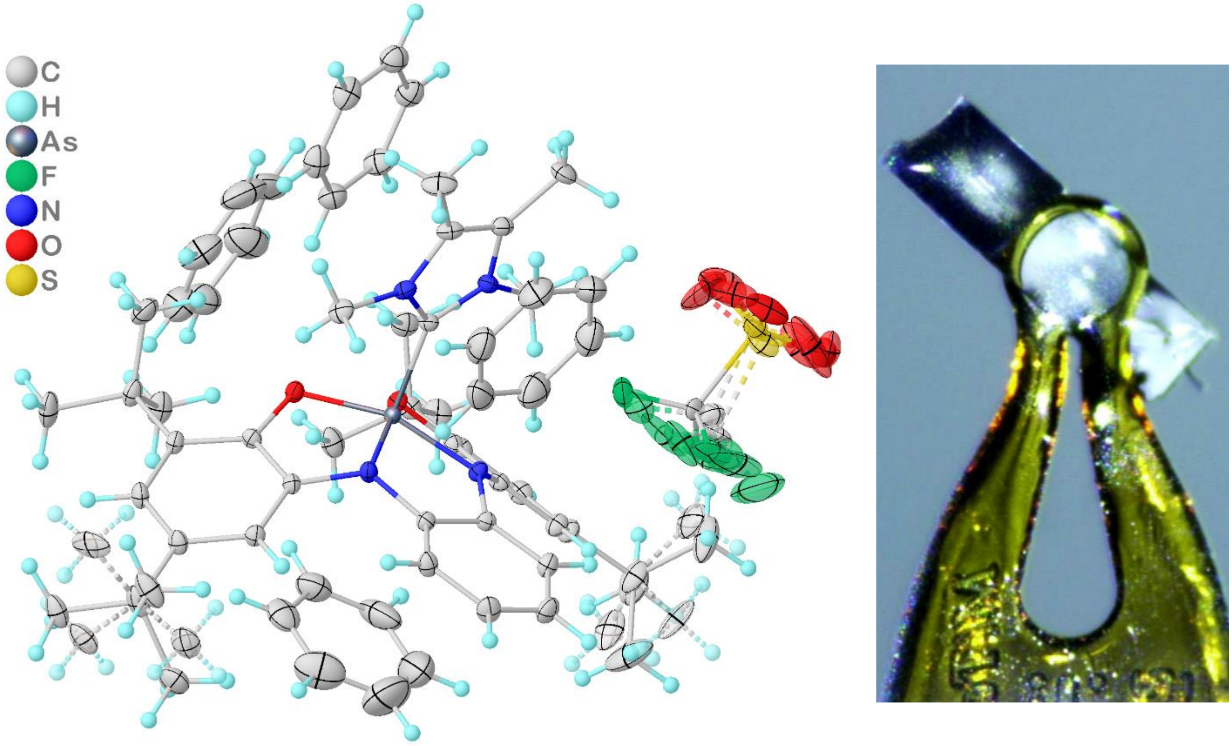

Figure S18: Full asymmetric unit of **7**◦4 benzene. Anisotropic displacement ellipsoids drawn at 50% probability level. Two of the *tert*-butyl groups exhibit rotational disorder and the triflate anion was split over three position due to severe disorder. The minor disorder parts are drawn translucent with stippled bonds. Single crystals of **7**◦4 benzene were obtained from a solution in benzene.

CCDC number	2416707
Empirical formula	$C_{66}H_{80}AsF_3N_4O_5S$
Formula weight	1173.32
Temperature [K]	100.00
Crystal system	Orthorhombic
Space group (number)	$Pbca$ (61)
a [Å]	20.1992(10)
b [Å]	16.8225(8)
c [Å]	36.311(2)
α [°]	90
β [°]	90
γ [°]	90
Volume [Å^3]	12338.5(11)
Z	8
ρ_{calc} [gcm^{-3}]	1.263
μ [mm^{-1}]	0.646
F(000)	4960
Crystal size [mm^3]	0.759×0.184×0.06
Crystal color	Colorless
Crystal shape	Plate
Radiation	MoK_{α} (λ=0.71073 Å)
2θ range [°]	4.03 to 59.15 (0.72 Å)

Index ranges	−28 ≤ h ≤ 27<br>−23 ≤ k ≤ 23<br>−50 ≤ l ≤ 50
Reflections collected	296158
Independent reflections	17301<br>R_{int} = 0.0741<br>R_{sigma} = 0.0280
Completeness to θ = 25.242°	99.9 %
Data / Restraints / Parameters	17301/295/947
Absorption correction T_{min}/T_{max} (method)	0.7653/0.9841 (numerical)
Goodness-of-fit on F^2	1.037
Final *R* indexes [$I \geq 2\sigma(I)$]	R_1 = 0.0422<br>wR_2 = 0.0874
Final *R* indexes [all data]	R_1 = 0.0594<br>wR_2 = 0.0955
Largest peak/hole [eÅ^{-3}]	0.55/−0.54
Extinction coefficient	0.00062(4)

Refinement details for **8**

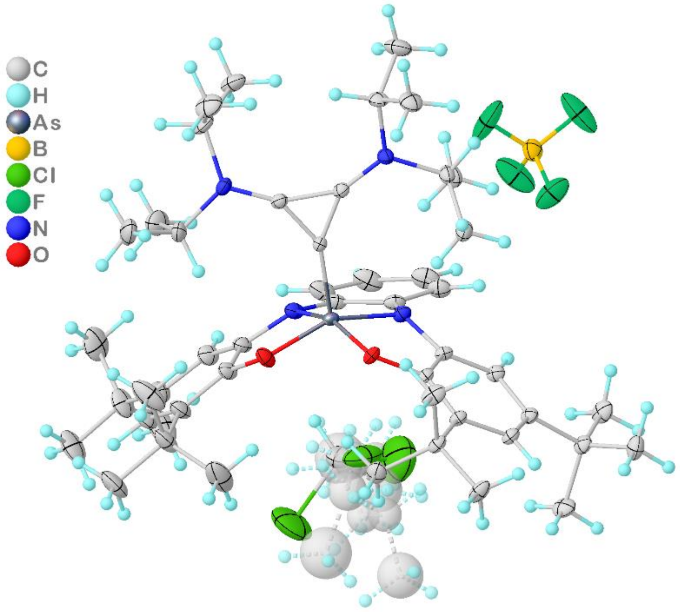

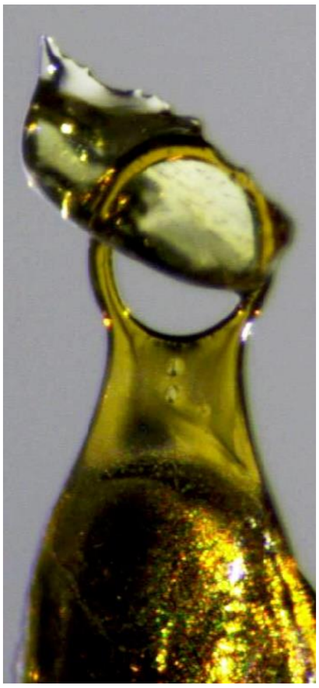

Figure S19: Full asymmetric unit of **8**◦0.29 $CHCl_3$, 0.71 pentane. Anisotropic and isotropic displacement ellipsoids drawn at 50% probability level. The co-crystallized solvents occupy a mixed cavity, with one position of chloroform and two positions refined with pentane molecules. Due to the proximity of chlorine and carbon atoms in this disorder, the refinement of anisotropic carbon atoms was found to be too unstable, instead these were refined only isotopically. The disordered pentanes are drawn translucent with stippled bonds. Single crystals of **8**◦0.29 $CHCl_3$, 0.71 pentane were obtained from a mixture of chloroform and pentane.

CCDC number	2416708
Empirical formula	$C_{52.84}H_{80.81}AsBCl_{0.87}F_4N_4O_2$
Formula weight	996.74
Temperature [K]	100.00
Crystal system	Monoclinic
Space group (number)	$C2/c$ (15)
a [Å]	39.2197(18)
b [Å]	14.3822(5)
c [Å]	19.6749(10)
α [°]	90
β [°]	95.803(2)
γ [°]	90
Volume [Å^3]	11041.1(8)
Z	8
ρ_{calc} [gcm^{-3}]	1.199
μ [mm^{-1}]	0.712
F(000)	4245
Crystal size [mm^3]	0.588×0.234×0.232
Crystal color	Yellow
Crystal shape	Block
Radiation	MoK_α (λ=0.71073 Å)
2θ range [°]	3.61 to 61.12 (0.70 Å)

Index ranges	$-55 \leq h \leq 56$<br>$-20 \leq k \leq 17$<br>$-28 \leq l \leq 28$
Reflections collected	159333
Independent reflections	16891<br>R_{int} = 0.0507<br>R_{sigma} = 0.0242
Completeness to θ = 25.242°	100.0 %
Data / Restraints / Parameters	16891/96/693
Absorption correction T_{min}/T_{max} (method)	0.6506/1.0000 (numerical)
Goodness-of-fit on F^2	1.028
Final R indexes [$I \geq 2\sigma(I)$]	R_1 = 0.0356<br>wR_2 = 0.0902
Final R indexes [all data]	R_1 = 0.0432<br>wR_2 = 0.0952
Largest peak/hole [eÅ^{-3}]	0.79/−0.64

Refinement details for **10** (two solvates)

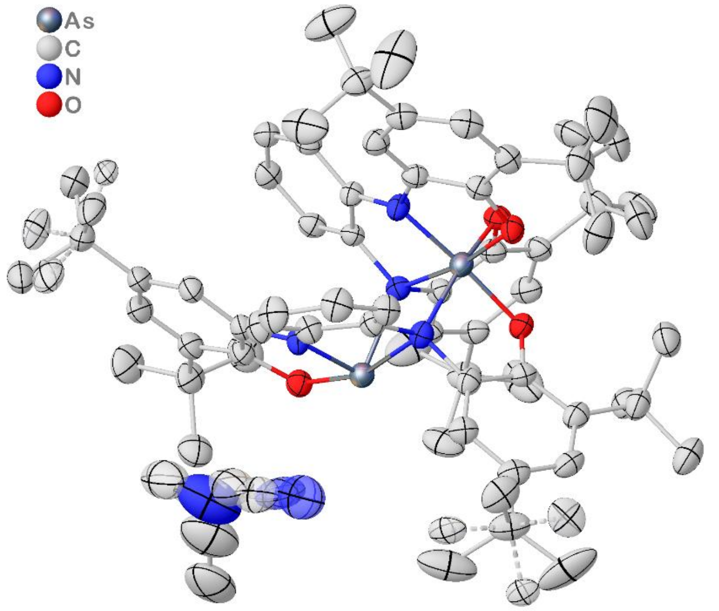

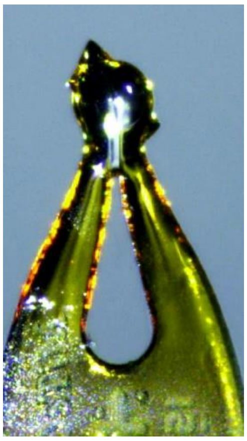

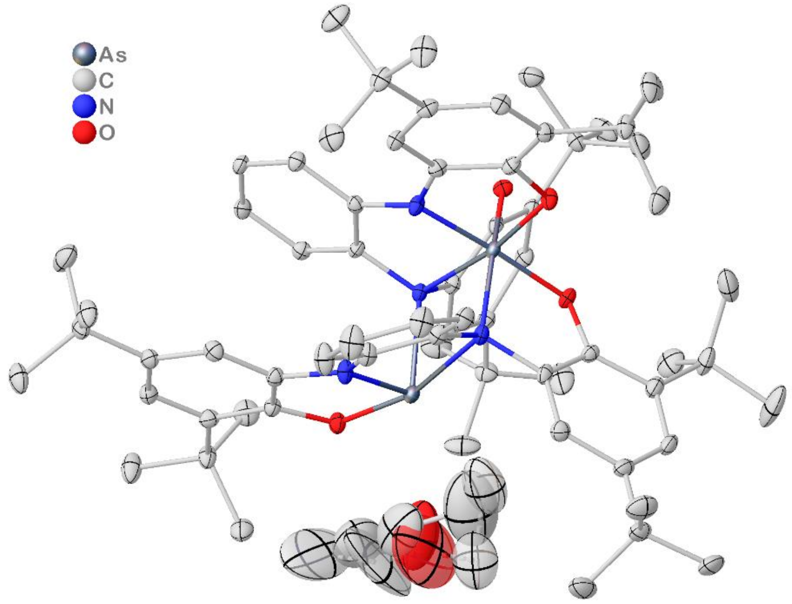

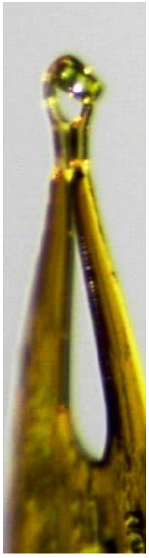

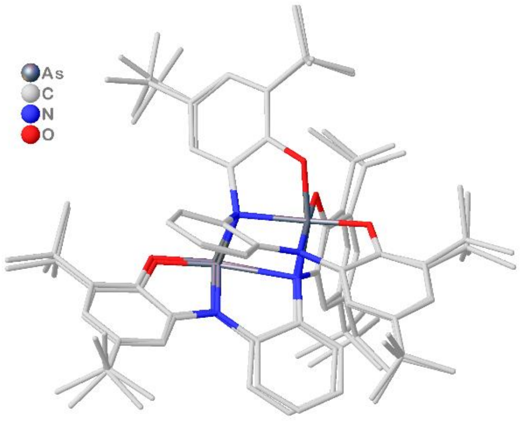

Figure S20: Two solvates of compound **10** could be crystallized. **10**◦MeCN (top): Full asymmetric unit is shown; hydrogen atoms are omitted for clarity. Anisotropic displacement ellipsoids drawn at 50% probability level. Two of the *tert*-butyl groups exhibit rotational disorder and the acetonitrile molecule was split over three position due to severe disorder. The acetonitrile refines better when the sum of occupancies is below one, maybe due to loss of solvent from the lattice. The minor disorder parts are drawn translucent with stippled bonds. Single crystals of **10**◦MeCN were obtained from a solution in acetonitrile. **10**◦Et_2O (bottom): Full asymmetric unit is shown; hydrogen atoms are omitted for clarity. Anisotropic displacement ellipsoids drawn at 50% probability level. The co-crystallized diethyl ether is disorder over two positions. The minor disorder part is drawn translucent with stippled bonds. Single crystals of **10**◦ Et_2O were obtained from a solution in diethyl ether. Side: Overlay of molecule **10** in both solvates reveal only minor conformational differences, mainly in the peripheral groups.

	**10**◦MeCN	**10**◦ Et_2O
CCDC number	2416710	2416709
Empirical formula	$C_{69.56}H_{90.34}As_2N_{4.78}O_4$	C72H98As2N4O5
Formula weight	1207.28	1249.38
Temperature [K]	100.00	100.00
Crystal system	Monoclinic	Triclinic
Space group (number)	$P2_1/n$ (14)	$P\bar{1}$ (2)
a [Å]	15.406(5)	14.084(3)
b [Å]	24.196(13)	15.603(3)
c [Å]	17.640(8)	18.674(3)
α [°]	90	70.606(6)
β [°]	98.49(2)	71.048(4)
γ [°]	90	63.453(4)
Volume [$Å^3$]	6504(5)	3386.3(10)
Z	4	2
ρ_{calc} [gcm^{-3}]	1.233	1.225
μ [mm^{-1}]	1.078	1.038
F(000)	2557	1328
Crystal size [mm^3]	0.42×0.229×0.024	0.067×0.09×0.12
Crystal color	Colorless	Yellow
Crystal shape	Plate	Block
Radiation	MoK_α (λ=0.71073 Å)	MoKα (λ=0.71073 Å)
2θ range [°]	4.10 to 52.88 (0.80 Å)	4.20 to 56.66 (0.75 Å)
Index ranges	−19 ≤ h ≤ 17<br>−30 ≤ k ≤ 28<br>−21 ≤ l ≤ 22	−18 ≤ h ≤ 18<br>−20 ≤ k ≤ 20<br>−24 ≤ l ≤ 24
Reflections collected	74596	205356
Independent reflections	13345<br>R_{int} = 0.0775<br>R_{sigma} = 0.0654	16858<br>R_{int} = 0.0902<br>R_{sigma} = 0.0433
Completeness to θ = 25.242°	99.9 %	99.9 %
Data / Restraints / Parameters	13345/79/867	16858 / 54 / 821
Absorption correction T_{min}/T_{max} (method)	0.1940/0.2369 (multi-scan)	0.7813 / 1.0000 (numerical)
Goodness-of-fit on F^2	1.014	1.070
Final *R* indexes [$I \geq 2\sigma(I)$]	R_1 = 0.0483<br>wR_2 = 0.1089	R_1 = 0.0364<br>wR_2 = 0.0803
Final *R* indexes [all data]	R_1 = 0.0868<br>wR_2 = 0.1262	R_1 = 0.0537<br>wR_2 = 0.0869
Largest peak/hole [$eÅ^{-3}$]	0.49/−0.59	0.60/−0.62

Metrical Analysis of Compounds **4-8**

Figure S21 summarizes metrical data of the compounds **4-8** relevant to the ligand oxidation state. Metrical oxidation states (MOS) of the amidophenolate side arms were computed using the method described by Brown.[11]

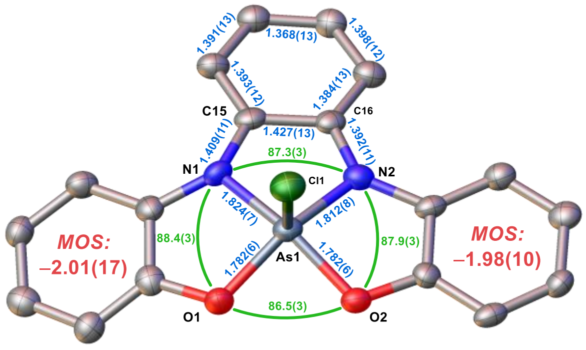

4

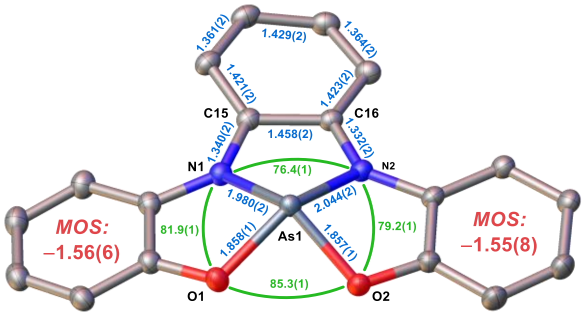

5

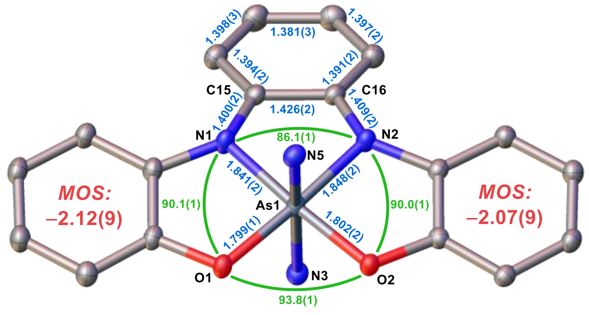

6

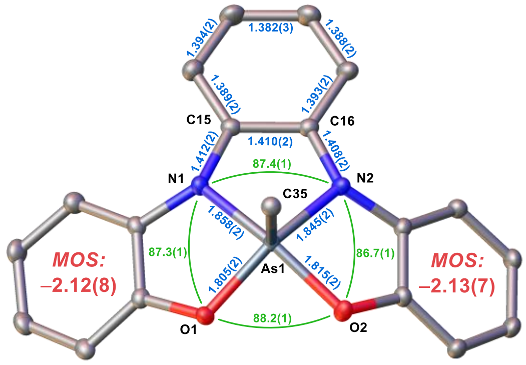

7

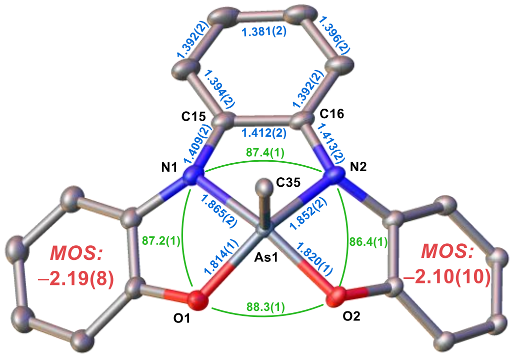

8

Figure S21 Simplified solid-state structures of compounds **4**-**8**. Hydrogens, anions, solvent molecules and *tert*-butyl groups were omitted for clarity. Coordinated Lewis bases are reduced to its donor atom. Ellipsoids are shown at the 50% probability level.

Computational Details

General Information

Geometry optimization, frequency and single-point energy calculations were carried out with Gaussian16, Revision A.03[12]. Unless otherwise mentioned, the (U)B3LYP functional[13] was employed in combination with the def2-TZVP basis set[14] and D3 dispersion correction with Becke-Johnson damping[15]. For the creation of input files and analysis of calculation outcomes GaussView 6.0 was used. IBOview was used for visualization of molecular orbitals.[16] Stationary points were characterized by frequency evaluation. NBO charges were computed using the NBO 3.1 program as imbedded in Gaussian16, Revision A.03.

Thermochemistry for Formation of **6** from **5** and 4-DMAP

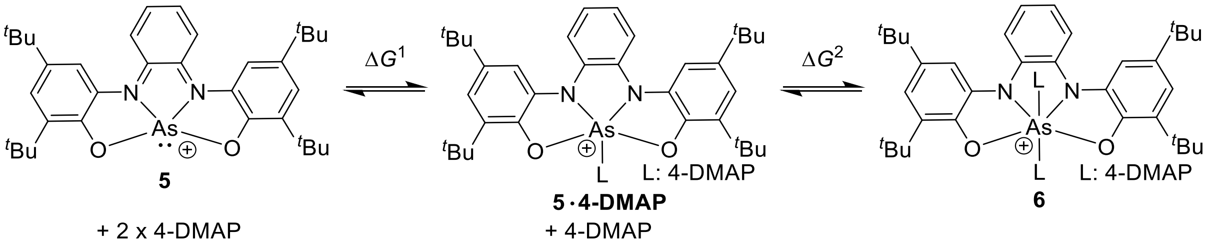

Scheme S2 4-DMAP Binding of ***5***.

The structures of **5⁺**, **5⁺ · 4-DMAP**, **6⁺** and 4-DMAP were optimized and their single point energies (*SPE*) calculated at the B3LYP-D3(BJ)/def2-TZVP level of theory. Thermodynamic corrections to the Gibbs Free Enthalpy (G_{corr}) were obtained from frequency calculations at the same level of theory. The data is summarized in Table S1.

Table S1 Thermochemistry of DMAP Binding to **5⁺**.

	SPE / E_h	G_{corr} / E_h	*SPE* + G_{corr} / E_h
**5⁺**	–3818.596717	0.637667	–3817.959050
**5⁺ · 4-DMAP**	–4201.065622	0.791071	–4200.274551
**6⁺**	–4583.552370	0.941240	–4582.611130
**4-DMAP**	–382.433929	0.128752	–382.305177

Accordingly, the values for ΔG^1 and ΔG^2 are –6.5 kcal mol^{-1} and –19.7 kcal mol^{-1}, respectively. As a consequence, the isolation of **5⁺ · 4-DMAP** is not feasible since its conversion to **5⁺** and **6⁺** is favorable by –13.2 kcal mol^{-1}.

Selected Molecular Orbitals and Natural Bond Orbitals of **5**

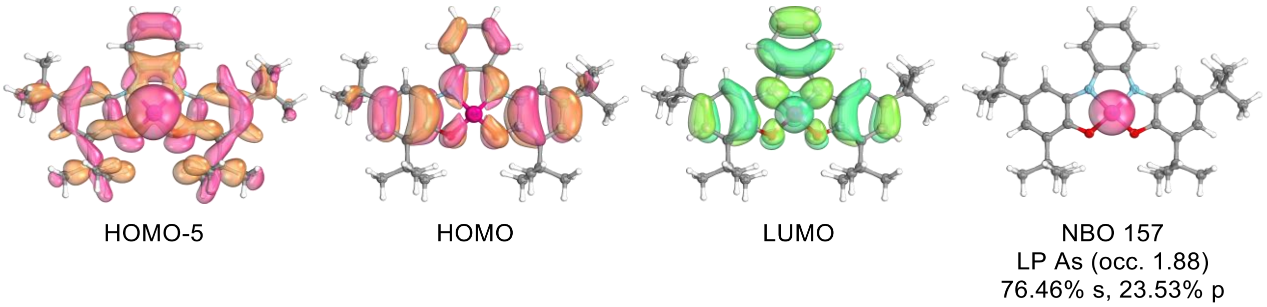

Figure S22 Selected Kohn-Sham molecular orbitals and natural bond orbitals of **5** calculated at the B3LYP-D3(BJ)/def2-TZVP level of theory.

Frontier Molecular Orbital Diagram of Lewis Base adducts of **5**

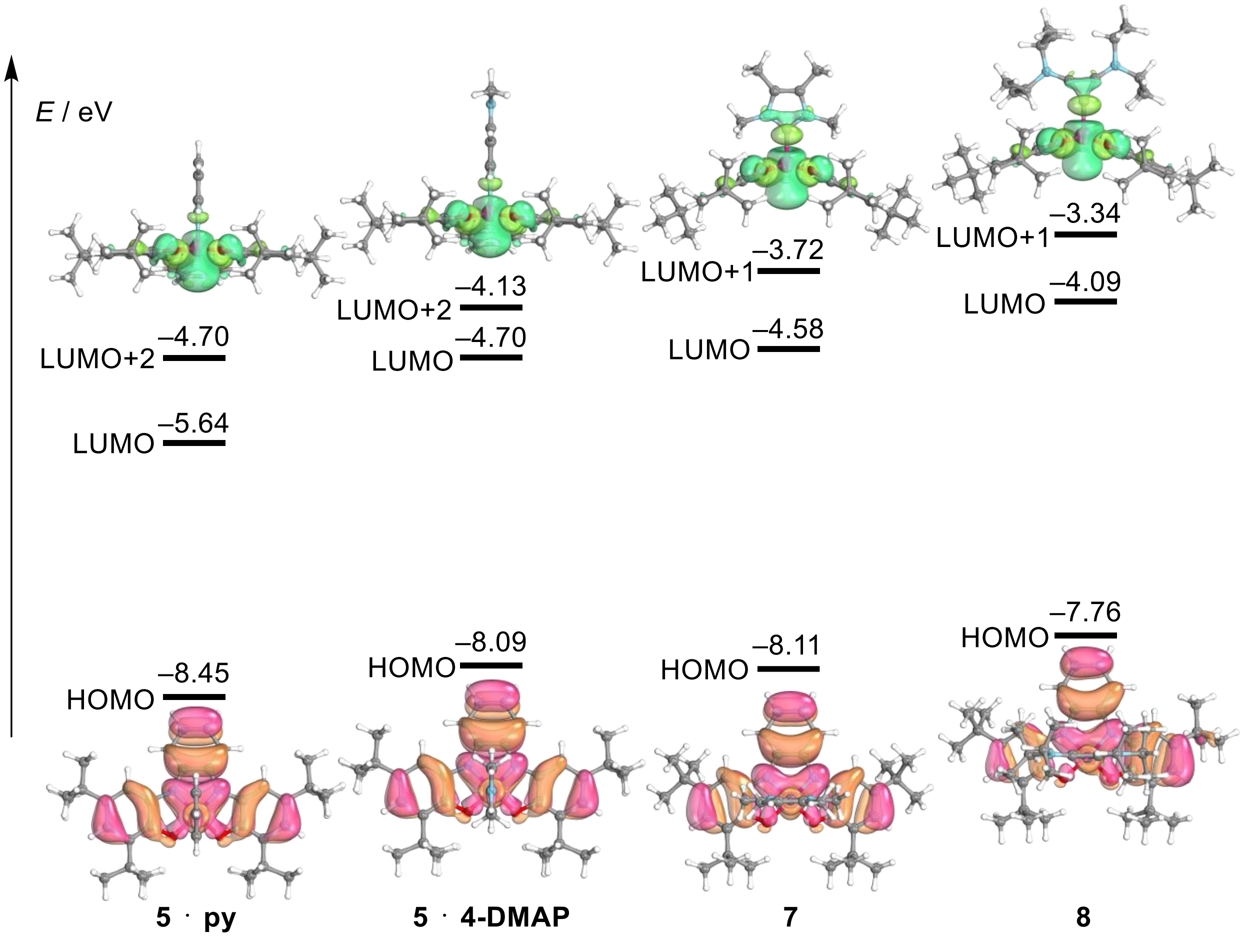

Figure S23 Frontier Kohn-Sham molecular orbitals for selected Lewis base adducts of **5** calculated at the B3LYP-D3(BJ)/def2-TZVP level of theory (py: pyridine).

Natural Resonance Theory

Natural Resonance Theory analysis was performed on 5 at the B3LYP-D3(BJ)/def2-TZVP level of theory using the NBO 6.0 program as imbedded in Gaussian16, Revision A.03.

All resonance structures are shown with relative weights equal to or exceeding 5.0%. The lone pair on As was present in all 26 resonance structures with more than 0.01% relative weight.

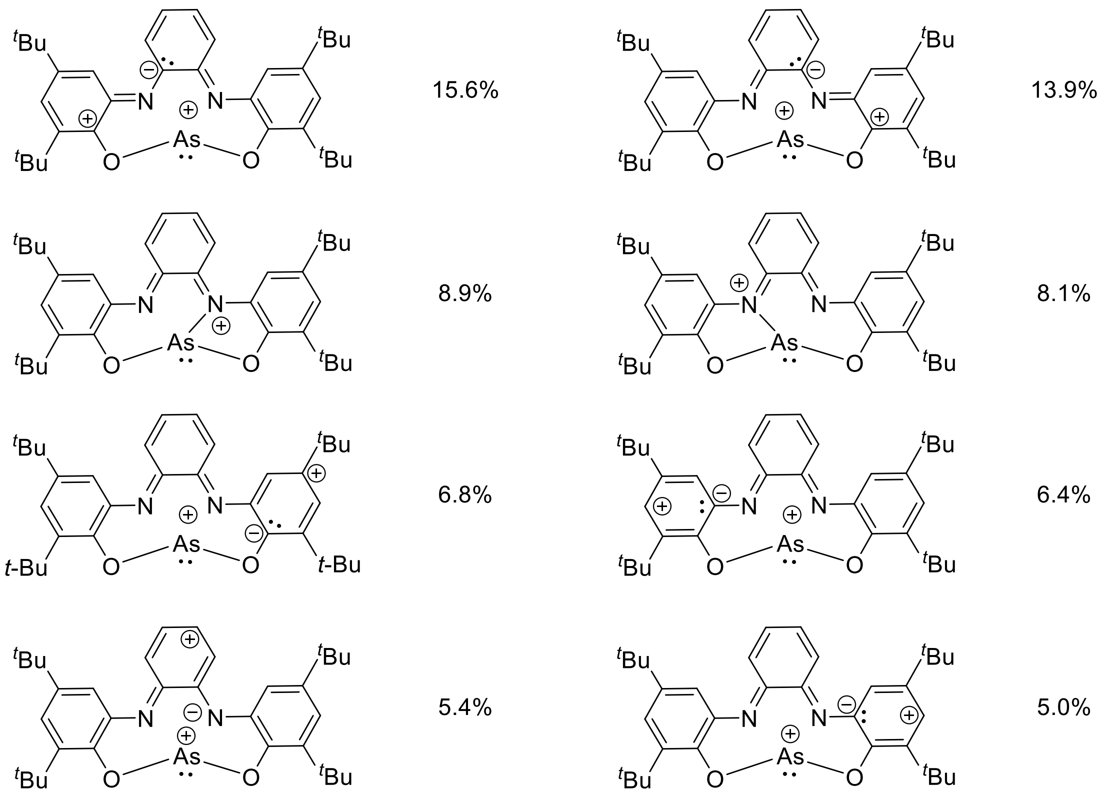

Figure S24 Selected resonance structures of **5** as obtained by NRT calculations.

Hydride and Fluoride Ion Affinities

Hydride and fluoride ion affinities were calculated isodesmically using the method described by Erdmann et al.[17] using the trimethylsilyl (TMS) reference system in combination with the B3LYP-D3(BJ)/def2-TZVP method for optimization and frequency calculations. Final single point energies were calculated at the PW6B95-D3(BJ)/def2-QZVPP level of theory.[18]

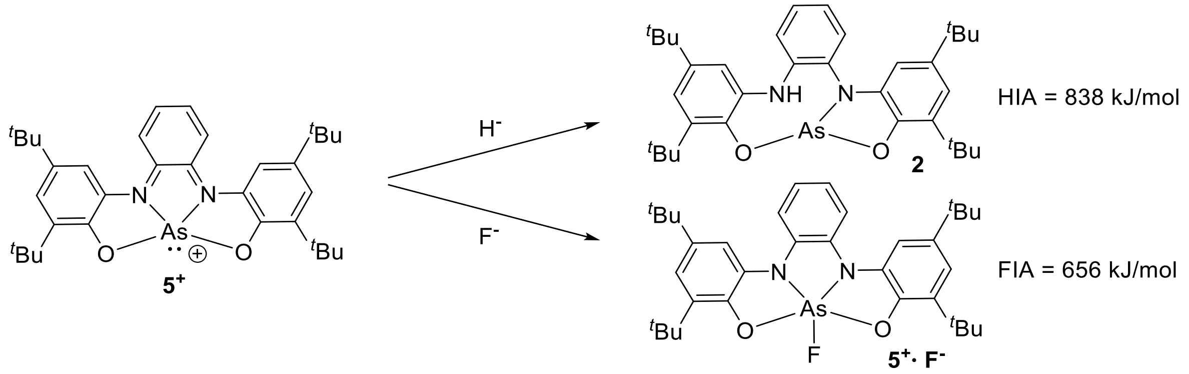

Energetic Landscape for the Dimerization of Radical **9**

Geometry optimization and frequency calculations were performed using the (U)PBE functional[19] in combination with the def2-SVP basis set and D3(BJ) dispersion correction. Single point energies of the optimized structures were obtained at the (U)B3LYP-D3(BJ)/def2-TZVP level including implicit solvation using the C-PCM model (acetonitrile). Relative free energies were determined at standard molarity (1 M) and temperature (298 K). For computational efficiency, truncated geometries were used in which the *tert*-butyl groups of the bisamidophenolate moiety were replaced by methyl groups as indicated by the "Me" superscripts.

Table S2 Overview of single point energies *SPE* ((U)B3LYP-D3(BJ)(C-PCM:CH_3CN)/def2-TZVP), correction terms for Gibbs free energy G_{corr} ((U)PBE-D3(BJ)/def2-SVP) and imaginary frequencies $\tilde{\nu}_i$ for transition states ((U)PBE-D3(BJ)/def2-SVP).

	SPE / E_h	G_{corr} / E_h	$\tilde{\nu}_i$ / cm^{-1}
**9^{Me}**	–3346.821141	0.301373	
**TS I**	–6693.661253	0.633610	*I*54.93
**Int A**	–6693.666221	0.633675	
**TS A**	–6693.657943	0.634317	*i*98.10
**Int I**	–6693.665193	0.633418	
**TS II**	–6693.654145	0.633614	*i*47.63
**Int II**	–6693.681583	0.633684	
**TS III**	–6693.668054	0.635496	*i*73.06
**10^{Me}**	–6693.689149	0.635989	
**TEMPO**	–483.957959	0.217373	
**Adduct I**	–3830.800849	0.546479	
**Adduct II**	–3830.763018	0.544159	

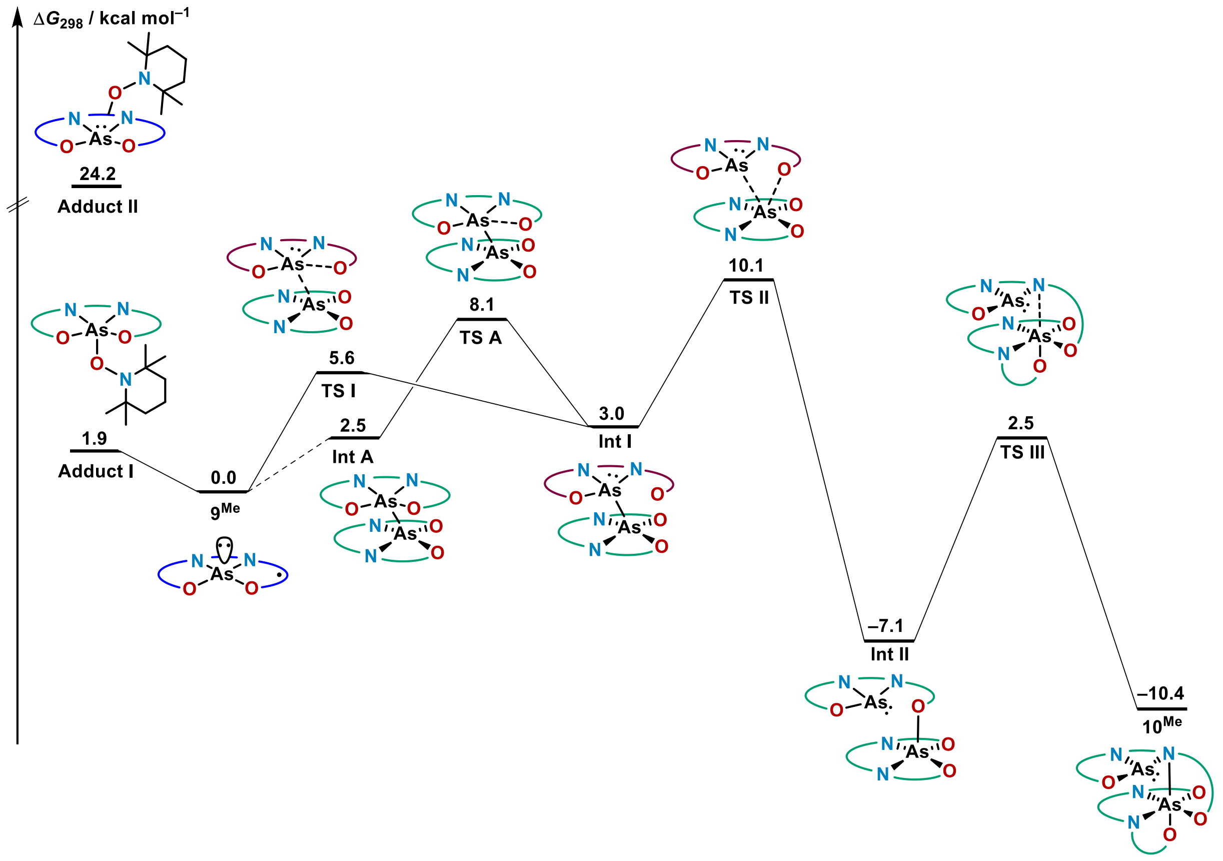

Figure S25 Energetic Landscape for the dimerization of **9** including scavenging of **9** by TEMPO at the (U)B3LYP-D3(BJ)(C-PCM:CH_3CN)/def2-TZVP//(U)PBE-D3(BJ)/def2-SVP level of theory.

Cartesian Coordinates of Optimized Geometries

2

As	-0.31528800	0.13458200	-2.57233300
O	0.46839600	-1.27924200	-1.68250600
O	-2.00988700	-0.37181400	-2.07947400
N	1.99880200	0.95803800	-2.10475300
H	2.65639600	0.99089300	-2.87419400
N	-0.42936100	1.32955500	-1.11981600
C	1.56684600	-1.11002100	-0.90483900
C	1.87736300	-2.04671900	0.10570700
C	3.03546400	-1.81955000	0.83966600
H	3.29028500	-2.52784500	1.61156300
C	3.88320200	-0.72041400	0.65804100
C	3.53044300	0.19398100	-0.32030500
H	4.11738300	1.08349800	-0.49485500
C	2.39388800	-0.00538800	-1.09437200
C	5.12286400	-0.56113900	1.54049800
C	4.69084800	-0.46310000	3.01536200
H	4.03572600	0.39610800	3.16897700
H	5.56463000	-0.34822600	3.66078400
H	4.15269700	-1.35526500	3.33620500
C	5.92094200	0.69966900	1.19027000
H	5.32750800	1.60486500	1.33052100
H	6.27624700	0.67772200	0.15829400
H	6.79470800	0.77273600	1.83968600
C	6.04361200	-1.78150200	1.35871200
H	6.93214800	-1.68435300	1.98663400
H	6.36660800	-1.87058200	0.31993900
H	5.54011000	-2.70883500	1.63269800
C	0.94882500	-3.22668300	0.41513300
C	1.50260100	-4.11418300	1.53773300
H	2.46982600	-4.54817400	1.27694400
H	0.80809500	-4.93678500	1.71380900
H	1.60942200	-3.56820500	2.47664600
C	-0.41430500	-2.67983000	0.88131600
H	-0.29581700	-2.05856100	1.77093500
H	-1.08072700	-3.50804100	1.13158800
H	-0.89234100	-2.08173500	0.11304400
C	0.76684600	-4.11609600	-0.82955300
H	0.32472700	-3.56581400	-1.65502000
H	0.11389300	-4.95776800	-0.58823400
H	1.72843600	-4.51794100	-1.15630200
C	1.70361000	2.26662500	-1.59966100
C	2.58943100	3.32850100	-1.65629300
H	3.56736300	3.18330600	-2.09910000
C	2.22843000	4.56727300	-1.13308000
H	2.92183400	5.39599900	-1.17457900
C	0.97555600	4.72417300	-0.55489800
H	0.68548700	5.68340500	-0.14627700
C	0.07208100	3.66843900	-0.51342600
H	-0.91698500	3.81410500	-0.10517900
C	0.42357800	2.42496800	-1.03982200
C	-1.49756900	1.01316200	-0.27211200
C	-2.35643400	0.04795200	-0.82097000
C	-3.45835800	-0.43070300	-0.11811000

C	-3.64237900	0.08203100	1.17366500
H	-4.48657700	-0.27016600	1.73959500
C	-2.78553900	1.01009300	1.76050700
C	-1.70146400	1.47972900	1.01634800
H	-1.00135900	2.18038700	1.44471800
C	-2.97364600	1.51415200	3.19507800
C	-3.09077400	3.04927400	3.19400900
H	-2.19733100	3.52102400	2.78389800
H	-3.22691400	3.42163700	4.21199000
H	-3.94481000	3.37024700	2.59491700
C	-1.75515700	1.09704300	4.04007600
H	-1.65212500	0.01060200	4.05503900
H	-1.86663800	1.44565400	5.06958200
H	-0.82970400	1.51401100	3.64196200
C	-4.23217100	0.94126300	3.85637500
H	-5.13567800	1.21598100	3.30945000
H	-4.32363600	1.33531400	4.87003000
H	-4.19185500	-0.14680000	3.92664900
C	-4.41785400	-1.45241700	-0.73734300
C	-3.66350300	-2.75480500	-1.06508600
H	-2.83539400	-2.57692900	-1.74680800
H	-4.34545900	-3.47082000	-1.52984200
H	-3.26662600	-3.20654600	-0.15518300
C	-5.02734400	-0.86549000	-2.02525900
H	-5.57839100	0.05086000	-1.80418600
H	-5.72337500	-1.58154300	-2.46857500
H	-4.25844700	-0.63495700	-2.75918800
C	-5.57153300	-1.80555700	0.20944500
H	-5.21353300	-2.25671600	1.13644300
H	-6.22629900	-2.52900600	-0.27911300
H	-6.17357400	-0.93101300	0.46201900

3 (anionic part)

O	0.97102300	1.37535100	-0.96243100
O	-1.57463200	1.21695400	-1.59213400
N	1.50280400	-1.08323900	-1.44710600
N	-1.03997600	-1.21453300	-1.19436200
C	2.12687000	0.92707300	-0.42728300
C	2.45008200	-0.43061200	-0.66964400
C	3.60167500	-0.96546800	-0.10797700
H	3.83749400	-2.00281400	-0.28428300
C	4.45044500	-0.18040400	0.68043900
C	4.10976700	1.14903700	0.89468100
H	4.75977700	1.76414500	1.49289600
C	2.94523900	1.73158100	0.36672100
C	2.55560900	3.18279800	0.67265900
C	1.21218500	3.20194700	1.42764000
H	0.43027600	2.71998600	0.84720600
H	0.90678100	4.23217000	1.63290300
H	1.30700800	2.67741600	2.38113300
C	3.59600600	3.89182700	1.54947200
H	3.71614800	3.39906600	2.51613200
H	3.26815000	4.91684900	1.73703400
H	4.57342100	3.93715800	1.06438100
C	2.42562800	3.98416300	-0.63687100

H	3.37839400	3.99449700	-1.17187600
H	2.14560800	5.01894800	-0.41852700
H	1.66994700	3.54954400	-1.28612700
C	5.71327300	-0.81611800	1.27214100
C	6.60991800	-1.34610100	0.13766700
H	7.51381100	-1.80986400	0.54407600
H	6.90823800	-0.53164500	-0.52529600
H	6.08988700	-2.09054800	-0.46527900
C	6.53803500	0.17624600	2.10015000
H	7.42382200	-0.32414800	2.49896000
H	5.96619200	0.56722700	2.94335500
H	6.87351800	1.02119400	1.49622100
C	5.31755800	-1.98736400	2.19080700
H	4.75274000	-2.74568400	1.64869900
H	4.69073900	-1.63308600	3.01116600
H	6.20671500	-2.46317800	2.61546200
C	1.02616700	-2.36257700	-1.19646600
C	-0.39249200	-2.43578300	-1.06190600
C	-0.99718100	-3.67535300	-0.86895500
H	-2.07296700	-3.74816000	-0.82124700
C	-0.22274000	-4.83251400	-0.77014700
H	-0.71139800	-5.78798600	-0.62333800
C	1.15663700	-4.76113100	-0.87833500
H	1.75837800	-5.65927900	-0.81185500
C	1.77662900	-3.53014700	-1.10176100
H	2.84745800	-3.48633400	-1.23518800
C	-2.41988700	0.60783300	-0.75853400
C	-3.48491400	1.25424600	-0.11500800
C	-4.28686400	0.48841400	0.73739100
H	-5.12386400	0.96606300	1.22357100
C	-4.05024900	-0.86042800	0.99659900
C	-2.97446100	-1.48012300	0.35971800
H	-2.74033200	-2.50855700	0.56778700
C	-2.17021900	-0.76493900	-0.52396900
C	-4.96480000	-1.61547400	1.96854700
C	-4.91045900	-0.94956600	3.35617500
H	-3.89137500	-0.96545800	3.74658700
H	-5.56001900	-1.47432600	4.06354800
H	-5.23125800	0.09132100	3.30927100
C	-4.55678800	-3.08436500	2.13392000
H	-4.60282100	-3.62233900	1.18542700
H	-5.23552100	-3.57891700	2.83308300
H	-3.54352400	-3.17835600	2.52729700
C	-6.41483100	-1.58252900	1.45056200
H	-6.77753700	-0.55932100	1.35010800
H	-7.08282700	-2.11434100	2.13521800
H	-6.47974100	-2.05584200	0.46897700
C	-3.73603800	2.74759900	-0.34896200
C	-4.01027100	3.00182800	-1.84366100
H	-3.17049700	2.67201600	-2.45086500
H	-4.17632900	4.06876200	-2.02165300
H	-4.90353200	2.45974700	-2.16360600
C	-2.49862100	3.55247400	0.09283000
H	-2.30782100	3.40298300	1.15764500
H	-2.66155400	4.62106400	-0.07790600
H	-1.61580700	3.24002500	-0.45888100

C	-4.94235600	3.26654300	0.44362600
H	-5.86571900	2.75790100	0.15832100
H	-5.07299400	4.33252300	0.24308300
H	-4.80262700	3.14462100	1.51957100
As	0.02191900	0.15500700	-2.07000000

4

As	0.00000000	-0.11421500	0.45457300
O	1.25106300	-1.30171300	-0.04314800
O	-1.25106300	-1.30171300	-0.04314800
N	-1.25475800	1.19880900	0.12225900
N	1.25475800	1.19880900	0.12225900
C	2.53706300	0.66510300	-0.01922700
C	-2.53706300	0.66510300	-0.01922700
C	2.50223800	-0.73587500	-0.10308800
C	-3.75690000	1.33168500	-0.05157300
H	-3.78976000	2.39910200	0.05474300
C	3.65987500	-1.49931500	-0.23917800
C	-2.50223800	-0.73587500	-0.10308700
C	0.71227700	2.46924700	-0.03399100
C	-0.71227600	2.46924700	-0.03399100
C	-6.30682900	1.28767100	-0.24937800
C	4.93535000	0.60599700	-0.19818100
C	-3.65987500	-1.49931500	-0.23917700
C	3.75690000	1.33168500	-0.05157300
H	3.78976000	2.39910200	0.05474200
C	2.75995500	-3.45715800	-1.53416400
H	2.73599500	-4.54699400	-1.60151800
H	3.19356300	-3.07039100	-2.45851800
H	1.73581800	-3.09915000	-1.45902600
C	-4.93535000	0.60599700	-0.19818100
C	-3.60530500	-3.02767900	-0.31941700
C	3.60530400	-3.02767900	-0.31941800
C	4.85770900	-0.78519700	-0.29108400
H	5.77507300	-1.34051200	-0.39899500
C	-6.99403300	0.95173800	-1.58574600
H	-6.39489200	1.30373500	-2.42733500
H	-7.97433100	1.43060400	-1.63898000
H	-7.13904100	-0.12204700	-1.70451800
C	-4.85770900	-0.78519700	-0.29108400
H	-5.77507300	-1.34051100	-0.39899600
C	6.30682900	1.28767100	-0.24937700
C	-6.20427400	2.81315600	-0.13640500
H	-5.75335300	3.11989900	0.80911300
H	-7.20277200	3.25048900	-0.18201300
H	-5.61943900	3.24024700	-0.95320300
C	-1.39859300	3.66432300	-0.21102300
H	-2.47428300	3.68274200	-0.22534500
C	7.17571100	0.77760400	0.91496500
H	6.70730400	1.00402400	1.87431800
H	8.15829600	1.25420300	0.89222600
H	7.32519900	-0.30085000	0.86230700
C	-7.17571100	0.77760400	0.91496400
H	-7.32519900	-0.30085000	0.86230600
H	-8.15829600	1.25420400	0.89222500

H	-6.70730400	1.00402400	1.87431800
C	-2.98812200	-3.59022600	0.97598500
H	-1.97263300	-3.23225300	1.12698500
H	-2.96284900	-4.68121000	0.93065000
H	-3.58611400	-3.30055000	1.84204000
C	1.39859300	3.66432300	-0.21102300
H	2.47428300	3.68274200	-0.22534600
C	6.99403300	0.95173800	-1.58574500
H	7.13904200	-0.12204800	-1.70451700
H	7.97433100	1.43060400	-1.63897900
H	6.39489300	1.30373500	-2.42733400
C	-2.75997800	-3.45715800	-1.53417800
H	-3.19360100	-3.07038800	-2.45852400
H	-2.73602200	-4.54699400	-1.60153500
H	-1.73583900	-3.09915200	-1.45905800
C	2.98814600	-3.59022700	0.97599500
H	1.97265800	-3.23225800	1.12701200
H	3.58615100	-3.30054700	1.84204000
H	2.96287700	-4.68121200	0.93066200
C	-0.69293700	4.85089600	-0.38766400
H	-1.23865100	5.77484300	-0.52298600
C	0.69293800	4.85089600	-0.38766400
H	1.23865100	5.77484300	-0.52298700
C	5.00226900	-3.63998600	-0.47979000
H	4.91162900	-4.72570300	-0.53448300
H	5.64980500	-3.40476600	0.36654900
H	5.49232300	-3.30230700	-1.39465300
C	-5.00227200	-3.63998700	-0.47976200
H	-5.64979200	-3.40476900	0.36659100
H	-4.91163200	-4.72570400	-0.53445900
H	-5.49234400	-3.30230700	-1.39461500
C	6.20427500	2.81315600	-0.13640400
H	5.61944000	3.24024700	-0.95320200
H	7.20277200	3.25048900	-0.18201300
H	5.75335300	3.11989800	0.80911400
Cl	0.00000000	-0.41393600	2.63825700

5 (cationic part)

O	1.25841300	1.34471500	-0.31969700
O	-1.26293200	1.32341000	-0.29460400
N	1.27097700	-1.16874200	-0.38038600
N	-1.24890600	-1.18891700	-0.36642400
C	2.46235800	0.78842100	-0.12527500
C	2.50983700	-0.63845200	-0.13665800
C	3.71369600	-1.30268900	0.11518200
H	3.74811400	-2.37686300	0.14195500
C	4.86194100	-0.57428900	0.36402600
C	4.78147600	0.83247000	0.33216700
H	5.68161400	1.38990200	0.52198900
C	3.61902600	1.55243000	0.08276600
C	3.58642800	3.08327100	0.06267600
C	2.63908200	3.59889800	1.16431200
H	1.61633900	3.26129800	1.01283800
H	2.63935500	4.68999300	1.16401500
H	2.97243300	3.26338200	2.14795900

C	4.97584300	3.68050100	0.31695700
H	5.36593000	3.40761500	1.29891100
H	4.90595300	4.76762100	0.28413200
H	5.69835400	3.37544100	-0.44200900
C	3.10694400	3.57047500	-1.31885400
H	3.77210600	3.21383700	-2.10731300
H	3.11172700	4.66115500	-1.34388400
H	2.09611100	3.23515300	-1.53945100
C	6.17196300	-1.30746400	0.66587300
C	6.52248100	-2.22906800	-0.51766200
H	7.45770300	-2.75385600	-0.31767500
H	6.64461200	-1.65478200	-1.43727100
H	5.75197600	-2.98160700	-0.68981500
C	7.34346800	-0.34389100	0.88801900
H	8.24751900	-0.91747900	1.09241000
H	7.17438100	0.31447800	1.74157600
H	7.53814400	0.27135900	0.00802600
C	5.99107700	-2.15339700	1.94072900
H	5.20400100	-2.89948200	1.82438700
H	5.73471700	-1.52352300	2.79393800
H	6.91722900	-2.68092200	2.17348800
C	0.74999300	-2.39959800	-0.40346200
C	-0.71059600	-2.41226700	-0.39520500
C	-1.38639800	-3.65678500	-0.40881500
H	-2.46100700	-3.68967400	-0.45201700
C	-0.66970000	-4.81924600	-0.40070100
H	-1.18940200	-5.76743100	-0.41296300
C	0.74812100	-4.80672500	-0.41227200
H	1.28370600	-5.74593300	-0.43475000
C	1.44563800	-3.63248400	-0.42929900
H	2.51967400	-3.64671700	-0.49160300
C	-2.45535300	0.75379800	-0.10815800
C	-3.62624200	1.50789600	0.09408800
C	-4.77538400	0.77439200	0.33104900
H	-5.68777000	1.31615000	0.51872700
C	-4.84307000	-0.64034700	0.35525600
C	-3.69250000	-1.35595200	0.11275800
H	-3.71275700	-2.42888000	0.13031500
C	-2.48889000	-0.67373400	-0.12543400
C	-6.18369300	-1.31044800	0.64727400
C	-6.67006300	-0.87446100	2.04313100
H	-5.95846000	-1.17204500	2.81493300
H	-7.62952700	-1.34381500	2.26508800
H	-6.80711200	0.20495600	2.10835400
C	-6.08393900	-2.83917200	0.62395500
H	-5.77011900	-3.21040500	-0.35380900
H	-7.06190000	-3.27034000	0.83701100
H	-5.39113200	-3.21272000	1.38061200
C	-7.20906100	-0.87273000	-0.41668200
H	-7.36372100	0.20622200	-0.41633300
H	-8.17261100	-1.34415100	-0.21810900
H	-6.88494600	-1.16759500	-1.41597600
C	-3.60846000	3.03866300	0.07850100
C	-3.12700500	3.53418500	-1.29939800
H	-2.11103600	3.21149100	-1.51499400
H	-3.14427400	4.62473300	-1.32173100

H	-3.78363200	3.17196100	-2.09246000
C	-2.67229500	3.56074300	1.18657300
H	-3.00651600	3.21826100	2.16752000
H	-2.68509500	4.65169000	1.18980900
H	-1.64495000	3.23579000	1.03890600
C	-5.00573600	3.61940300	0.32718000
H	-5.72046800	3.30916500	-0.43714900
H	-4.94808700	4.70728800	0.29856800
H	-5.39777600	3.33887400	1.30629200
As	0.00625300	0.22984600	-1.09357500

5 · 4-DMAP (cationic part)

As	-0.00000300	0.23366800	-0.34266500
O	1.27949900	-0.52863800	-1.33363300
O	-1.27950900	-0.52865100	-1.33361700
N	0.00001200	-1.14955300	1.03921400
N	-1.25900400	1.38008500	0.34130200
N	1.25899600	1.38009100	0.34129500
N	0.00002900	-4.05702300	3.97736100
C	2.55088900	1.02399200	-0.08385700
C	-0.00000800	-2.45858500	0.70104700
H	-0.00002900	-2.67868800	-0.35530200
C	-2.55089800	1.02398300	-0.08384600
C	2.53201700	-0.03255300	-1.00625300
C	-3.76043400	1.56600500	0.33403200
H	-3.77369300	2.33774400	1.08111100
C	3.70108400	-0.54041200	-1.57247400
C	-2.53202600	-0.03256600	-1.00623800
C	0.71113500	2.56711900	0.83906800
C	-0.71114500	2.56711600	0.83907200
C	-0.00000300	-3.44436000	1.64194700
H	-0.00002100	-4.46847200	1.30761200
C	-6.31467400	1.63374600	0.22533600
C	4.95060100	1.07869200	-0.19518200
C	-3.70109400	-0.54042600	-1.57245600
C	3.76042500	1.56601200	0.33402300
H	3.77368400	2.33774800	1.08110500
C	2.87190100	-1.22411600	-3.84232800
H	2.86492900	-2.02171100	-4.58749100
H	3.32833000	-0.34327400	-4.29653400
H	1.84034200	-0.98531900	-3.59243800
C	0.00004100	-1.72767900	3.34052700
H	0.00006100	-1.38332700	4.36131500
C	-4.95061000	1.07868300	-0.19517000
C	0.00003600	-0.79785300	2.34387300
H	0.00005100	0.26169500	2.55578300
C	-3.67483900	-1.67166000	-2.60547000
C	3.67482900	-1.67164500	-2.60548900
C	0.00002300	-3.11513700	3.02557800
C	4.88833100	0.04661500	-1.13564200
H	5.81457100	-0.32424600	-1.54219000
C	0.00004400	-3.68367500	5.38964000
H	0.88891000	-3.10252200	5.64292300
H	0.00003300	-4.58542900	5.99236500
H	-0.88879900	-3.10249300	5.64293300

C	-7.04535700	2.17974600	-1.01546100
H	-6.47001200	2.97958300	-1.48447500
H	-8.01939400	2.58172700	-0.73114400
H	-7.21137100	1.40326900	-1.76212000
C	-4.88834000	0.04660300	-1.13562700
H	-5.81458000	-0.32426000	-1.54217300
C	6.31466600	1.63375300	0.22532600
C	-6.19002700	2.77031100	1.24621300
H	-5.70917900	2.43861000	2.16861200
H	-7.18353000	3.13458600	1.50881200
H	-5.62728700	3.61519000	0.84452100
C	0.00001700	-5.47310800	3.61801100
H	-0.88879000	-5.73193000	3.03928300
H	0.00004900	-6.06680100	4.52574000
H	0.88878600	-5.73192800	3.03922300
C	-1.40092400	3.70139200	1.25071800
H	-2.47658400	3.73041100	1.22377700
C	7.14961500	0.50482600	0.85739500
H	6.65134600	0.10133100	1.74085300
H	8.12726200	0.88354000	1.16078600
H	7.31224100	-0.31587500	0.15869200
C	-7.14962600	0.50482300	0.85740700
H	-7.31225400	-0.31588000	0.15870700
H	-8.12727100	0.88354000	1.16079900
H	-6.65135700	0.10132900	1.74086600
C	-3.03184900	-2.92906000	-1.98843900
H	-1.99380900	-2.75234600	-1.71565600
H	-3.05608000	-3.74880000	-2.70852600
H	-3.57806000	-3.24455900	-1.09730700
C	1.40091200	3.70139800	1.25070800
H	2.47657200	3.73042100	1.22375900
C	7.04535000	2.17975600	-1.01546800
H	7.21136800	1.40328000	-1.76212700
H	8.01938500	2.58174000	-0.73114800
H	6.47000300	2.97959200	-1.48448200
C	-2.87192200	-1.22412800	-3.84231500
H	-3.32836100	-0.34329100	-4.29652000
H	-2.86494700	-2.02172400	-4.58747600
H	-1.84036400	-0.98532100	-3.59243000
C	3.03185100	-2.92904800	-1.98845400
H	1.99382200	-2.75233100	-1.71563100
H	3.57809200	-3.24456500	-1.09734700
H	3.05604900	-3.74877700	-2.70855500
C	-0.69401000	4.81619700	1.68570800
H	-1.23754600	5.69005800	2.01715500
C	0.69399700	4.81620000	1.68570300
H	1.23753100	5.69006400	2.01714600
C	5.08628300	-2.04998100	-3.07109300
H	5.01629000	-2.85056100	-3.80796400
H	5.70541900	-2.41152400	-2.24851100
H	5.59616600	-1.21069200	-3.54571200
C	-5.08629500	-2.05000500	-3.07106300
H	-5.70542200	-2.41155200	-2.24847700
H	-5.01630200	-2.85058400	-3.80793600
H	-5.59618600	-1.21071800	-3.54567800
C	6.19001900	2.77031400	1.24620700

H	5.62727900	3.61519500	0.84451700
H	7.18352200	3.13458800	1.50880800
H	5.70917000	2.43861100	2.16860500

5 · py (cationic part)

As	0.00000000	-0.08148800	-0.09702100
O	1.28759600	-1.26710800	-0.44560100
O	-1.28759600	-1.26710800	-0.44560100
N	0.00000000	-0.49669500	1.88129800
N	-1.25859700	1.24779000	-0.09831100
N	1.25859800	1.24779000	-0.09831100
C	2.55454800	0.71887000	-0.22866200
C	0.00000100	-1.77366500	2.29213500
H	0.00000100	-2.52779600	1.52030000
C	-2.55454700	0.71887000	-0.22866200
C	2.54043700	-0.67092800	-0.42020500
C	-3.76250000	1.40279600	-0.15512200
H	-3.77279200	2.45829900	0.04325500
C	3.71291400	-1.40644900	-0.59512300
C	-2.54043700	-0.67092800	-0.42020600
C	0.71161600	2.52110500	-0.28282900
C	-0.71161600	2.52110500	-0.28282900
C	0.00000100	-2.07820200	3.63810300
H	0.00000200	-3.11254000	3.94945300
C	-6.31800800	1.39932600	-0.23284000
C	4.95525000	0.70475500	-0.30559800
C	-3.71291300	-1.40644900	-0.59512400
C	3.76250000	1.40279700	-0.15512400
H	3.77279300	2.45830000	0.04325300
C	2.92109900	-3.21876800	-2.14169200
H	2.91881300	-4.29374300	-2.33026200
H	3.39796800	-2.72911200	-2.99210400
H	1.88745200	-2.88319000	-2.08820500
C	0.00000000	0.27529100	4.11584500
H	0.00000000	1.10567400	4.80667800
C	-4.95524900	0.70475500	-0.30559600
C	0.00000000	0.51868500	2.75775600
H	0.00000000	1.51666800	2.34335700
C	-3.69280800	-2.91841200	-0.84207700
C	3.69280800	-2.91841200	-0.84207400
C	0.00000100	-1.04114200	4.56322300
C	4.89744700	-0.67468700	-0.52762100
H	5.82654400	-1.20691200	-0.64601000
C	-7.06845400	1.18182500	-1.56003000
H	-6.50591200	1.59732900	-2.39767600
H	-8.04176000	1.67410800	-1.52530800
H	-7.23822500	0.12407900	-1.76081400
C	-4.89744700	-0.67468700	-0.52762100
H	-5.82654400	-1.20691200	-0.64601100
C	6.31800900	1.39932600	-0.23284200
C	-6.18897300	2.90819000	0.00386200
H	-5.69098400	3.13108100	0.94961300
H	-7.18191500	3.35627500	0.04475200
H	-5.64187700	3.39968600	-0.80305500
C	-1.40190100	3.70524900	-0.51809300

H	-2.47742100	3.71648800	-0.55724100
C	7.13497300	0.79565200	0.92450600
H	6.62206600	0.93641600	1.87768300
H	8.11072100	1.28014200	0.98843400
H	7.30248800	-0.27259600	0.78679700
C	-7.13497300	0.79565100	0.92450700
H	-7.30248800	-0.27259700	0.78679600
H	-8.11072100	1.28014000	0.98843400
H	-6.62206700	0.93641200	1.87768400
C	-3.02176400	-3.63545800	0.34572000
H	-1.97768000	-3.34711400	0.44699200
H	-3.05783000	-4.71543800	0.19428300
H	-3.54040000	-3.40740600	1.27892600
C	1.40190000	3.70525000	-0.51809300
H	2.47742000	3.71649000	-0.55724200
C	7.06845500	1.18182300	-1.56003100
H	7.23822700	0.12407600	-1.76081400
H	8.04176100	1.67410600	-1.52530900
H	6.50591400	1.59732500	-2.39767800
C	-2.92110200	-3.21876900	-2.14169600
H	-3.39797200	-2.72911100	-2.99210700
H	-2.91882100	-4.29374500	-2.33026700
H	-1.88745400	-2.88319600	-2.08821100
C	3.02176500	-3.63545900	0.34572400
H	1.97768100	-3.34711600	0.44699700
H	3.54040200	-3.40740600	1.27892800
H	3.05783200	-4.71543900	0.19428600
C	-0.69454700	4.88321900	-0.71958600
H	-1.23729300	5.80311300	-0.88691000
C	0.69454600	4.88322000	-0.71958700
H	1.23729100	5.80311400	-0.88691100
C	5.10882200	-3.48853800	-0.99114900
H	5.04379900	-4.56166600	-1.17196200
H	5.70640200	-3.34141400	-0.09013100
H	5.63870800	-3.04541400	-1.83521100
C	-5.10882400	-3.48853600	-0.99115000
H	-5.70640000	-3.34142000	-0.09012900
H	-5.04380200	-4.56166300	-1.17197100
H	-5.63871200	-3.04540500	-1.83520600
C	6.18897400	2.90819000	0.00385700
H	5.64187900	3.39968500	-0.80306100
H	7.18191600	3.35627500	0.04474700
H	5.69098300	3.13108300	0.94960700
H	0.00000100	-1.25615300	5.62328500

$5^{+} \cdot F^{-}$

As	0.00000000	-0.12885700	0.61756800
O	1.26038700	-1.32484700	0.18659000
O	-1.26038700	-1.32484700	0.18659100
N	-1.26258300	1.19016100	0.36376800
N	1.26258300	1.19016100	0.36376700
C	2.53858200	0.65057000	0.15795200
C	-2.53858200	0.65057000	0.15795200
C	2.50342900	-0.74892100	0.06301600
C	-3.75399800	1.32077800	0.07859000

H	-3.78925400	2.38572400	0.20826000
C	3.65388200	-1.50524900	-0.15413500
C	-2.50342900	-0.74892100	0.06301700
C	0.71178600	2.44213200	0.09374300
C	-0.71178600	2.44213200	0.09374300
C	-6.29357600	1.28756900	-0.22798300
C	4.92665700	0.60163000	-0.13162600
C	-3.65388200	-1.50524900	-0.15413400
C	3.75399800	1.32077700	0.07859000
H	3.78925400	2.38572400	0.20826100
C	2.69117500	-3.43079600	-1.45084700
H	2.66574400	-4.51844600	-1.54659500
H	3.07519900	-3.01773700	-2.38569500
H	1.67168900	-3.07861400	-1.31231100
C	-4.92665700	0.60163000	-0.13162600
C	-3.59841400	-3.03155800	-0.27071100
C	3.59841400	-3.03155800	-0.27071200
C	4.84667500	-0.78735600	-0.24911300
H	5.75874600	-1.33815600	-0.41124400
C	-6.92445900	0.97803000	-1.59806000
H	-6.29034100	1.34483200	-2.40706800
H	-7.90125700	1.45943100	-1.68317000
H	-7.06536900	-0.09306800	-1.74308000
C	-4.84667500	-0.78735600	-0.24911200
H	-5.75874600	-1.33815600	-0.41124300
C	6.29357600	1.28756900	-0.22798200
C	-6.19308600	2.81040900	-0.08267200
H	-5.78187000	3.09871000	0.88642900
H	-7.18790900	3.25122100	-0.16233800
H	-5.57347700	3.25075600	-0.86614400
C	-1.39742900	3.61479300	-0.19815400
H	-2.47296100	3.63063500	-0.21824700
C	7.21173200	0.75817700	0.88893600
H	6.78391100	0.96548400	1.87128800
H	8.19173900	1.23743600	0.83375500
H	7.36043800	-0.31878400	0.80974300
C	-7.21173200	0.75817800	0.88893600
H	-7.36043800	-0.31878300	0.80974300
H	-8.19173900	1.23743700	0.83375500
H	-6.78391100	0.96548600	1.87128800
C	-3.05192400	-3.63192900	1.03917900
H	-2.04551400	-3.28074800	1.25412100
H	-3.02527200	-4.72125600	0.96437700
H	-3.69528300	-3.36565700	1.87992100
C	1.39742900	3.61479200	-0.19815400
H	2.47296100	3.63063500	-0.21824900
C	6.92446000	0.97803100	-1.59806000
H	7.06537000	-0.09306700	-1.74308100
H	7.90125700	1.45943200	-1.68317000
H	6.29034200	1.34483300	-2.40706700
C	-2.69117800	-3.43079500	-1.45084900
H	-3.07520500	-3.01773600	-2.38569600
H	-2.66574700	-4.51844600	-1.54659800
H	-1.67169300	-3.07861400	-1.31231500
C	3.05192800	-3.63192900	1.03918000
H	2.04551700	-3.28074900	1.25412300

H	3.69528700	-3.36565300	1.87992100
H	3.02527700	-4.72125600	0.96437900
C	-0.69286900	4.78205800	-0.47635700
H	-1.23913500	5.69035100	-0.69177400
C	0.69286900	4.78205800	-0.47635700
H	1.23913500	5.69035100	-0.69177400
C	4.98609600	-3.63466900	-0.52156100
H	4.89453300	-4.71869100	-0.60232900
H	5.67682600	-3.42167000	0.29600800
H	5.42688600	-3.26966300	-1.45086700
C	-4.98609700	-3.63466900	-0.52155700
H	-5.67682500	-3.42166800	0.29601400
H	-4.89453400	-4.71869100	-0.60232200
H	-5.42688900	-3.26966500	-1.45086200
C	6.19308600	2.81040800	-0.08267000
H	5.57347700	3.25075700	-0.86614200
H	7.18790900	3.25122000	-0.16233600
H	5.78187000	3.09870900	0.88643100
F	0.00000000	-0.36273400	2.33039600

6 (cationic part)

As	0.00000600	-0.19026200	-0.03052700
O	1.33082200	-0.22053100	-1.24421800
O	-1.33081200	-0.22070200	-1.24421200
N	0.00013900	-2.25975600	-0.12853000
N	-0.00012600	1.90116900	-0.22956300
N	-1.26525300	-0.13600200	1.31425300
N	1.26526400	-0.13585000	1.31425000
N	0.00042300	-6.40494700	-0.39195700
N	-0.00039500	6.03312500	-0.65876400
C	2.55130000	-0.00481000	0.78417900
C	0.00016200	-2.87394900	-1.32574900
H	0.00010400	-2.22964600	-2.19040200
C	-2.55130100	-0.00505900	0.78418600
C	2.56110500	-0.06373900	-0.62395100
C	-3.74154100	0.19542000	1.47451200
H	-3.73208300	0.27461100	2.54475400
C	3.74323300	0.03975000	-1.35504500
C	-2.56110600	-0.06399900	-0.62394400
C	0.71660900	-0.13104100	2.59504700
C	-0.71659500	-0.13112700	2.59504800
C	-0.00016600	2.70436600	0.84840500
H	-0.00012500	2.22134600	1.81362000
C	0.00025400	-4.23505000	-1.45850600
H	0.00026700	-4.65328700	-2.45161000
C	-0.00017600	2.46683000	-1.44798000
H	-0.00014300	1.78847400	-2.28544300
C	-6.27770100	0.53012800	1.49396000
C	4.94106900	0.31164600	0.77790400
C	-3.74324400	0.03939600	-1.35503300
C	3.74153000	0.19574200	1.47450200
H	3.73207200	0.27491900	2.54474500
C	2.89240800	1.05233700	-3.50160400
H	2.96186400	1.01803000	-4.59054800
H	3.23295200	2.03517100	-3.17059400

H	1.84790800	0.93775000	-3.22511900
C	0.00030200	-4.38735500	0.94013500
H	0.00035300	-4.92757000	1.87247100
C	-4.94109100	0.31123400	0.77791800
C	0.00020800	-3.01919900	0.98178000
H	0.00018700	-2.49912400	1.92724000
C	-3.76094400	-0.06092800	-2.88523700
C	3.76093100	-0.06054300	-2.88525000
C	0.00033000	-5.06139300	-0.30656600
C	4.91284500	0.22282300	-0.61354400
H	5.84525400	0.30269500	-1.14705000
C	0.00049600	-7.21933500	0.81659900
H	0.88834200	-7.02803100	1.42377500
H	0.00057200	-8.26769200	0.53650100
H	-0.88736600	-7.02816400	1.42379300
C	-0.00030800	4.69325300	-0.51963200
C	-6.90113700	1.85427000	1.01530400
H	-6.24211600	2.69527900	1.23852400
H	-7.85559900	2.02610400	1.51662700
H	-7.08476300	1.84778200	-0.05913900
C	-4.91286700	0.22239400	-0.61353000
H	-5.84528300	0.30219300	-1.14703200
C	-0.00025400	4.07004300	0.75323600
H	-0.00028000	4.64763100	1.66293000
C	6.27766700	0.53062200	1.49394400
C	-6.11831700	0.59880900	3.01705500
H	-5.70732400	-0.32740400	3.42328000
H	-7.09357400	0.75507400	3.47931200
H	-5.47458100	1.42648700	3.32090600
C	-0.00026500	3.82191100	-1.63703400
H	-0.00030000	4.19933300	-2.64636100
C	0.00044500	-7.05982100	-1.69385500
H	-0.88746600	-6.79337800	-2.27192200
H	0.00052800	-8.13518200	-1.54861700
H	0.88829200	-6.79324600	-2.27195800
C	-1.39826600	-0.17128700	3.80463200
H	-2.47383900	-0.20447000	3.82525700
C	-0.00043700	6.89510600	0.51585800
H	-0.88819400	6.72856100	1.13055500
H	-0.00050600	7.93147000	0.19403300
H	0.88735600	6.72867300	1.13053500
C	7.23058200	-0.63267000	1.16265600
H	6.80878100	-1.58422600	1.49101000
H	8.18863000	-0.49186500	1.66681900
H	7.42414100	-0.70303100	0.09229200
C	-7.23053800	-0.63323300	1.16269100
H	-7.42409900	-0.70361900	0.09232900
H	-8.18859300	-0.49248700	1.66685800
H	-6.80867100	-1.58475600	1.49105400
C	-3.22455000	-1.43930200	-3.31723800
H	-2.20028100	-1.58943700	-2.98417300
H	-3.24653100	-1.52467100	-4.40557300
H	-3.84160800	-2.23788400	-2.90183100
C	1.39828400	-0.17111300	3.80463100
H	2.47386100	-0.20415800	3.82525800
C	6.90101200	1.85481200	1.01530400

H	7.08463500	1.84835100	-0.05914000
H	7.85546400	2.02670400	1.51662600
H	6.24193500	2.69577400	1.23853700
C	-2.89249000	1.05198700	-3.50162400
H	-3.23307500	2.03480900	-3.17061800
H	-2.96196800	1.01766300	-4.59056500
H	-1.84797800	0.93745400	-3.22516200
C	3.22461800	-1.43893800	-3.31728100
H	2.20035600	-1.58913800	-2.98422500
H	3.84171900	-2.23749400	-2.90188800
H	3.24660900	-1.52428400	-4.40561800
C	-0.00044900	6.63452800	-1.98578300
H	0.88729500	6.34480100	-2.55297700
H	-0.00051800	7.71496500	-1.88449400
H	-0.88816700	6.34468600	-2.55295700
C	-0.69180300	-0.19429400	5.00717900
H	-1.23846200	-0.22101800	5.93974200
C	0.69182400	-0.19420700	5.00717900
H	1.23848700	-0.22086100	5.93974100
C	5.17841500	0.08784900	-3.45257500
H	5.13868800	0.00486700	-4.53928200
H	5.84783100	-0.69193000	-3.08709000
H	5.61428200	1.05840300	-3.21102700
C	-5.17843900	0.08736700	-3.45255700
H	-5.84780800	-0.69244400	-3.08705200
H	-5.13871400	0.00436300	-4.53926300
H	-5.61436200	1.05790000	-3.21102800
C	6.11828500	0.59927200	3.01704100
H	5.47449500	1.42690300	3.32090500
H	7.09353400	0.75559500	3.47929600
H	5.70735500	-0.32697400	3.42325500

7 (cationic part)

As	0.00000000	0.38563000	-0.34102900
O	-1.28999500	1.00823100	0.76052500
O	1.28999400	1.00823000	0.76052700
N	-1.27935900	-0.80080200	-0.99358200
N	1.27936400	-0.80079600	-0.99358600
N	-1.08157700	2.52042500	-2.09215100
N	1.08157400	2.52043100	-2.09214400
C	-2.42719600	0.22299900	0.76051700
C	-2.44674800	-0.79897100	-0.20209000
C	-3.52119700	-1.66978900	-0.26326600
H	-3.52829500	-2.47526700	-0.97596100
C	-4.59250800	-1.51303800	0.62017600
C	-4.54101400	-0.48061100	1.55259600
H	-5.36851700	-0.35645200	2.22796200
C	-3.46199800	0.40549200	1.66979400
C	-3.41248600	1.50204200	2.73927100
C	-3.28375600	2.88476400	2.07108600
H	-2.35638300	2.97329400	1.50761900
H	-3.28738400	3.66723700	2.83182500
H	-4.12684800	3.06802100	1.40093100
C	-2.20863100	1.25721500	3.67010600
H	-2.29557500	0.28630200	4.16033100
H	-2.17719400	2.02466200	4.44576700

H	-1.26622500	1.28492000	3.12783000
C	-4.68135100	1.51616000	3.60044500
H	-5.57619100	1.71068700	3.00705300
H	-4.59944900	2.30895200	4.34456300
H	-4.81966200	0.57615600	4.13586600
C	-5.77427200	-2.48408000	0.53180900
C	-6.86317600	-2.17144800	1.56414600
H	-7.67845400	-2.88800300	1.45978600
H	-7.28190800	-1.17353700	1.42360200
H	-6.48739900	-2.24748000	2.58563300
C	-5.27163200	-3.91848000	0.78042700
H	-4.81837600	-4.00453200	1.76920300
H	-4.52769500	-4.22189600	0.04304600
H	-6.10265500	-4.62371700	0.72384800
C	-6.40224600	-2.39783300	-0.87160900
H	-7.24793400	-3.08327900	-0.94934900
H	-5.68811400	-2.66329100	-1.65214100
H	-6.76350900	-1.38792200	-1.07394200
C	-0.70892100	-1.83281200	-1.75444800
C	0.70892800	-1.83280900	-1.75445000
C	1.39787400	-2.76347200	-2.52199700
H	2.47396500	-2.74039700	-2.57310500
C	0.69350300	-3.70557800	-3.26426200
H	1.23755800	-4.42577100	-3.85953700
C	-0.69349300	-3.70558100	-3.26426000
H	-1.23754600	-4.42577800	-3.85953300
C	-1.39786600	-2.76348000	-2.52199200
H	-2.47395700	-2.74041000	-2.57309600
C	2.44675100	-0.79896500	-0.20209200
C	3.52120200	-1.66978200	-0.26327000
H	3.52830300	-2.47525600	-0.97596900
C	4.59251100	-1.51303200	0.62017600
C	4.54101200	-0.48061100	1.55260100
H	5.36851300	-0.35645300	2.22797000
C	3.46199400	0.40549000	1.66980000
C	2.42719600	0.22300000	0.76051900
C	3.41247700	1.50203400	2.73928400
C	2.20862100	1.25719700	3.67011400
H	2.17717900	2.02463800	4.44578000
H	2.29556700	0.28628100	4.16033200
H	1.26621600	1.28490200	3.12783600
C	4.68134000	1.51615100	3.60046000
H	4.59943300	2.30893800	4.34458400
H	5.57618100	1.71068700	3.00707100
H	4.81965400	0.57614400	4.13587400
C	3.28374400	2.88476100	2.07110700
H	2.35637200	2.97329100	1.50763900
H	4.12683600	3.06802500	1.40095500
H	3.28736900	3.66722800	2.83185200
C	5.77427700	-2.48407000	0.53180400
C	6.86317800	-2.17144400	1.56414800
H	7.28190900	-1.17353100	1.42361100
H	7.67845700	-2.88799700	1.45978600
H	6.48739700	-2.24748300	2.58563300
C	6.40225500	-2.39781100	-0.87161100
H	5.68812700	-2.66327000	-1.65214600

H	7.24794800	-3.08325000	-0.94935300
H	6.76351100	-1.38789600	-1.07393800
C	5.27164200	-3.91847400	0.78041000
H	4.81838300	-4.00453500	1.76918500
H	6.10266600	-4.62370800	0.72382800
H	4.52770700	-4.22188800	0.04302600
C	-0.00000100	1.88863300	-1.60267200
C	-2.48013400	2.17977400	-1.82183500
H	-2.60255500	1.10303300	-1.83872400
H	-3.09600500	2.61897500	-2.59852300
H	-2.77774600	2.56578600	-0.85040600
C	-1.64468000	4.48506500	-3.57211000
H	-2.24547800	3.95197900	-4.31180200
H	-1.10588300	5.27301100	-4.09237200
H	-2.32364700	4.95863700	-2.86117600
C	-0.68394800	3.58055000	-2.88996300
C	0.68394500	3.58055400	-2.88996000
C	1.64467600	4.48507600	-3.57209800
H	2.32363400	4.95865200	-2.86115900
H	1.10587700	5.27301800	-4.09236300
H	2.24548300	3.95199500	-4.31178600
C	2.48013200	2.17978600	-1.82182200
H	2.77773400	2.56578900	-0.85038500
H	3.09600600	2.61899900	-2.59849900
H	2.60256100	1.10304600	-1.83872100

8 (cationic part)

As	0.07453700	-0.26610800	0.12961700
O	-1.19956400	-0.69659800	1.33870900
O	1.35361400	-0.68339600	1.32998900
N	-1.19875300	-0.63131700	-1.18166600
N	1.35149500	-0.56247200	-1.19048900
N	-2.11761700	3.12903400	0.15679100
N	1.57094900	3.69164700	0.15090100
C	-2.35219000	-1.18805300	0.76359600
C	-2.38068300	-1.16441900	-0.64241400
C	-3.49172000	-1.64373000	-1.31508100
H	-3.51521900	-1.64897200	-2.39005300
C	-4.58825300	-2.12608800	-0.59530800
C	-4.51990100	-2.13727500	0.79466700
H	-5.36085200	-2.51416000	1.34896500
C	-3.40341600	-1.69506100	1.51797300
C	-3.32790800	-1.78241600	3.04642500
C	-4.60110000	-2.39108300	3.64681200
H	-4.49793700	-2.43668300	4.73141400
H	-4.77414800	-3.40697000	3.28940400
H	-5.48565300	-1.79160100	3.42501700
C	-2.13998400	-2.67966000	3.44720900
H	-2.09701900	-2.77287800	4.53396600
H	-1.19193900	-2.27196800	3.10432800
H	-2.25732900	-3.68006600	3.02746100
C	-3.14932400	-0.37718600	3.65171100
H	-4.00918800	0.25231000	3.41720100
H	-2.24828500	0.10940100	3.28358400
H	-3.07513000	-0.44915200	4.73832900

C	-5.81923000	-2.61798900	-1.36354100
C	-6.92303400	-3.12806600	-0.43064600
H	-7.77343000	-3.46568900	-1.02398500
H	-7.28048700	-2.34655000	0.24202400
H	-6.58522900	-3.97269500	0.17160200
C	-6.39274300	-1.45682900	-2.19730300
H	-7.26967500	-1.78775700	-2.75651000
H	-5.66518700	-1.07668100	-2.91570300
H	-6.69609400	-0.62967800	-1.55210800
C	-5.41193600	-3.76893400	-2.30177300
H	-4.99621000	-4.60230900	-1.73348700
H	-4.66422700	-3.45498200	-3.03071800
H	-6.28136300	-4.13185700	-2.85285200
C	-0.63554100	-0.74323200	-2.45794500
C	0.78558500	-0.70559700	-2.46297400
C	1.46881200	-0.74901900	-3.67187700
H	2.54304100	-0.67693900	-3.69626000
C	0.76226600	-0.85108900	-4.86661100
H	1.30489200	-0.88504100	-5.80125700
C	-0.62334100	-0.88684600	-4.86175200
H	-1.17015200	-0.94856300	-5.79255100
C	-1.32492700	-0.82224500	-3.66148500
H	-2.40179600	-0.80601800	-3.67685000
C	2.56269200	-1.03804600	-0.65585100
C	2.52942400	-1.10706400	0.74220300
C	3.59482200	-1.60857000	1.48812800
C	4.72708600	-1.97135100	0.75674700
H	5.58281900	-2.34081100	1.29714600
C	4.80906800	-1.89610600	-0.63546200
C	3.70237200	-1.43600100	-1.34377500
H	3.72708500	-1.39792300	-2.41606700
C	6.09911800	-2.32891000	-1.33919200
C	6.00732100	-2.18599700	-2.86269500
H	6.94686400	-2.50339500	-3.31599300
H	5.83171700	-1.15092400	-3.16324500
H	5.21449000	-2.80832200	-3.28161500
C	7.26596800	-1.45524400	-0.84306000
H	8.19567900	-1.75309900	-1.33135300
H	7.41002800	-1.54785300	0.23340000
H	7.08552000	-0.40242000	-1.06931000
C	6.38876900	-3.80528700	-1.01099800
H	5.57206200	-4.44484900	-1.34959200
H	6.51662100	-3.96315100	0.05982200
H	7.30497900	-4.12959800	-1.50790700
C	3.50635200	-1.78585500	3.00855800
C	2.35991300	-2.76372800	3.33513700
H	2.30270900	-2.92045200	4.41394200
H	2.53525900	-3.73142400	2.86225300
H	1.39814400	-2.38454200	2.99691500
C	4.80144500	-2.36762000	3.58873600
H	4.68850600	-2.48461200	4.66695200
H	5.65712200	-1.71260800	3.41603900
H	5.02849300	-3.35026700	3.17332700
C	3.24884100	-0.43435300	3.70014500
H	2.30673500	0.00789100	3.38443800
H	4.05535600	0.26925300	3.48610700

H	3.20823200	-0.57730600	4.78145900
C	-0.02016100	1.65360600	0.27021800
C	-0.87966600	2.72473300	0.21069800
C	0.52355300	2.91756900	0.20698400
C	-3.19420200	2.09535500	0.15855900
H	-2.69320500	1.16861900	0.41379000
C	-4.22910800	2.36685900	1.24115500
H	-4.82807100	3.25455300	1.03422200
H	-3.75891800	2.47671900	2.21761500
H	-4.90821200	1.51614300	1.28957100
C	-3.80382600	1.95074800	-1.22927800
H	-4.52501100	1.13491500	-1.22141200
H	-3.03764000	1.71438500	-1.96804300
H	-4.32486700	2.85816300	-1.53940800
C	-2.49839200	4.55233000	-0.02528500
H	-3.58072100	4.52767900	-0.12834000
C	-1.93871600	5.11531600	-1.32784000
H	-0.85086300	5.10607500	-1.35173100
H	-2.26585300	6.14713800	-1.45648100
H	-2.29593600	4.53577300	-2.17869600
C	-2.17996400	5.37065700	1.22169200
H	-2.72281400	4.98173600	2.08253400
H	-2.47471800	6.40955700	1.07290600
H	-1.11843000	5.35448200	1.46543400
C	1.44493400	5.16951300	0.17715900
H	0.37792500	5.36270900	0.20135400
C	2.05560100	5.74295200	1.45153300
H	1.61064600	5.29016000	2.33797600
H	1.87596300	6.81759500	1.48881800
H	3.13422400	5.58919400	1.49125700
C	2.01150500	5.79768900	-1.09099900
H	3.09031600	5.66116700	-1.16745600
H	1.81759300	6.87036100	-1.08021200
H	1.54771700	5.37548700	-1.98278800
C	2.93997400	3.09635700	0.07815400
H	3.60929300	3.95339200	0.10032800
C	3.22669800	2.24086800	1.30206200
H	3.17102600	2.83451500	2.21462100
H	4.22725600	1.81675100	1.22735400
H	2.53070200	1.41275000	1.39318400
C	3.13436800	2.37414100	-1.24880700
H	2.42114700	1.56188300	-1.36875900
H	4.13417600	1.94307200	-1.29026100
H	3.01950100	3.06174800	-2.08661300

9

O	-1.27361400	-1.36668300	-0.56454500
O	1.24959600	-1.33607100	-0.46004700
N	-1.26253200	1.15197000	-0.59475200
N	1.24742600	1.17967800	-0.55839300
C	-2.45497400	-0.79929200	-0.25858100
C	-2.48959900	0.61970100	-0.25824600
C	-3.65797100	1.28388000	0.10523300
H	-3.67879600	2.35868100	0.13915900
C	-4.79709600	0.56314800	0.45062700

C	-4.73557800	-0.83157200	0.41802900
H	-5.61749400	-1.38928300	0.67961100
C	-3.58749600	-1.54898200	0.07192300
C	-3.55020200	-3.08080900	0.07286800
C	-2.50106200	-3.57436000	1.08876000
H	-1.50562700	-3.21296100	0.84229700
H	-2.47906200	-4.66657100	1.09820600
H	-2.75326400	-3.23239900	2.09461000
C	-4.90373200	-3.68679500	0.46443800
H	-5.20584400	-3.39409400	1.47155300
H	-4.82616300	-4.77490100	0.44652500
H	-5.69537600	-3.40009200	-0.23025300
C	-3.19547000	-3.59391200	-1.33615400
H	-3.94009900	-3.26281300	-2.06297500
H	-3.18021200	-4.68617900	-1.34089400
H	-2.21915400	-3.23739100	-1.65511100
C	-6.06742500	1.32106600	0.85142400
C	-6.51308400	2.22770300	-0.31080300
H	-7.41935700	2.77455600	-0.04083200
H	-6.72299700	1.63605100	-1.20349100
H	-5.74606700	2.95883600	-0.56795600
C	-7.22777600	0.37856900	1.19117600
H	-8.10487200	0.96591200	1.46774200
H	-6.98668700	-0.27290000	2.03267700
H	-7.50276500	-0.24723100	0.34055600
C	-5.77610900	2.18797500	2.09048100
H	-4.98701900	2.91513000	1.89687200
H	-5.45856900	1.56685800	2.92971500
H	-6.67188100	2.73695700	2.38970100
C	-0.74121900	2.40444900	-0.53762500
C	0.70514000	2.42092500	-0.51396300
C	1.37212400	3.65456700	-0.44737200
H	2.44783200	3.69080000	-0.46714800
C	0.65306900	4.83206600	-0.39286800
H	1.17990700	5.77578700	-0.34756400
C	-0.74561100	4.81606100	-0.42555400
H	-1.29485600	5.74794900	-0.40862500
C	-1.43632000	3.62248700	-0.50695100
H	-2.51033800	3.63414200	-0.57989800
C	2.43317900	-0.76310400	-0.19659600
C	3.57745100	-1.51035600	0.12413000
C	4.72635400	-0.78697800	0.42449100
H	5.61832900	-1.33442200	0.68318500
C	4.79567000	0.61509100	0.41763600
C	3.65822000	1.32980800	0.08171200
H	3.67980800	2.40301300	0.07501100
C	2.47310700	0.65558100	-0.23167100
C	6.11648400	1.30013000	0.77833100
C	6.52276300	0.90393700	2.21004600
H	5.76050700	1.21343500	2.92712400
H	7.46548000	1.38335800	2.48324100
H	6.65495800	-0.17373700	2.30690400
C	6.01557200	2.82842500	0.71601800
H	5.75874400	3.17554200	-0.28657800
H	6.97764200	3.26936500	0.98128200
H	5.27172300	3.21317600	1.41604100

C	7.21213500	0.85099700	-0.20628000
H	7.36406800	-0.22797700	-0.17527600
H	8.16237200	1.33017600	0.03990000
H	6.94660100	1.12241900	-1.22947000
C	3.53807500	-3.04126000	0.15864800
C	3.15199200	-3.58257000	-1.23150200
H	2.16795400	-3.23400100	-1.53491700
H	3.13812600	-4.67459500	-1.21338600
H	3.87942600	-3.26589100	-1.98176800
C	2.51104300	-3.51177400	1.20740400
H	2.78433100	-3.14795100	2.19998600
H	2.49008000	-4.60340600	1.24107500
H	1.51026500	-3.15655200	0.97417500
C	4.89975200	-3.63949300	0.53304000
H	5.67584800	-3.36829900	-0.18513200
H	4.82182700	-4.72759300	0.53997700
H	5.22366700	-3.32590900	1.52709400
As	-0.00192500	-0.20443500	-1.30477800

9^Me

O	1.29759200	-1.84279800	0.33528300
C	2.45680300	-1.24715100	0.02173900
C	3.60437500	-2.01211400	-0.29030200
C	4.76924400	-1.31998100	-0.65317500
H	5.66906400	-1.90479800	-0.90496000
C	4.83064000	0.09357700	-0.71425700
C	3.68951800	0.84082900	-0.38492800
H	3.73430500	1.93440400	-0.44367700
C	2.49556300	0.18776800	-0.00446000
N	1.27070400	0.72586500	0.32899300
C	0.72901400	1.97963300	0.24679800
C	1.41753000	3.21329100	0.16531900
H	2.51194900	3.23294300	0.21071100
C	0.70526600	4.41138400	0.06683500
H	1.25424000	5.36310300	0.01223200
C	-0.70527200	4.41138400	0.06683900
H	-1.25424700	5.36310200	0.01223900
C	-1.41753300	3.21328900	0.16532500
H	-2.51195300	3.23293900	0.21072300
C	-0.72901500	1.97963200	0.24679900
N	-1.27070500	0.72586300	0.32899400
C	-2.49556300	0.18776600	-0.00446000
C	-3.68951500	0.84082700	-0.38493800
H	-3.73430000	1.93440100	-0.44369300
C	-4.83063600	0.09357400	-0.71426900
C	-4.76924000	-1.31998400	-0.65318600
H	-5.66905800	-1.90480100	-0.90497500
C	-3.60437300	-2.01211600	-0.29030600
C	-2.45680300	-1.24715300	0.02174000
O	-1.29759100	-1.84279900	0.33528400
C	3.51985700	-3.51206500	-0.24107900
C	6.11026500	0.77617000	-1.12951900
C	-3.51985200	-3.51206800	-0.24109300
C	-6.11027200	0.77616700	-1.12949800
As	0.00000000	-0.64007500	1.09366300

H	4.48978800	-3.97996700	-0.49520600
H	3.21019700	-3.86128200	0.76586400
H	2.74954700	-3.89042700	-0.94503800
H	6.95423000	0.49244100	-0.46609700
H	6.40566400	0.48933600	-2.16090800
H	6.01480300	1.87885600	-1.10138600
H	-6.40587200	0.48908600	-2.16075800
H	-6.95414800	0.49268800	-0.46585500
H	-6.01471200	1.87885300	-1.10166500
H	-3.21011700	-3.86128700	0.76582600
H	-4.48980100	-3.97996900	-0.49515200
H	-2.74959400	-3.89042900	-0.94511100

TS I

O	-1.26483400	-2.43931700	-1.15965500
C	-3.48283900	-3.24931100	-0.87122800
C	-2.54582300	-2.19203900	-1.10974200
C	-4.84444400	-2.95339500	-0.90583200
H	-5.56756100	-3.76770700	-0.73359800
C	-5.33475700	-1.64416500	-1.16569200
C	-4.42185300	-0.60235300	-1.36374200
H	-4.80712300	0.39447900	-1.60544700
C	-3.02663200	-0.83838600	-1.30999600
N	-2.00365700	0.07876500	-1.47575700
C	-1.99241900	1.44801000	-1.45643600
C	-3.08378100	2.29359600	-1.12046400
H	-4.02369100	1.85134000	-0.77667700
C	-2.94613900	3.67604800	-1.15298800
H	-3.80081400	4.31200700	-0.87843300
C	-1.72029300	4.26139600	-1.54344100
H	-1.63141400	5.35481000	-1.62632600
C	-0.61451100	3.46361000	-1.82346200
H	0.31543600	3.93779000	-2.14746700
C	-0.69730400	2.04765500	-1.73947900
N	0.33718200	1.15309400	-1.90320100
C	1.71882400	1.33697300	-1.86521700
C	2.43915300	2.54637600	-1.71843400
H	1.92138100	3.50060000	-1.59171100
C	3.83659800	2.54766400	-1.65237700
C	4.53874200	1.31867800	-1.75595100
H	5.64083200	1.32756300	-1.72471500
C	3.88090800	0.09536800	-1.88415500
C	2.45493600	0.09767600	-1.90097500
O	1.79162700	-1.04347000	-1.92640500
C	-2.93582900	-4.62020000	-0.59984800
C	-6.82006800	-1.39440700	-1.21118300
C	4.59525200	3.83806400	-1.48266200
C	4.59423400	-1.22003100	-2.01247900
O	-1.41724300	-1.04795900	1.62022200
C	-2.15372600	0.05730800	1.89949300
C	-3.50823600	-0.04295500	2.26302100
C	-4.19550500	1.15859500	2.52572000
H	-5.25794400	1.10006600	2.81242700
C	-3.57607300	2.42213300	2.44158700
C	-2.21788100	2.49730100	2.07779600

H	-1.74060000	3.47968900	1.99366900
C	-1.49907300	1.32199100	1.79996800
N	-0.17572700	1.16618500	1.41019800
C	0.93588700	1.97678900	1.49182900
C	0.96131000	3.38153900	1.59791500
H	0.02732700	3.95152700	1.56287200
C	2.18413500	4.05112900	1.74101900
H	2.19106300	5.14657400	1.84271800
C	3.39240500	3.33367000	1.75423200
H	4.34833500	3.86465100	1.87161000
C	3.39799800	1.94310200	1.58849100
H	4.35106200	1.40748600	1.56531800
C	2.18138800	1.24171000	1.45291400
N	2.00191000	-0.11595300	1.28797800
C	2.85007400	-1.20954800	1.32529000
C	4.25681000	-1.26238900	1.38594700
H	4.85424700	-0.34533200	1.41710800
C	4.91740000	-2.50336000	1.37784600
C	4.15683800	-3.69129900	1.28611800
H	4.67985000	-4.66100300	1.26213700
C	2.75408300	-3.68571600	1.21288000
C	2.10850100	-2.43334000	1.26788100
O	0.76632100	-2.34623300	1.25364800
C	-4.15269700	-1.39492400	2.37796600
C	-4.34715600	3.68917100	2.71906700
C	1.92204400	-4.93259100	1.10240000
C	6.42491200	-2.55942300	1.39053500
As	0.18629300	-0.62709700	0.74591000
As	-0.23635800	-0.75901200	-2.03837900
H	-7.32747100	-2.07998800	-1.92175200
H	-7.28505600	-1.56693500	-0.21655100
H	-7.05282200	-0.35514000	-1.51364200
H	-3.74411600	-5.35385600	-0.41891900
H	-2.31223800	-4.97320300	-1.44747700
H	-2.26203700	-4.59975600	0.28252400
H	-5.22816000	-1.30677100	2.62355500
H	-4.05316000	-1.96589900	1.43366100
H	-3.66199300	-2.00417600	3.16561000
H	-5.41152400	3.47904900	2.94001900
H	-3.92650300	4.24231600	3.58530100
H	-4.31052900	4.38157800	1.85097100
H	5.39518300	3.73777300	-0.72081100
H	5.08996900	4.14479700	-2.42983300
H	3.92897600	4.66351600	-1.16594500
H	4.44671100	-1.64815000	-3.02687000
H	5.68128400	-1.10875300	-1.83579400
H	4.18807600	-1.96362100	-1.29892300
H	6.86372900	-1.69214500	1.92252400
H	6.79639600	-3.48365000	1.87560500
H	6.83234000	-2.54849800	0.35545500
H	1.19016700	-4.83952600	0.27372900
H	2.55262000	-5.82530100	0.93018300
H	1.32463700	-5.09973000	2.02357500

Int A

O	1.01010500	-2.20779800	1.70058300
C	3.23208800	-3.14291000	1.66062400
C	2.36761500	-2.03861200	1.61020300
C	4.61213100	-2.88927600	1.51758300
H	5.30564400	-3.74508300	1.54003800
C	5.12544200	-1.59401500	1.31732400
C	4.23668100	-0.50013200	1.28462400
H	4.62703900	0.50227000	1.07660600
C	2.85900300	-0.70951100	1.44928700
N	1.81795800	0.22268900	1.41799800
C	1.74076500	1.60472100	1.56506500
C	2.81405500	2.50877300	1.64620000
H	3.84496300	2.14457500	1.59572400
C	2.56453500	3.88291500	1.79072600
H	3.41352800	4.58055300	1.84157600
C	1.24914500	4.36185000	1.86672500
H	1.05564300	5.43922500	1.97355100
C	0.16310500	3.47443900	1.80302500
H	-0.85841300	3.86163300	1.86846300
C	0.38941300	2.09627600	1.64662300
N	-0.57089800	1.08982400	1.56335400
C	-1.95328500	1.03809500	1.76812800
C	-2.88581500	2.08737100	1.76242700
H	-2.56594200	3.11802900	1.57320500
C	-4.25766600	1.81354900	1.94511400
C	-4.67290100	0.48181300	2.12520300
H	-5.74508800	0.26601900	2.26058400
C	-3.77074500	-0.60248100	2.10868600
C	-2.41317600	-0.30329600	1.92101800
O	-1.48011300	-1.30664800	1.83983300
C	2.66658600	-4.52563500	1.83395600
C	6.59870200	-1.36274700	1.08806700
C	-5.25222500	2.94859600	1.92148000
C	-4.21389700	-2.03438700	2.21926800
O	1.48011400	-1.30664700	-1.83983100
C	2.41317700	-0.30329500	-1.92101500
C	3.77074500	-0.60248000	-2.10868100
C	4.67290100	0.48181500	-2.12519800
H	5.74508900	0.26602100	-2.26057800
C	4.25766500	1.81355000	-1.94511000
C	2.88581400	2.08737100	-1.76242800
H	2.56593900	3.11803000	-1.57320800
C	1.95328500	1.03809500	-1.76812800
N	0.57089800	1.08982400	-1.56335800
C	-0.38941300	2.09627500	-1.64662700
C	-0.16310500	3.47443800	-1.80303000
H	0.85841300	3.86163300	-1.86846900
C	-1.24914500	4.36185000	-1.86673000
H	-1.05564300	5.43922500	-1.97355700
C	-2.56453500	3.88291400	-1.79073100
H	-3.41352800	4.58055200	-1.84158200
C	-2.81405500	2.50877400	-1.64620400
H	-3.84496200	2.14457500	-1.59572800
C	-1.74076500	1.60472100	-1.56506800
N	-1.81795800	0.22268900	-1.41799900
C	-2.85900300	-0.70951100	-1.44928700

C	-4.23668100	-0.50013200	-1.28461900
H	-4.62703700	0.50227000	-1.07659500
C	-5.12544200	-1.59401500	-1.31731900
C	-4.61213100	-2.88927500	-1.51758200
H	-5.30564500	-3.74508200	-1.54003700
C	-3.23208900	-3.14290900	-1.66062700
C	-2.36761500	-2.03861100	-1.61020600
O	-1.01010500	-2.20779700	-1.70058800
C	4.21389800	-2.03438600	-2.21926500
C	5.25222400	2.94859700	-1.92146200
C	-2.66658700	-4.52563500	-1.83395900
C	-6.59870100	-1.36274600	-1.08806200
As	-0.14434400	-0.62989700	-1.24994700
As	0.14434400	-0.62989600	1.24994300
H	7.18194900	-2.29593500	1.20849800
H	6.78445000	-0.97790800	0.06234800
H	7.01035700	-0.61038600	1.79231400
H	3.46843000	-5.28765000	1.85349600
H	2.08202500	-4.60659300	2.77380000
H	1.96479400	-4.77373500	1.01050200
H	5.27917200	-2.10141300	-2.51143800
H	4.09103000	-2.55469300	-1.24546100
H	3.60486400	-2.59141600	-2.95936800
H	6.29447500	2.57812300	-1.97012700
H	5.10196200	3.64185400	-2.77601800
H	5.14818700	3.55468300	-0.99666300
H	-5.14812800	3.55474700	0.99673100
H	-6.29447800	2.57811800	1.97005300
H	-5.10202000	3.64179200	2.77609500
H	-3.60485600	-2.59142000	2.95936300
H	-5.27916900	-2.10141500	2.51145200
H	-4.09104000	-2.55469000	1.24546100
H	-7.01036500	-0.61041700	-1.79233900
H	-7.18194300	-2.29594200	-1.20845200
H	-6.78444600	-0.97786600	-0.06235900
H	-1.96481700	-4.77374600	-1.01049000
H	-3.46843300	-5.28764600	-1.85352600
H	-2.08200300	-4.60658500	-2.77378900

TS A

O	1.13384900	-2.23248100	1.58866400
C	3.39309700	-3.01960100	1.72193200
C	2.46353400	-1.97147100	1.55446400
C	4.75856000	-2.71004200	1.59269600
H	5.49139200	-3.52863500	1.67759100
C	5.21488300	-1.40361600	1.32242700
C	4.27705100	-0.36040400	1.22469500
H	4.62604500	0.64457000	0.96432600
C	2.90178000	-0.62295100	1.36039100
N	1.83847400	0.28432800	1.29536300
C	1.75236100	1.65569900	1.49026700
C	2.82851900	2.55590700	1.63835800
H	3.85848500	2.19085200	1.59529000
C	2.58571200	3.92017400	1.82967000
H	3.43680200	4.61208700	1.91283300

C	1.26861500	4.40283300	1.92372300
H	1.08133600	5.47536200	2.07987700
C	0.18337900	3.52608700	1.82543700
H	-0.83482700	3.91208400	1.92825200
C	0.39772100	2.15306300	1.58170400
N	-0.57446500	1.17167200	1.45213000
C	-1.95870200	1.16493700	1.65910500
C	-2.84360300	2.25806200	1.63883900
H	-2.48000300	3.26190300	1.39320100
C	-4.22331800	2.06135900	1.83529100
C	-4.70010600	0.75860500	2.08164500
H	-5.78009600	0.60171300	2.23587400
C	-3.85301700	-0.36438700	2.10279800
C	-2.48346500	-0.15399900	1.84358800
O	-1.64373500	-1.21463100	1.73540000
C	2.88919500	-4.41489600	1.96664500
C	6.67671300	-1.12526400	1.07444700
C	-5.16420700	3.23839600	1.75068200
C	-4.35736600	-1.76646400	2.29667700
O	1.46640100	-1.37711600	-1.71935800
C	2.37596000	-0.36836900	-1.90137500
C	3.73263100	-0.65612500	-2.11071200
C	4.61262000	0.43963300	-2.22700100
H	5.68425700	0.23427900	-2.38128100
C	4.17659300	1.77348700	-2.12240700
C	2.80543000	2.03501500	-1.91781900
H	2.47061900	3.07115900	-1.79458900
C	1.89334600	0.97121100	-1.82689300
N	0.51680700	1.00644900	-1.59363700
C	-0.46795300	1.98090700	-1.72757200
C	-0.27750800	3.35563800	-1.95164200
H	0.73383700	3.76584300	-2.03485400
C	-1.38613000	4.20946000	-2.06681300
H	-1.22088100	5.28422800	-2.23192400
C	-2.68879100	3.69975500	-1.97099200
H	-3.55606900	4.37021700	-2.06296000
C	-2.90198500	2.32939700	-1.75205600
H	-3.92339700	1.94382800	-1.67783900
C	-1.80631300	1.45892400	-1.62064500
N	-1.84717800	0.08715300	-1.39057700
C	-2.86110100	-0.87376200	-1.39137100
C	-4.24953500	-0.69761900	-1.28862700
H	-4.67969400	0.30314900	-1.17162500
C	-5.10346700	-1.81978400	-1.27227000
C	-4.54497400	-3.10893500	-1.35897400
H	-5.21133900	-3.98613700	-1.34055900
C	-3.15379300	-3.32684400	-1.43935900
C	-2.32612100	-2.19402700	-1.44505600
O	-0.96360300	-2.32200500	-1.48899900
C	4.19770200	-2.08483400	-2.14323400
C	5.14968800	2.92395100	-2.21152700
C	-2.53984800	-4.69853400	-1.49371800
C	-6.59041400	-1.62034800	-1.11063700
As	-0.15210400	-0.69947400	-1.12430500
As	0.14165300	-0.60702400	1.34906200
H	7.31849600	-1.94558800	1.45060300

H	6.87971200	-1.01636300	-0.01345400
H	7.00455100	-0.18316000	1.55847800
H	3.72434200	-5.11780500	2.14715200
H	2.20393100	-4.44462400	2.83870600
H	2.30088300	-4.78367300	1.10024100
H	5.26320900	-2.15200500	-2.43453400
H	4.08274900	-2.55377200	-1.14316900
H	3.59589100	-2.69063200	-2.85046600
H	6.19769600	2.56859900	-2.24886000
H	4.97234000	3.53983600	-3.11871400
H	5.05087700	3.60391500	-1.33922800
H	-5.07890900	3.74919000	0.76788400
H	-6.21874400	2.92732900	1.88083000
H	-4.93916400	3.99973900	2.52689500
H	-3.75868100	-2.30612000	3.05826300
H	-5.41929900	-1.77008200	2.60824400
H	-4.26909700	-2.34758400	1.35423000
H	-6.99729200	-0.92761100	-1.87618400
H	-7.14149400	-2.57714100	-1.19090800
H	-6.82640400	-1.17771400	-0.11891500
H	-1.84527800	-4.85536700	-0.64231000
H	-3.31532600	-5.48716200	-1.46575900
H	-1.93556800	-4.83295700	-2.41489600

Int I

O	-1.41814300	-2.44591300	-1.07081800
C	-3.69376600	-3.13236200	-1.05269400
C	-2.66981900	-2.12443700	-1.13466900
C	-5.02314500	-2.74700200	-1.18646800
H	-5.80894100	-3.51783400	-1.12126300
C	-5.40648300	-1.39554200	-1.42549500
C	-4.42003500	-0.40821200	-1.49047300
H	-4.72291500	0.61804800	-1.72468000
C	-3.05068800	-0.73137700	-1.31146500
N	-1.97193300	0.13136700	-1.35374500
C	-1.93003800	1.49998500	-1.37125400
C	-3.01121900	2.37432100	-1.06909800
H	-3.95093300	1.95654900	-0.69489200
C	-2.85791600	3.75126700	-1.16017500
H	-3.70046400	4.40935500	-0.90059500
C	-1.62799900	4.30343400	-1.59011100
H	-1.52952500	5.38997900	-1.73178100
C	-0.53037200	3.48294400	-1.82353100
H	0.40609500	3.92702700	-2.17112800
C	-0.62336600	2.07280600	-1.65255600
N	0.40919200	1.17154000	-1.74346400
C	1.78608400	1.37403300	-1.77242400
C	2.49011100	2.59518500	-1.63097000
H	1.96145000	3.52650100	-1.40929600
C	3.88708300	2.62524200	-1.66081400
C	4.60468800	1.41628100	-1.86911000
H	5.70577000	1.45043300	-1.91822200
C	3.96635200	0.18320800	-1.98665000
C	2.54147200	0.14777200	-1.87473500
O	1.90581200	-0.99960000	-1.84028800

C	-3.25665600	-4.54792400	-0.81741300
C	-6.86233700	-1.05349600	-1.60724800
C	4.63510500	3.91689300	-1.45861400
C	4.69485900	-1.11303400	-2.19524700
O	-1.42349600	-1.15048100	1.62549200
C	-2.17444200	-0.05171800	1.91914900
C	-3.52897100	-0.17158000	2.27022400
C	-4.22967700	1.02023400	2.54719700
H	-5.29399900	0.94792100	2.82312800
C	-3.62051100	2.28825800	2.48437000
C	-2.26001800	2.38053200	2.12706800
H	-1.79262300	3.36861600	2.05485100
C	-1.52970400	1.21639900	1.83885500
N	-0.20329500	1.07949700	1.43858000
C	0.90754100	1.90098100	1.54395500
C	0.91910200	3.30062200	1.68363100
H	-0.02031600	3.86257900	1.67105600
C	2.13813200	3.98094400	1.83086300
H	2.13412500	5.07422200	1.95280100
C	3.35021700	3.27488500	1.82299000
H	4.30320700	3.80997100	1.94624000
C	3.36520200	1.88496600	1.63416200
H	4.32189300	1.35584100	1.60314400
C	2.15575200	1.18122700	1.48195600
N	1.98810400	-0.17893000	1.27288300
C	2.85129100	-1.26738300	1.26495300
C	4.25665100	-1.30169200	1.27143400
H	4.84224000	-0.37632100	1.28178300
C	4.93269100	-2.53711400	1.22470300
C	4.18483000	-3.73038500	1.15235600
H	4.71672100	-4.69396700	1.10070700
C	2.77750800	-3.73957200	1.12874700
C	2.12488000	-2.49679600	1.20731400
O	0.77269000	-2.42086500	1.21640300
C	-4.16972300	-1.52921100	2.33312600
C	-4.40195700	3.54611200	2.77553200
C	1.95844200	-4.99681100	1.03585400
C	6.44116800	-2.56283500	1.19507900
As	0.18832400	-0.69531400	0.82323000
As	-0.16684400	-0.75721500	-1.77150800
H	-7.32140200	-1.65060500	-2.42319200
H	-7.44369800	-1.27597200	-0.68678200
H	-7.00632000	0.01799200	-1.84535900
H	-4.11782400	-5.24190600	-0.78518800
H	-2.55357200	-4.87954900	-1.60947000
H	-2.69295400	-4.62785700	0.13590000
H	-5.21104000	-1.46398600	2.70179700
H	-4.18482300	-2.00758000	1.33204300
H	-3.60026700	-2.21102300	2.99740300
H	-5.46782900	3.32607600	2.97896600
H	-3.99553200	4.08496600	3.65748200
H	-4.35834700	4.25516100	1.92129400
H	5.35099600	3.83693300	-0.61378300
H	5.22964900	4.18699000	-2.35754400
H	3.94864000	4.75643400	-1.23741800
H	4.39792600	-1.58031400	-3.15772700

H	5.79169500	-0.96339700	-2.20075600
H	4.43419200	-1.84430100	-1.40390000
H	6.87786800	-1.91360700	1.98146000
H	6.83470100	-3.58725300	1.34135700
H	6.82774600	-2.19334800	0.22038000
H	1.19639500	-4.90917200	0.23435700
H	2.59503900	-5.87898300	0.83395500
H	1.39682900	-5.18091000	1.97615100

TS II

O	-1.47928800	-1.67724200	-1.46500200
C	-3.83835400	-1.98584500	-1.61270200
C	-2.67010800	-1.15680600	-1.58872100
C	-5.07356800	-1.39147300	-1.86456900
H	-5.97167100	-2.02941200	-1.91120000
C	-5.21186900	0.00286000	-2.10524400
C	-4.07705300	0.82044800	-2.04252400
H	-4.17344100	1.89025300	-2.27008800
C	-2.80665700	0.27693700	-1.73605800
N	-1.63490800	0.99652600	-1.56241400
C	-1.51793400	2.28850700	-1.14297200
C	-2.56334200	3.11658600	-0.64259800
H	-3.56395600	2.68999400	-0.50880100
C	-2.31191300	4.43830000	-0.29309600
H	-3.12880500	5.06137100	0.10065100
C	-1.02055500	4.98789300	-0.46751100
H	-0.84262400	6.05561800	-0.27217000
C	0.03927900	4.18079600	-0.87256600
H	1.02850300	4.62425600	-1.01643800
C	-0.16538300	2.80222100	-1.13815800
N	0.81580300	1.86652900	-1.40533700
C	2.19113100	1.95901000	-1.18001400
C	2.90360400	2.99833700	-0.53584800
H	2.38294400	3.85925400	-0.10807000
C	4.28297500	2.89293700	-0.33287400
C	4.96997900	1.73959400	-0.79690900
H	6.06049000	1.67162700	-0.64890800
C	4.31014400	0.67439900	-1.40945500
C	2.89689600	0.77132300	-1.56875400
O	2.18999600	-0.23418100	-2.03970500
C	-3.64817800	-3.46479000	-1.43807400
C	-6.56637700	0.57696700	-2.43077300
C	5.03656500	3.97251000	0.40131900
C	5.00226000	-0.56784800	-1.88950800
O	-1.30517700	-2.17852100	1.25850800
C	-2.26815200	-1.39357800	1.83112000
C	-3.44126400	-1.94027100	2.37629500
C	-4.37431100	-1.03755400	2.93185200
H	-5.29992200	-1.44982900	3.36539300
C	-4.17274600	0.35500000	2.95361800
C	-2.98013800	0.87805700	2.40983400
H	-2.80269600	1.96203600	2.40747100
C	-2.02186800	0.00865900	1.87067100
N	-0.77502700	0.32424500	1.32645900
C	0.23733300	1.12826300	1.82514800

C	0.08295600	2.43367400	2.32468600
H	-0.90723700	2.90562600	2.30611500
C	1.19388400	3.12479500	2.82987000
H	1.06189900	4.13818200	3.23729500
C	2.46919500	2.53558000	2.79943200
H	3.34286000	3.09002500	3.17278600
C	2.65379600	1.25365800	2.26332100
H	3.66447200	0.84061700	2.19813800
C	1.54733200	0.52533600	1.78226300
N	1.54431600	-0.75069400	1.22527300
C	2.55356900	-1.66478800	0.96612400
C	3.89668900	-1.66040600	1.39569500
H	4.26989100	-0.87600100	2.06214300
C	4.76926300	-2.68987500	1.00360600
C	4.28971700	-3.71885100	0.16135800
H	4.97861100	-4.51896700	-0.15409500
C	2.96119400	-3.76332800	-0.29047300
C	2.08893600	-2.74350500	0.14739700
O	0.79101700	-2.75492400	-0.20176500
C	-3.66484000	-3.42826800	2.37686700
C	-5.20411400	1.28518800	3.54483500
C	2.42748900	-4.84788300	-1.18322100
C	6.21595700	-2.66427000	1.42957200
As	-0.08992700	-1.15745900	0.27586200
As	0.19267800	0.12299200	-2.04497700
H	-7.01182700	0.08496500	-3.32094600
H	-7.27863300	0.42000700	-1.59278100
H	-6.51510400	1.66428200	-2.63232600
H	-4.60480500	-3.97529100	-1.21576400
H	-3.22005900	-3.91775000	-2.35793600
H	-2.91587600	-3.66774300	-0.63214400
H	-4.48019200	-3.70888900	3.07088800
H	-3.93618100	-3.79679100	1.36601200
H	-2.74444400	-3.97127300	2.67124100
H	-6.09658200	0.73190300	3.89615400
H	-4.79633300	1.84989400	4.40997800
H	-5.54522200	2.03850800	2.80347400
H	5.53058800	3.57018700	1.31139100
H	5.83984400	4.40994300	-0.22867700
H	4.36722100	4.79537600	0.71804900
H	4.90787700	-0.67236600	-2.99090800
H	6.07787200	-0.55674200	-1.62892500
H	4.53754100	-1.47324100	-1.45076300
H	6.35904200	-2.08707400	2.36428300
H	6.61379300	-3.68579200	1.59196500
H	6.85123200	-2.18878200	0.64993300
H	1.95943200	-4.41390900	-2.09091000
H	3.22834100	-5.54540800	-1.49343300
H	1.63020700	-5.42951600	-0.67500800

Int II

O	1.31390700	-0.92532700	1.40412000
C	3.11386000	0.20495800	2.55129100
C	1.86499900	0.24563500	1.88729300
C	3.61124600	1.39535500	3.10931300

H	4.57925400	1.36312600	3.63530000
C	2.90710100	2.61224500	3.03514500
C	1.66316500	2.62128000	2.38556100
H	1.07015800	3.54684700	2.34906400
C	1.13031800	1.45718300	1.79147100
N	-0.14288900	1.46494100	1.18355400
C	-0.56120900	2.45495600	0.33719100
C	0.25578700	3.44179000	-0.27128800
H	1.34073100	3.41141300	-0.10722700
C	-0.31681100	4.42071700	-1.07670200
H	0.32492700	5.17362700	-1.55767100
C	-1.71520900	4.45811800	-1.26261400
H	-2.17350400	5.26932300	-1.84722400
C	-2.53310200	3.47271600	-0.70776700
H	-3.61547000	3.55504500	-0.83213900
C	-1.96960400	2.41924900	0.05385400
N	-2.63047300	1.33783500	0.63391700
C	-3.95551600	0.93948800	0.43680000
C	-4.90407500	1.43225600	-0.49387700
H	-4.63841000	2.19799700	-1.22799600
C	-6.20431900	0.91084300	-0.53068500
C	-6.55672200	-0.14154500	0.35241100
H	-7.57994100	-0.55047400	0.31507400
C	-5.63645300	-0.71152500	1.23225400
C	-4.32071000	-0.16697100	1.26994300
O	-3.38365500	-0.66473600	2.04315300
C	3.89278900	-1.07864700	2.66857300
C	3.47526400	3.86982400	3.64497200
C	-7.21739500	1.43332400	-1.51930400
C	-5.94440300	-1.89362000	2.10493100
O	3.00067600	-2.40575900	-0.30197000
C	4.07037100	-1.62599900	-0.65125400
C	5.38096400	-2.11646000	-0.54421300
C	6.42858000	-1.23138000	-0.87226900
H	7.46523900	-1.59331200	-0.78182900
C	6.19814200	0.09328300	-1.28863000
C	4.86992300	0.55541000	-1.40013200
H	4.68347400	1.59816300	-1.68485000
C	3.80035100	-0.30393300	-1.10484700
N	2.42463000	-0.03977000	-1.15077100
C	1.65844400	0.75130400	-2.01221000
C	2.12196700	1.80650300	-2.81530700
H	3.18049000	2.08686100	-2.79780000
C	1.22710300	2.50326500	-3.64159000
H	1.59808200	3.33885700	-4.25292800
C	-0.12768800	2.14636300	-3.67886600
H	-0.83261200	2.70649600	-4.30973600
C	-0.60406500	1.07587300	-2.90779400
H	-1.66266700	0.80388000	-2.95653100
C	0.27493400	0.36624500	-2.07173200
N	-0.02959400	-0.73687400	-1.27405000
C	-1.14723100	-1.57202600	-1.15267400
C	-2.46619000	-1.36898900	-1.59626100
H	-2.75607200	-0.44253500	-2.10312200
C	-3.44502500	-2.35822700	-1.37337400
C	-3.09349900	-3.52590600	-0.66978900

H	-3.86440800	-4.28912400	-0.47687000
C	-1.78757400	-3.76097200	-0.19908400
C	-0.82666000	-2.77236700	-0.45763600
O	0.47035200	-2.93625900	-0.04868900
C	5.61535600	-3.52220000	-0.06433900
C	7.34513400	1.01647900	-1.62111700
C	-1.40002600	-4.99604100	0.56579000
C	-4.83821700	-2.17792200	-1.92144000
As	1.44164500	-1.42103900	-0.34098100
As	-1.58910600	0.21516700	1.81578400
H	3.84091800	3.69133300	4.67671600
H	4.34139700	4.24315800	3.05782000
H	2.72375700	4.68187300	3.68321400
H	4.35852000	-1.16374300	3.67002700
H	3.25137200	-1.96102900	2.49340100
H	4.71287800	-1.10879100	1.92054300
H	6.69008100	-3.78302600	-0.09429900
H	5.25223200	-3.65387800	0.97696600
H	5.05583800	-4.25537600	-0.68074600
H	8.31868200	0.57383700	-1.33482700
H	7.38582200	1.23695200	-2.70919900
H	7.24829000	1.99091800	-1.09926500
H	-7.56578700	0.63234100	-2.20581300
H	-8.12008500	1.82503500	-1.00475300
H	-6.80235200	2.25166500	-2.13923500
H	-5.71254300	-1.67994400	3.16854200
H	-7.00587200	-2.19552800	2.02247800
H	-5.30495700	-2.75749700	1.82253500
H	-4.91774900	-2.59883900	-2.94741600
H	-5.59170600	-2.69143700	-1.29327100
H	-5.11723800	-1.10832800	-1.97352800
H	-1.05277700	-4.73678100	1.58773100
H	-2.25173600	-5.69664500	0.65344100
H	-0.55654200	-5.52454500	0.07547500

TS III

As	-0.82248900	1.00230000	-0.89016500
As	0.76907000	0.19962700	2.06000400
O	-1.35915000	2.08059400	0.52427900
O	2.72250900	0.28187800	2.31161600
O	-2.56407100	0.56073000	-1.32670900
O	-0.72930800	2.46594600	-2.01677600
N	-1.11456200	-0.45713400	1.42890500
N	1.19892300	-1.55910900	1.44059400
N	-0.34619500	-0.59487000	-1.77152000
N	1.06136500	1.23599700	-0.56681200
C	-2.31858800	1.61101800	1.37133500
C	-2.22482500	0.28946100	1.87045400
C	-3.20131800	-0.17317600	2.77582700
H	-3.11236000	-1.18970700	3.18629300
C	-4.26959000	0.65262600	3.16424700
C	-4.35146000	1.95405600	2.62172100
H	-5.19332300	2.60472600	2.90932000
C	-3.39913100	2.45126900	1.71731800
C	-3.48775900	3.82087500	1.10268800

C	-5.29676900	0.17353000	4.16089700
C	-1.15200100	-1.81450100	1.17213500
C	0.13680700	-2.43653900	1.16914800
C	0.22248600	-3.83417600	0.98543800
H	1.18358100	-4.34834900	1.07965000
C	-0.93621400	-4.58391000	0.75106000
H	-0.85311500	-5.67311400	0.62112300
C	-2.19729200	-3.95923300	0.70388400
H	-3.10190000	-4.55036800	0.49991800
C	-2.30847700	-2.58620800	0.92401700
H	-3.28667300	-2.08962300	0.89241400
C	2.56480400	-1.81986800	1.29430400
C	3.18839400	-2.89780400	0.62889500
H	2.59663100	-3.65979600	0.11212800
C	4.58607300	-2.95332500	0.53541900
C	5.36401000	-1.92215900	1.11569200
H	6.46281600	-1.97958900	1.04761900
C	4.78727400	-0.81132300	1.74074500
C	3.37237300	-0.76049000	1.81124900
C	5.58799600	0.33313900	2.29494700
C	5.24913800	-4.05692000	-0.24982900
C	-2.66599800	-0.71661900	-1.79093100
C	-1.44548500	-1.40207900	-2.05237500
C	-1.48323000	-2.75225700	-2.43614200
H	-0.55793400	-3.32318700	-2.57263400
C	-2.72419500	-3.40320300	-2.58566600
C	-3.91325000	-2.69269000	-2.32924200
H	-4.88110200	-3.20742000	-2.44048300
C	-3.91322600	-1.34789000	-1.90763100
C	-5.16357800	-0.59196900	-1.55128200
C	-2.75584700	-4.85909800	-2.98045300
C	1.03287900	-0.79013500	-1.87034400
C	1.69132400	-1.81313800	-2.57774100
H	1.11349900	-2.57618500	-3.11025800
C	3.09376200	-1.82003300	-2.65089300
H	3.59627000	-2.62440200	-3.20850900
C	3.84790700	-0.81348000	-2.03148200
H	4.94579000	-0.82615000	-2.08537500
C	3.20321200	0.21581900	-1.32891900
H	3.78344500	1.01007800	-0.84236700
C	1.80497800	0.23617300	-1.24473900
C	1.32435000	2.61480900	-0.80639000
C	2.39664300	3.34947400	-0.29151600
H	3.10744800	2.86120900	0.39146800
C	2.53181500	4.71110200	-0.63514400
C	1.55466800	5.29665400	-1.46569400
H	1.64930800	6.36364900	-1.72575500
C	0.43872600	4.59152000	-1.96250400
C	0.33907800	3.23267500	-1.61867700
C	-0.62733100	5.23286700	-2.80700800
C	3.70578500	5.51216200	-0.12767400
H	-1.62063900	5.15152100	-2.31767000
H	-0.41052900	6.30286500	-2.98721900
H	-0.72423700	4.72421800	-3.78855900
H	3.52512800	6.60111900	-0.21729500
H	3.92210700	5.28779900	0.93659200

H	4.63047600	5.28259700	-0.70017700
H	5.39554500	-3.75268100	-1.31001100
H	6.24851700	-4.30787000	0.15798200
H	4.63895000	-4.98139500	-0.25873100
H	5.33114000	0.52889600	3.35624000
H	6.67494300	0.13877700	2.22235200
H	5.36563500	1.27518900	1.74892600
H	-3.79240800	-5.24385300	-3.03757400
H	-2.27828600	-5.02694500	-3.96844800
H	-2.20214100	-5.48122100	-2.24512500
H	-6.06097800	-1.23268800	-1.64289600
H	-5.10786400	-0.20456500	-0.51150900
H	-5.29965800	0.29623200	-2.20267000
H	-5.24378100	-0.92262700	4.30853600
H	-5.14801200	0.65013600	5.15360800
H	-6.32670400	0.42604300	3.83568200
H	-4.43252700	4.32635300	1.37845300
H	-2.63974400	4.46220300	1.42143900
H	-3.42452800	3.75515600	-0.00345300

10^{Me}

As	-1.08651200	0.59232300	-0.83647100
As	0.96813700	0.78933000	1.67369600
O	-1.77430100	2.15988200	-0.05280300
O	2.84186400	0.92883300	1.82028900
O	-2.74973900	-0.00686100	-1.36385000
O	-0.76139200	1.66957600	-2.35057400
N	-1.15233400	-0.06403300	1.10118500
N	1.29594400	-1.08359600	1.59913100
N	-0.40840900	-1.07189000	-1.47666600
N	0.91262200	1.01670500	-0.47117500
C	-2.40742500	1.92922300	1.12507600
C	-2.11949100	0.71581900	1.78851500
C	-2.72546400	0.41371800	3.01623700
H	-2.48609200	-0.53146500	3.52548600
C	-3.63661400	1.32561900	3.58627600
C	-3.92552100	2.51929200	2.89260200
H	-4.64404200	3.23101300	3.33065200
C	-3.33722600	2.84392900	1.65506700
C	-3.64919700	4.10363200	0.89621700
C	-4.26426100	1.03705700	4.92847500
C	-1.07517300	-1.46378200	1.35258300
C	0.21900200	-1.98335600	1.63490600
C	0.32892300	-3.34912600	1.97805000
H	1.29825000	-3.75822600	2.28416500
C	-0.80367900	-4.17567300	1.96794000
H	-0.68995700	-5.23791600	2.23196500
C	-2.06918400	-3.66340900	1.64162900
H	-2.95191400	-4.31808600	1.61945900
C	-2.20163100	-2.29995000	1.35134500
H	-3.18166100	-1.86385900	1.11314900
C	2.66255700	-1.39242200	1.45173700
C	3.24612800	-2.61204000	1.06708300
H	2.62218700	-3.48551000	0.84410000
C	4.64170100	-2.70060900	0.88832600

C	5.43930900	-1.56134500	1.10710700
H	6.53149500	-1.63629800	0.98181500
C	4.88424100	-0.31184400	1.44482100
C	3.48951100	-0.24790300	1.59273800
C	5.70834500	0.93745200	1.59259800
C	5.24989400	-3.99561800	0.40879600
C	-2.71587500	-1.33372000	-1.70796700
C	-1.43919900	-1.95928600	-1.78305200
C	-1.36309400	-3.34158100	-2.01598300
H	-0.39447800	-3.85638900	-1.98966500
C	-2.54254800	-4.09060200	-2.21619200
C	-3.78609000	-3.43485300	-2.17333800
H	-4.70805400	-4.01742500	-2.33046900
C	-3.90209600	-2.05293100	-1.91001800
C	-5.22583400	-1.34582000	-1.80724700
C	-2.45094600	-5.57844400	-2.45388200
C	0.95394800	-1.10238200	-1.69609300
C	1.66726000	-2.09270700	-2.41000200
H	1.13155400	-2.94500500	-2.84240600
C	3.05002800	-1.96571000	-2.59429000
H	3.58832500	-2.75414700	-3.14211800
C	3.74867200	-0.84593000	-2.11362900
H	4.83283500	-0.75287200	-2.26554900
C	3.05037600	0.15929200	-1.43220200
H	3.56854200	1.05335000	-1.06144100
C	1.67780800	0.02171100	-1.19893100
C	1.07241500	2.35209100	-0.99931200
C	1.99868900	3.30240200	-0.55933900
H	2.69133100	3.05483900	0.25924200
C	2.03361900	4.56406600	-1.18663800
C	1.11425900	4.82338600	-2.22662900
H	1.13163400	5.81243700	-2.71366600
C	0.15350500	3.89171400	-2.66157800
C	0.14620200	2.62856100	-2.03265900
C	-0.86281100	4.19108300	-3.72703700
C	3.04697800	5.60394100	-0.77574200
H	-1.88844000	4.00435300	-3.34641300
H	-0.79350700	5.24044700	-4.07097700
H	-0.73427200	3.52437400	-4.60512900
H	2.60592300	6.62112600	-0.76598700
H	3.45522300	5.39879500	0.23321700
H	3.90670300	5.63233800	-1.47986200
H	4.99802100	-4.17782200	-0.65888600
H	6.35391700	-3.98447000	0.49264800
H	4.87244000	-4.86738600	0.98142100
H	5.51296200	1.44094400	2.56147600
H	6.79027700	0.71636600	1.52169000
H	5.45684600	1.67921300	0.80352200
H	-3.45403500	-6.03726300	-2.54991600
H	-1.88456000	-5.81122400	-3.38033400
H	-1.92431600	-6.08814200	-1.61938300
H	-6.06938000	-2.04536000	-1.96039100
H	-5.34448400	-0.86090400	-0.81518500
H	-5.30562100	-0.53054600	-2.55615600
H	-4.50277100	-0.03922000	5.04588500
H	-3.58112200	1.30939500	5.76220200

H	-5.20003500	1.61222600	5.07172700
H	-4.42644700	4.70221800	1.40790500
H	-2.74330100	4.73369700	0.77406600
H	-3.99986300	3.86843500	-0.13031100

NMR Spectra

Compound **2**

^{1}H NMR (400 MHz, CD_2Cl_2)

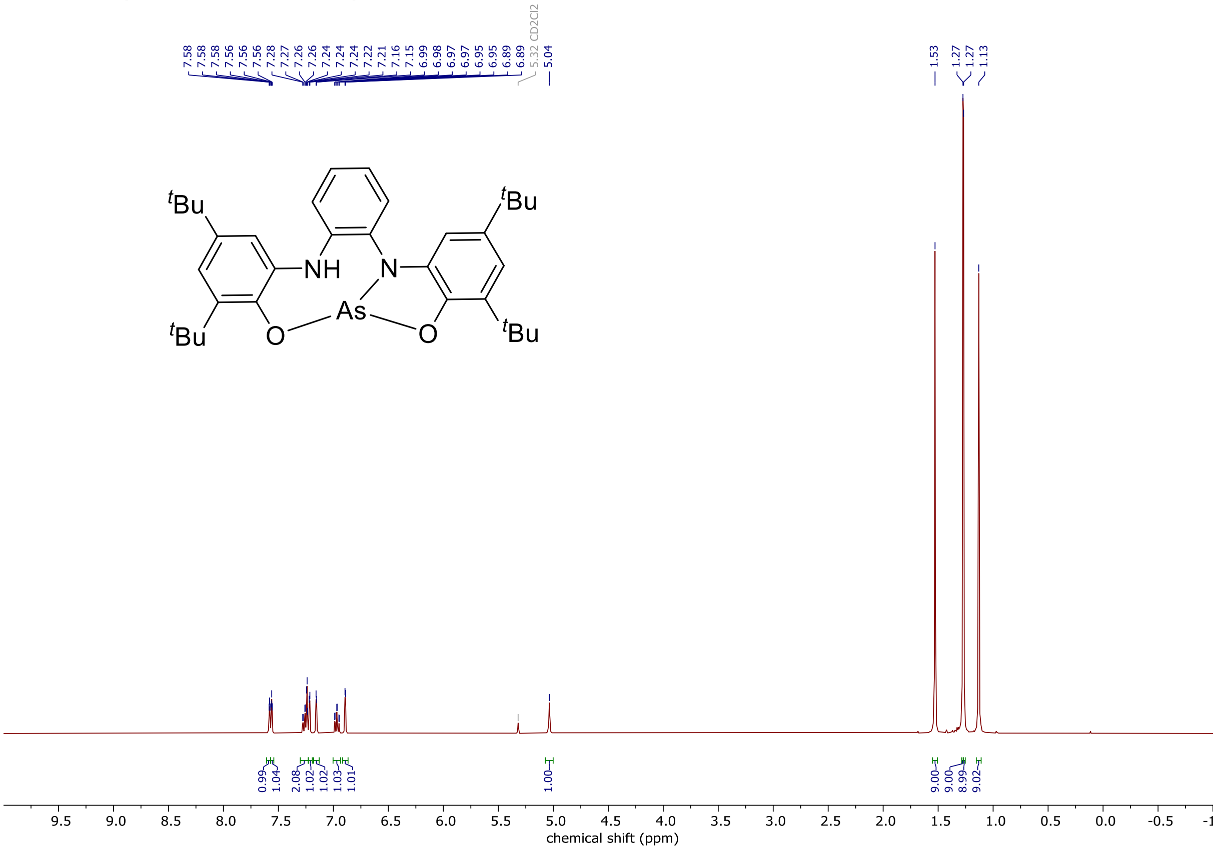

^{13}C{^{1}H} NMR (101 MHz, CD_2Cl_2)

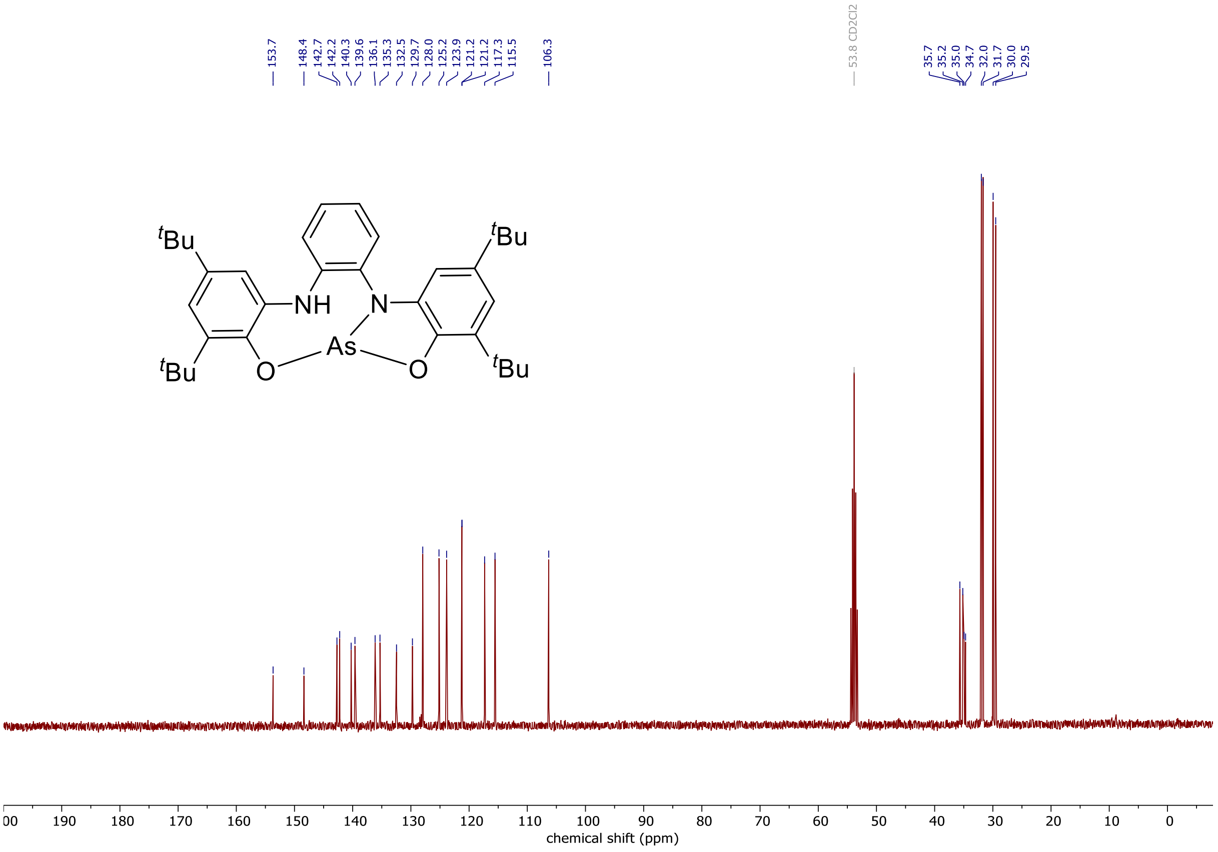

Compound **3**

^{1}**H NMR** (300 MHz, CD_3CN)

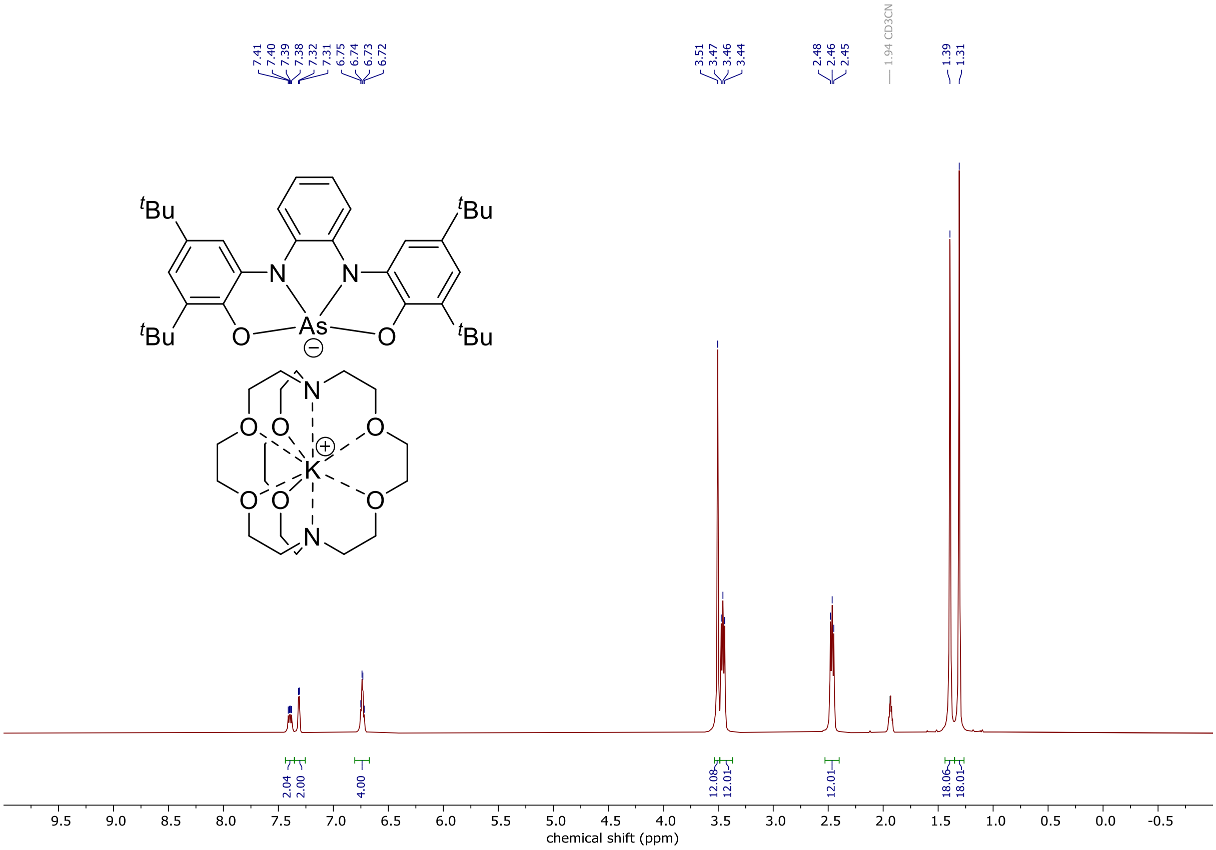

^{13}**C{^{1}H} NMR** (101 MHz, CD_3CN)

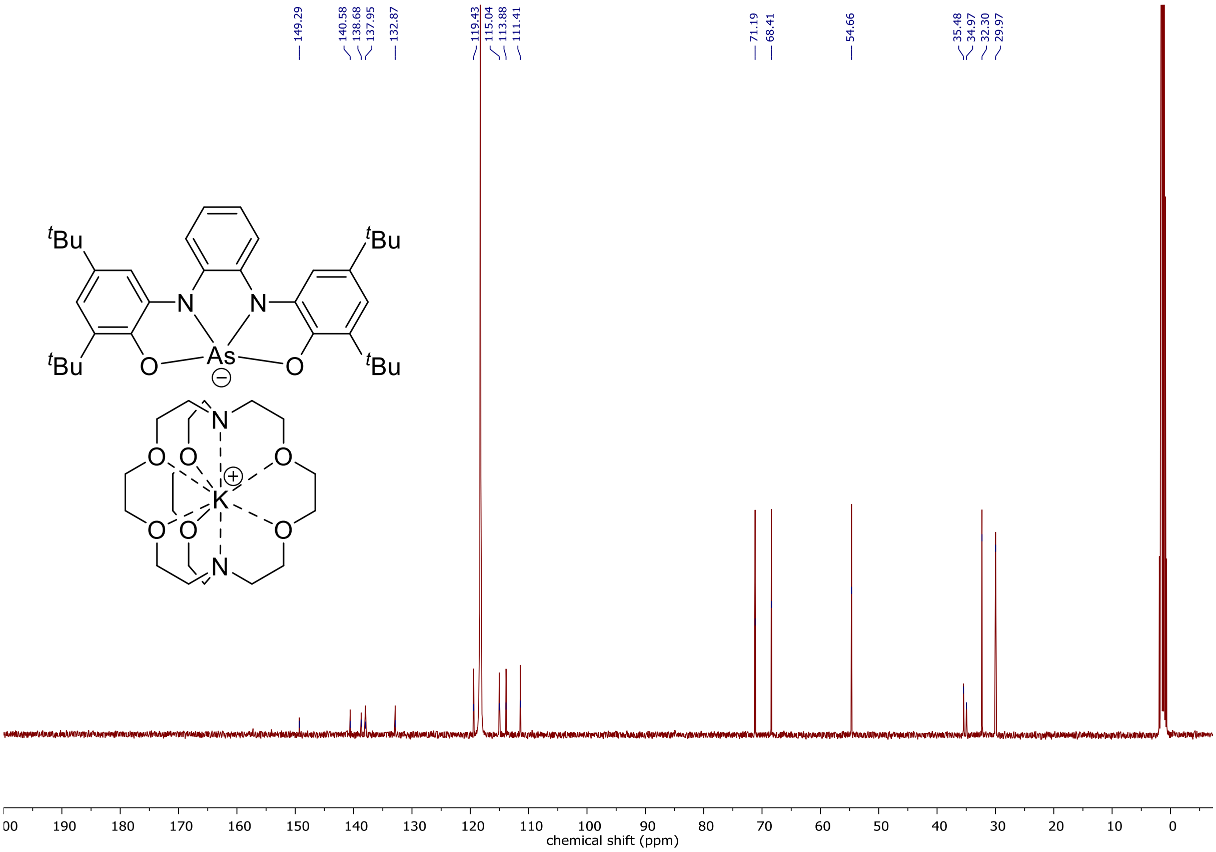

Compound **4**

^{1}H NMR (300 MHz, $CDCl_3$)

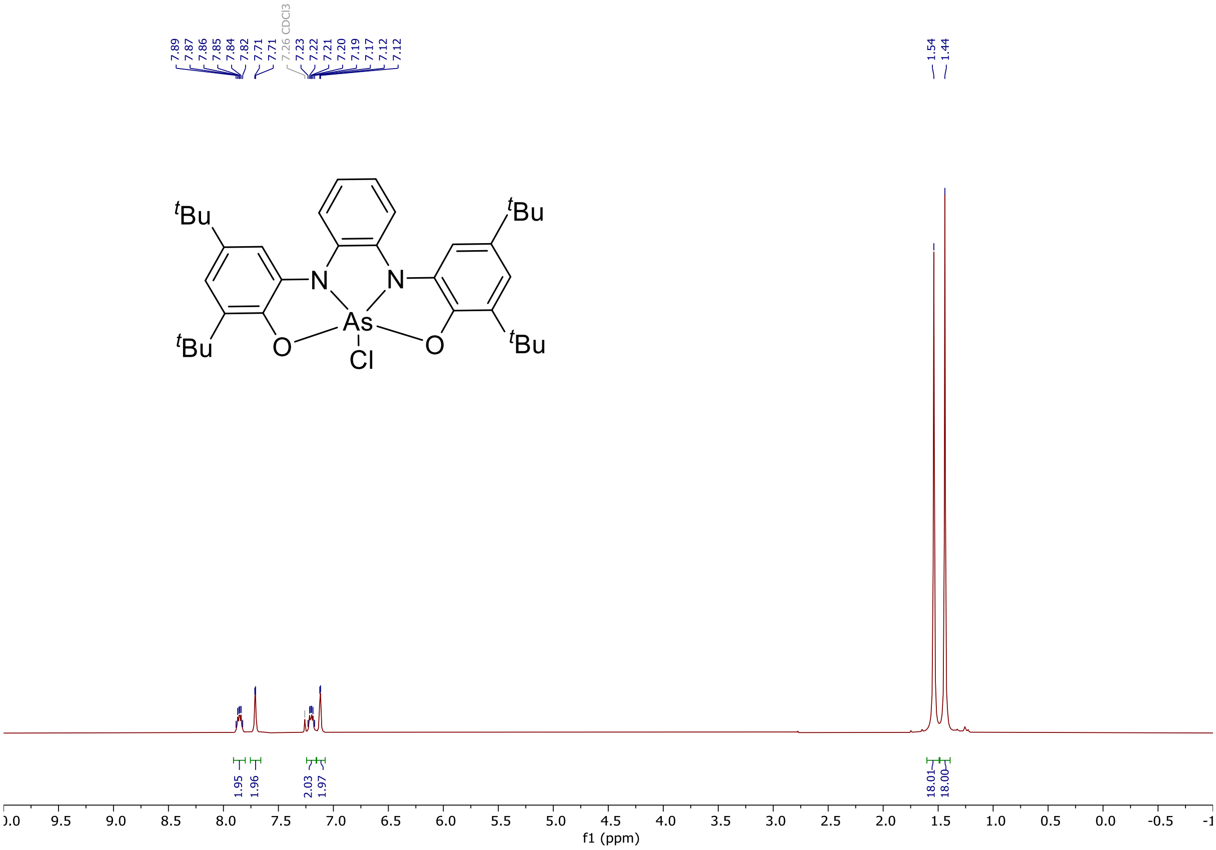

^{13}C{^{1}H} NMR (75 MHz, $CDCl_3$)

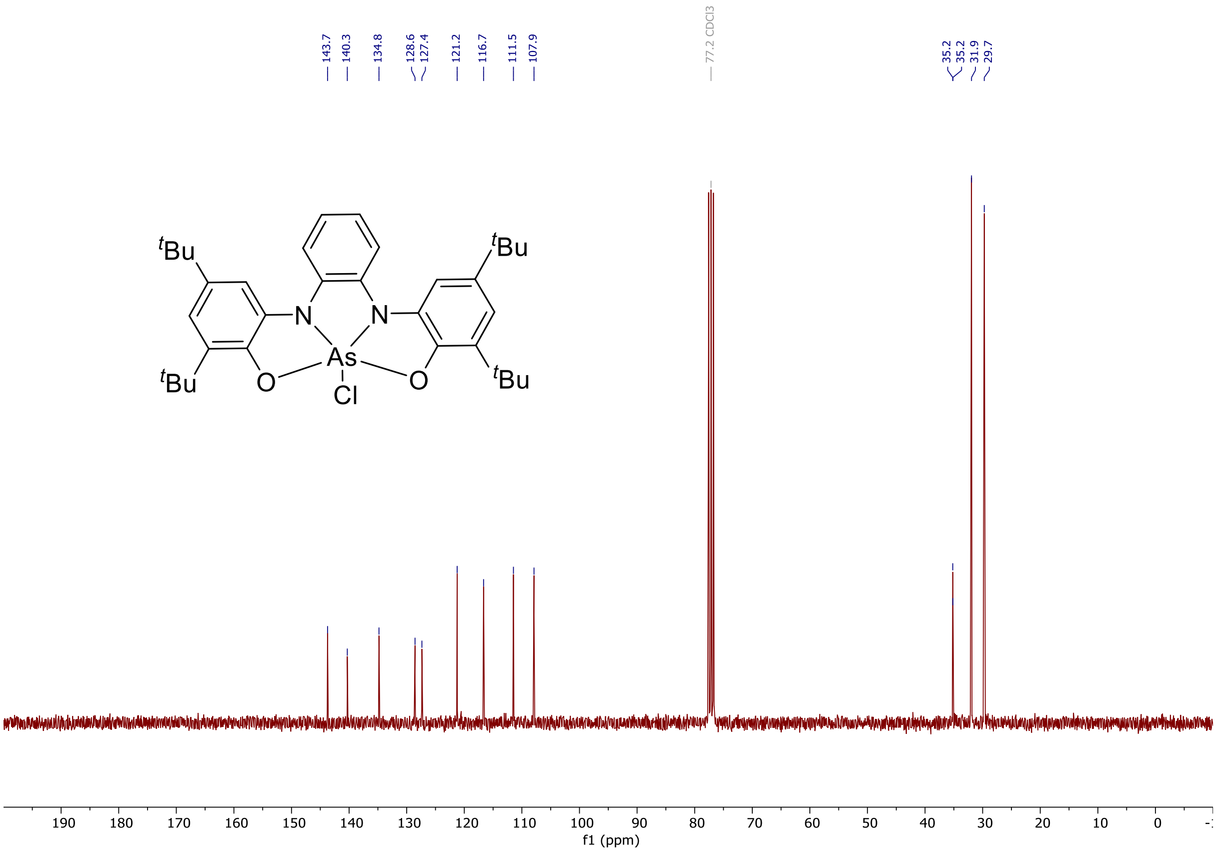

Compound **5**

^{1}H NMR (400 MHz, $CDCl_3$)

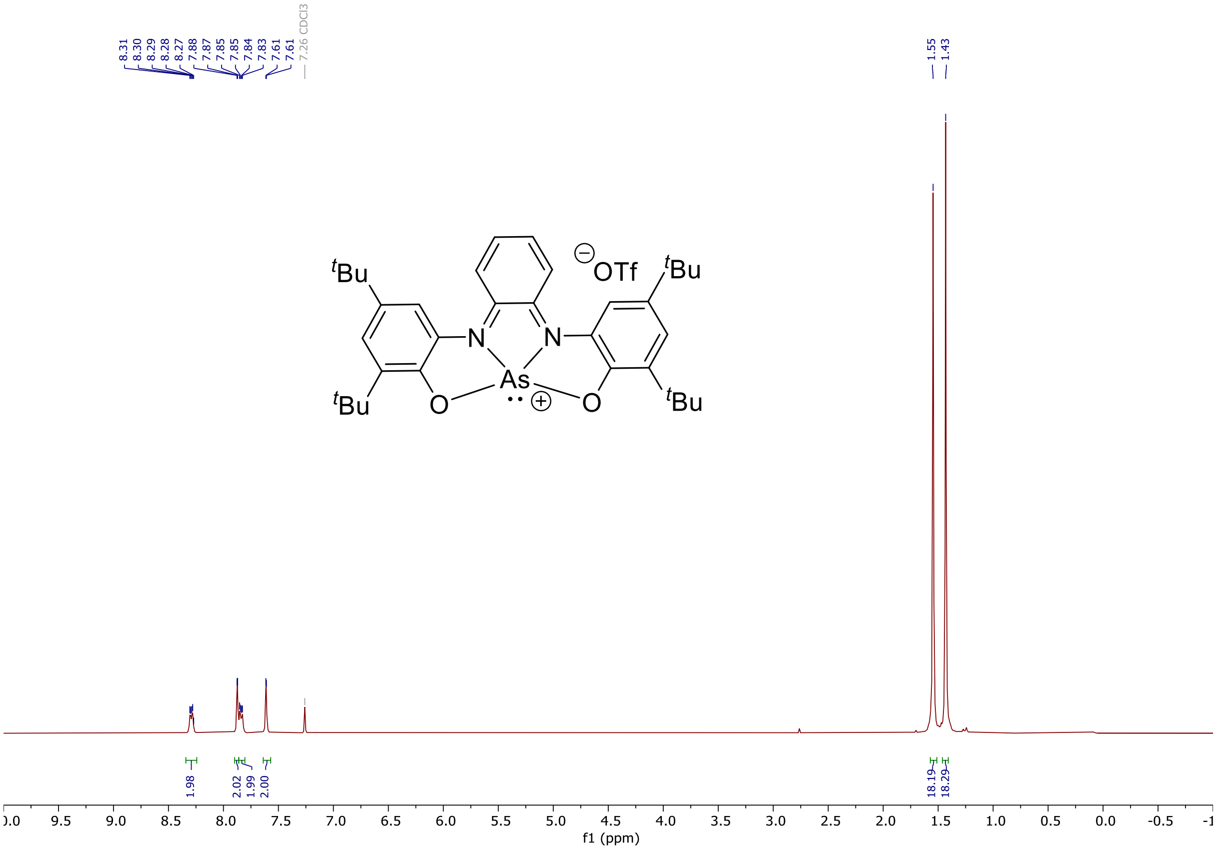

^{13}C{^{1}H} NMR (101 MHz, $CDCl_3$)

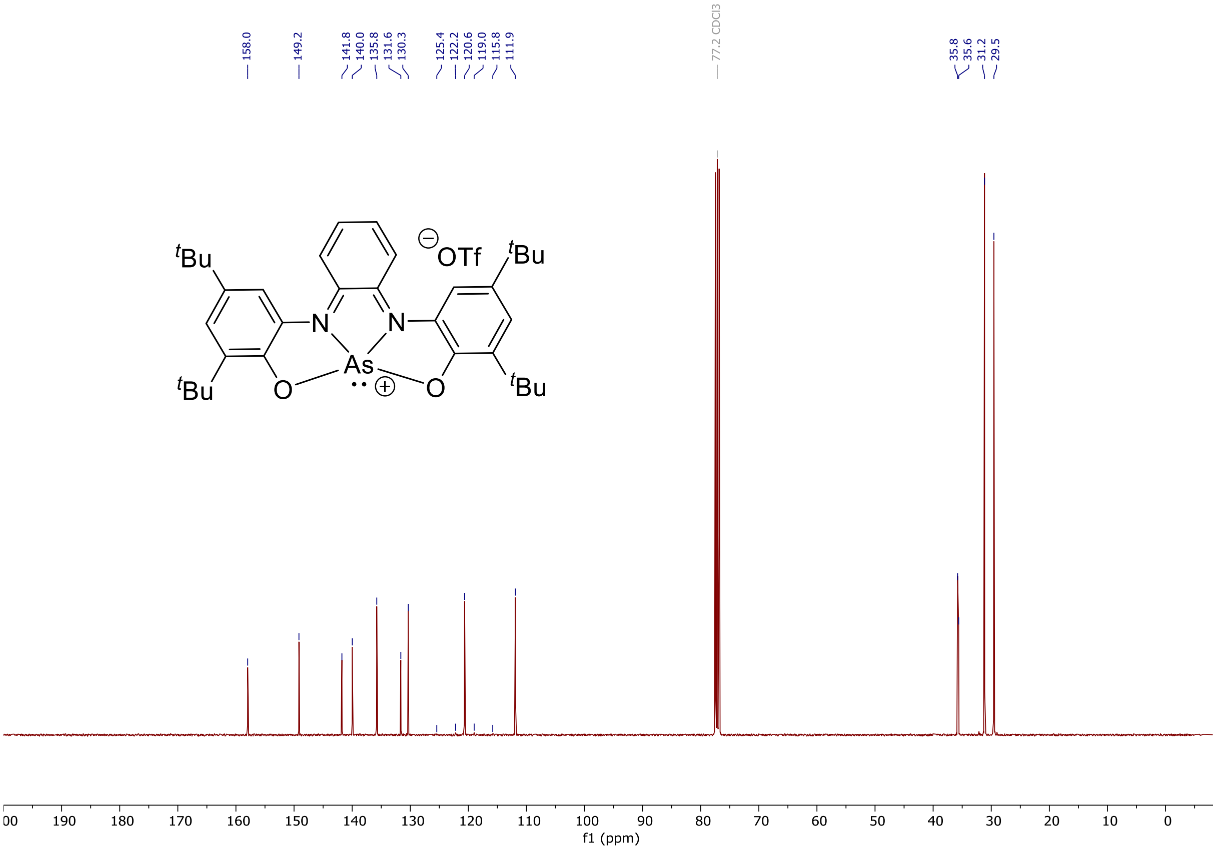

^{19}F NMR (377 MHz, $CDCl_3$)

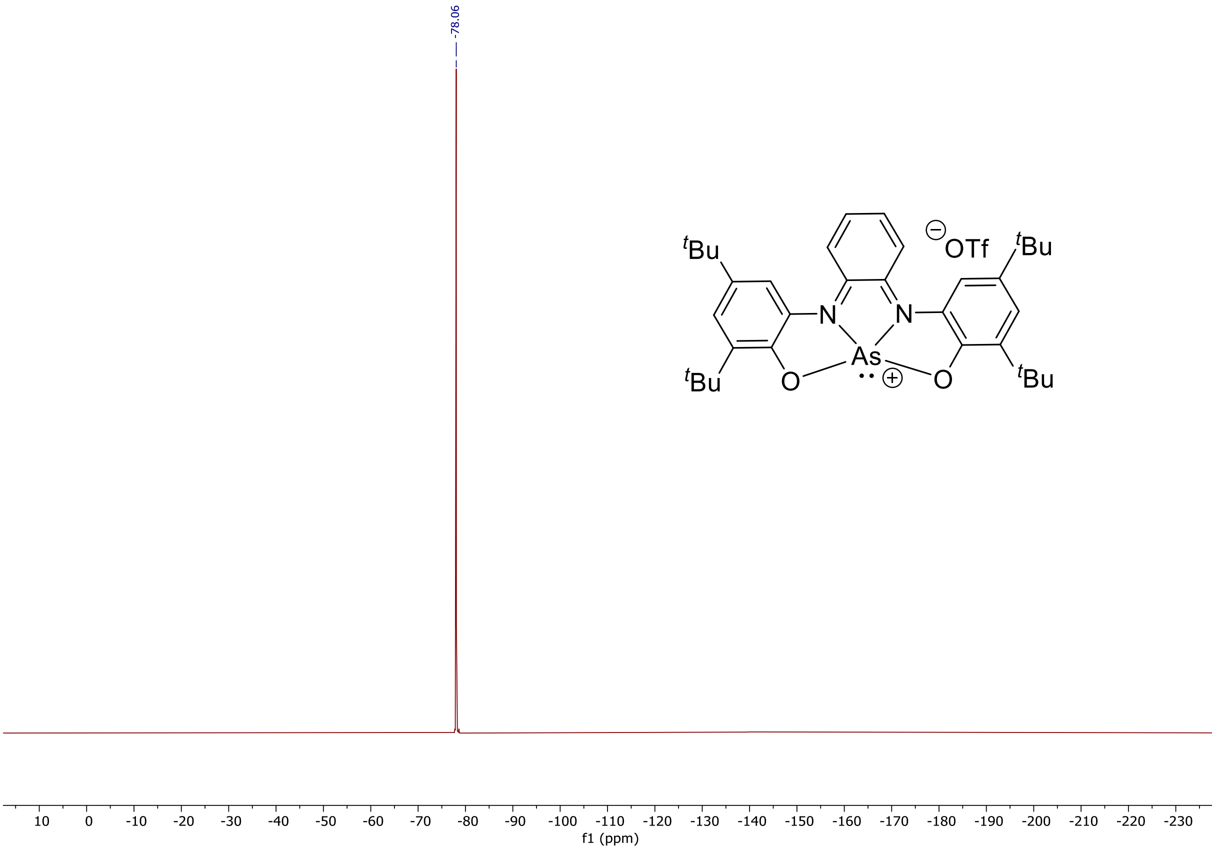

Compound **6**

^{1}H NMR (400 MHz, $CDCl_3$)

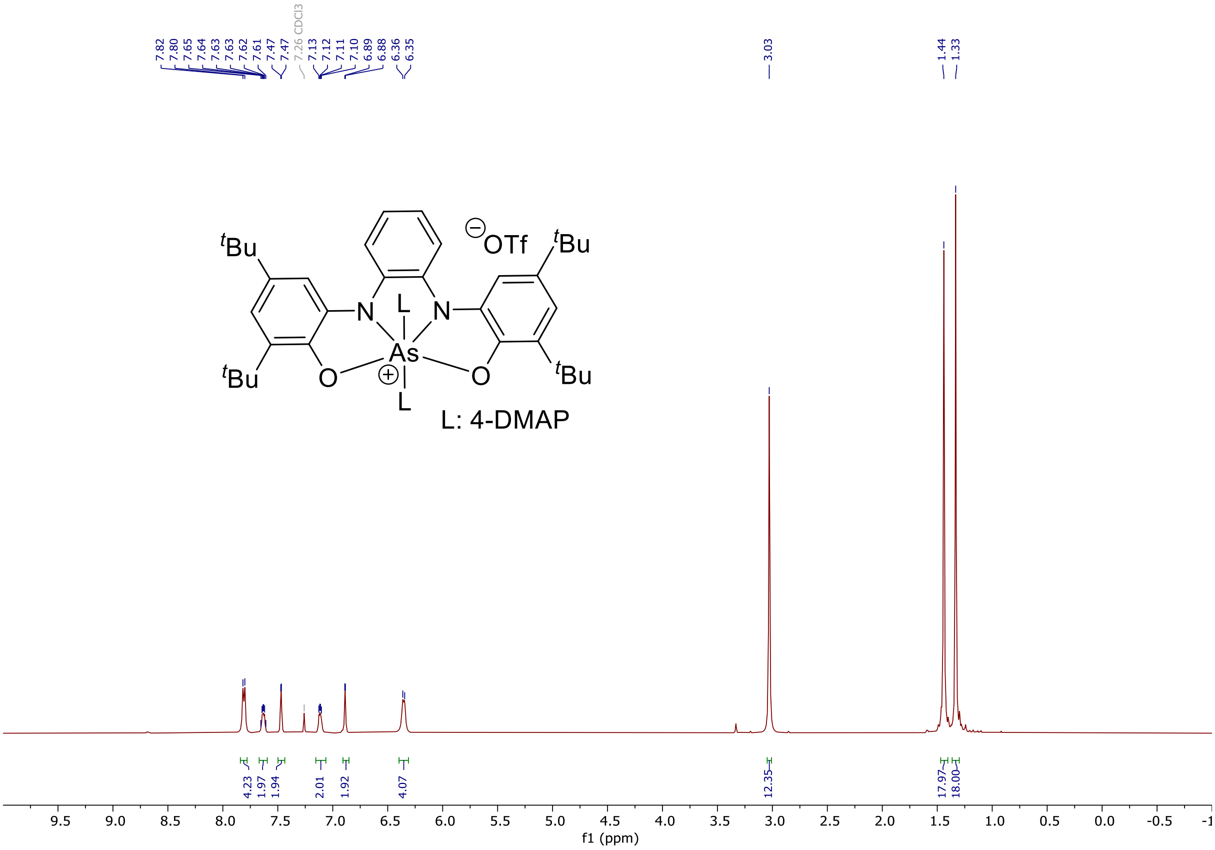

$^{13}C\{^1H\}$ NMR (101 MHz, $CDCl_3$)

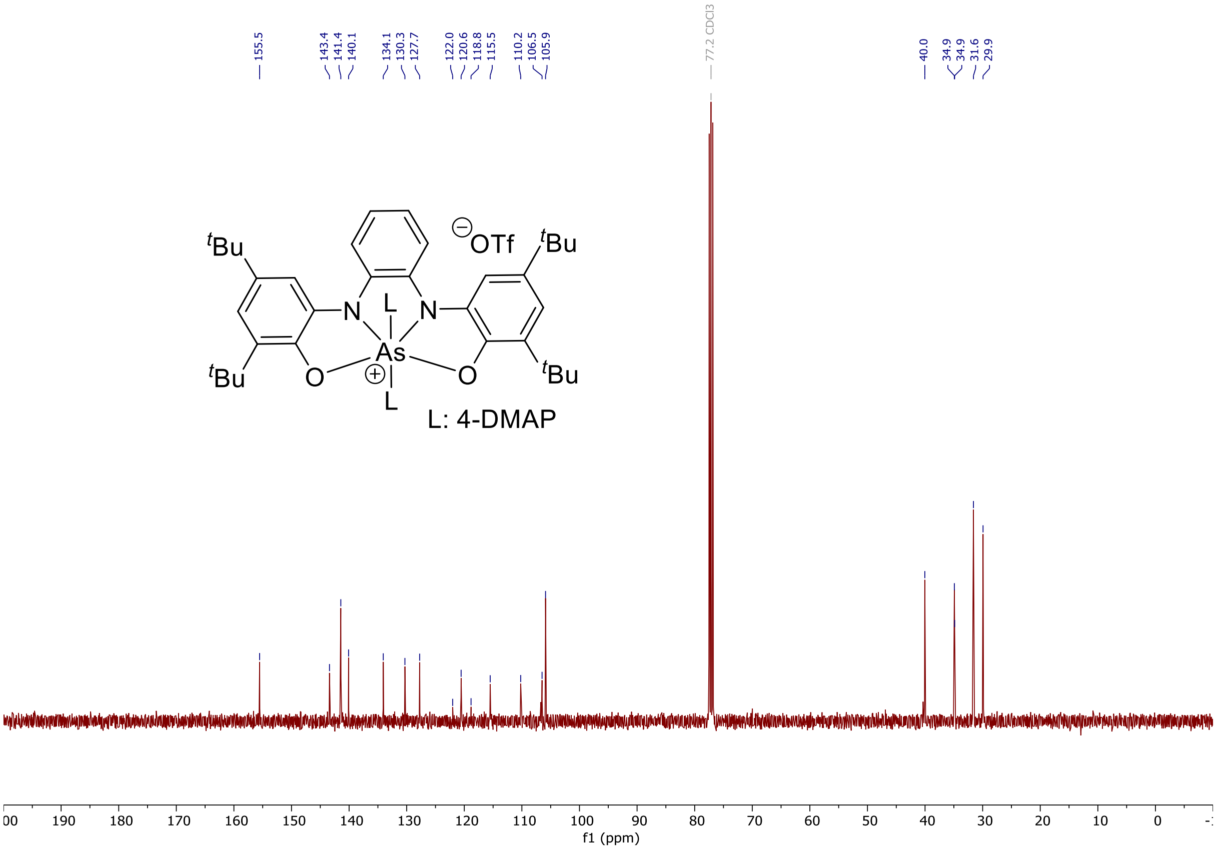

^{19}F NMR (377 MHz, $CDCl_3$)

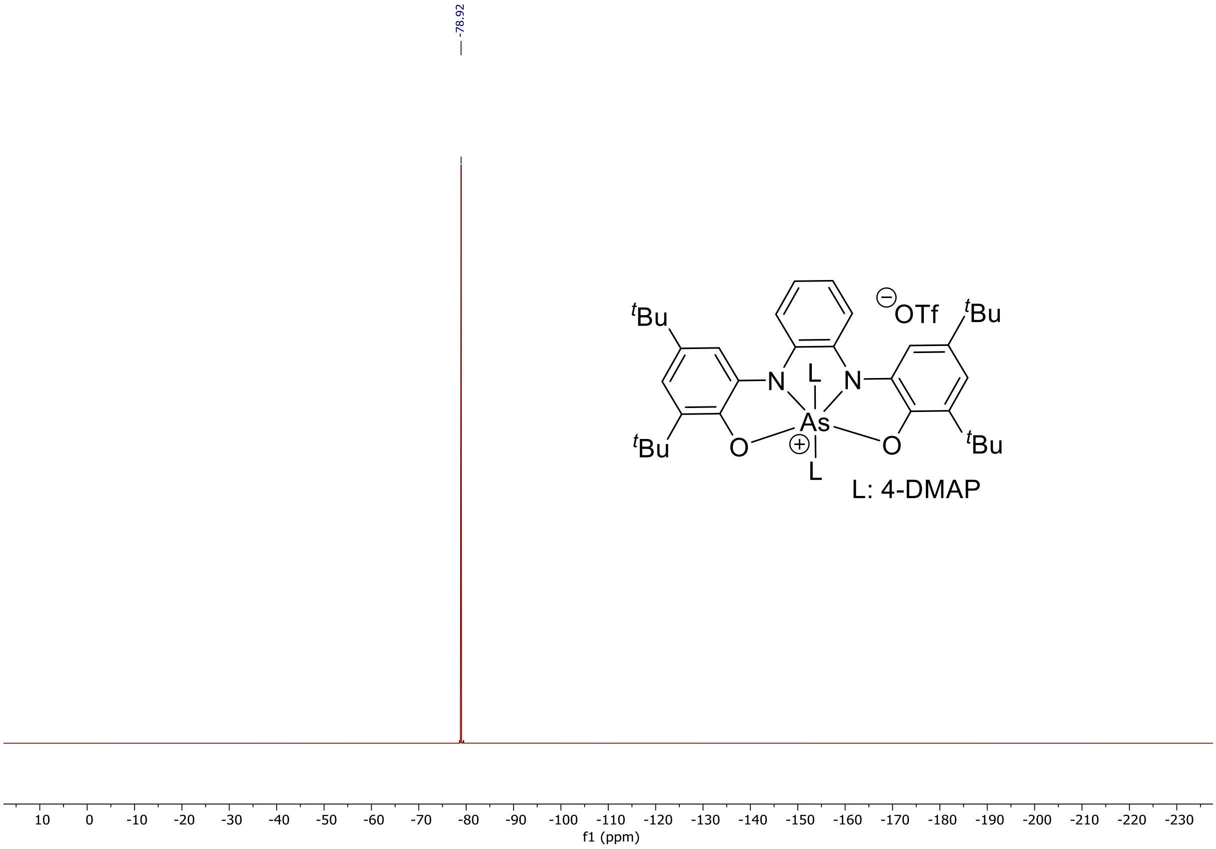

Compound **7**

^{1}**H NMR** (300 MHz, CD_3CN)

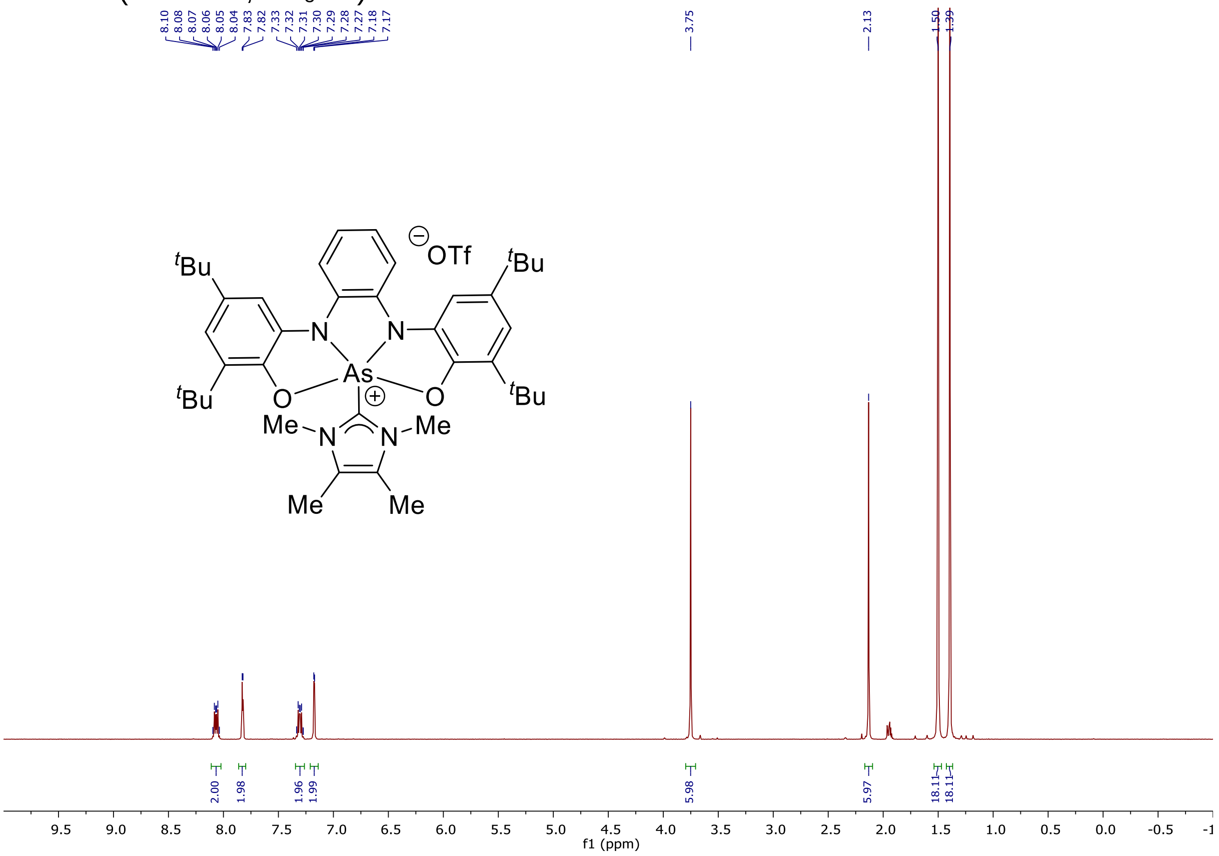

^{13}**C{^{1}H} NMR** (126 MHz, CD_3CN)

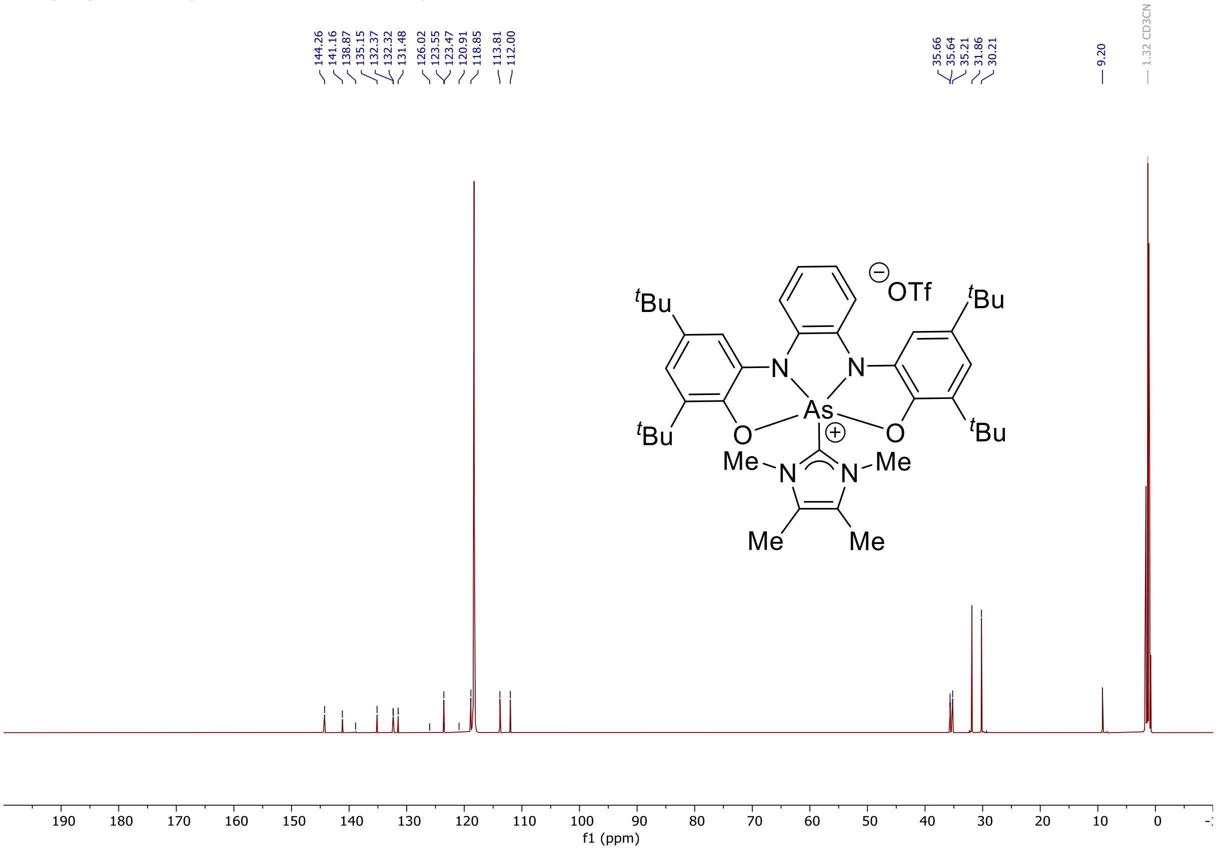

$^{19}F\{^{1}H\}$ NMR (282 MHz, CD_3CN)

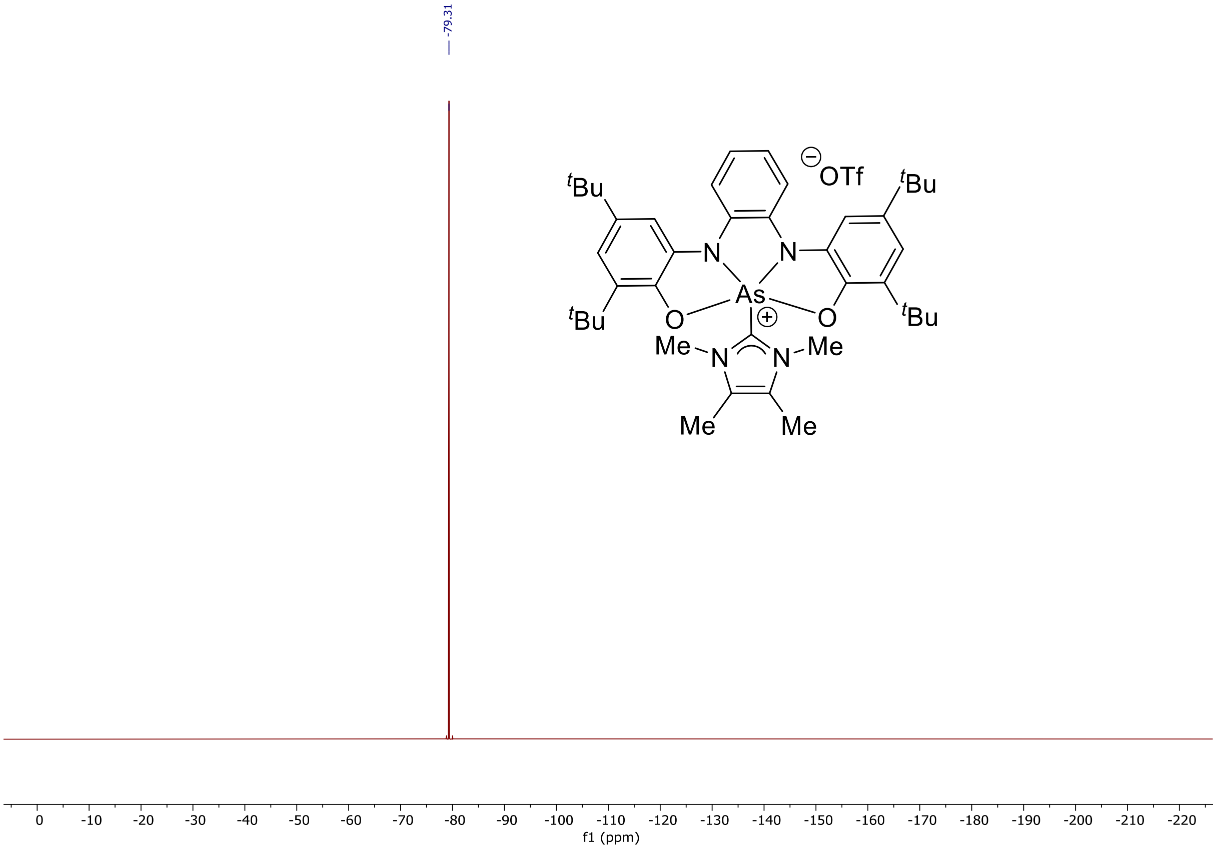

Compound **8**

^{1}H NMR (400 MHz, $CDCl_3$)

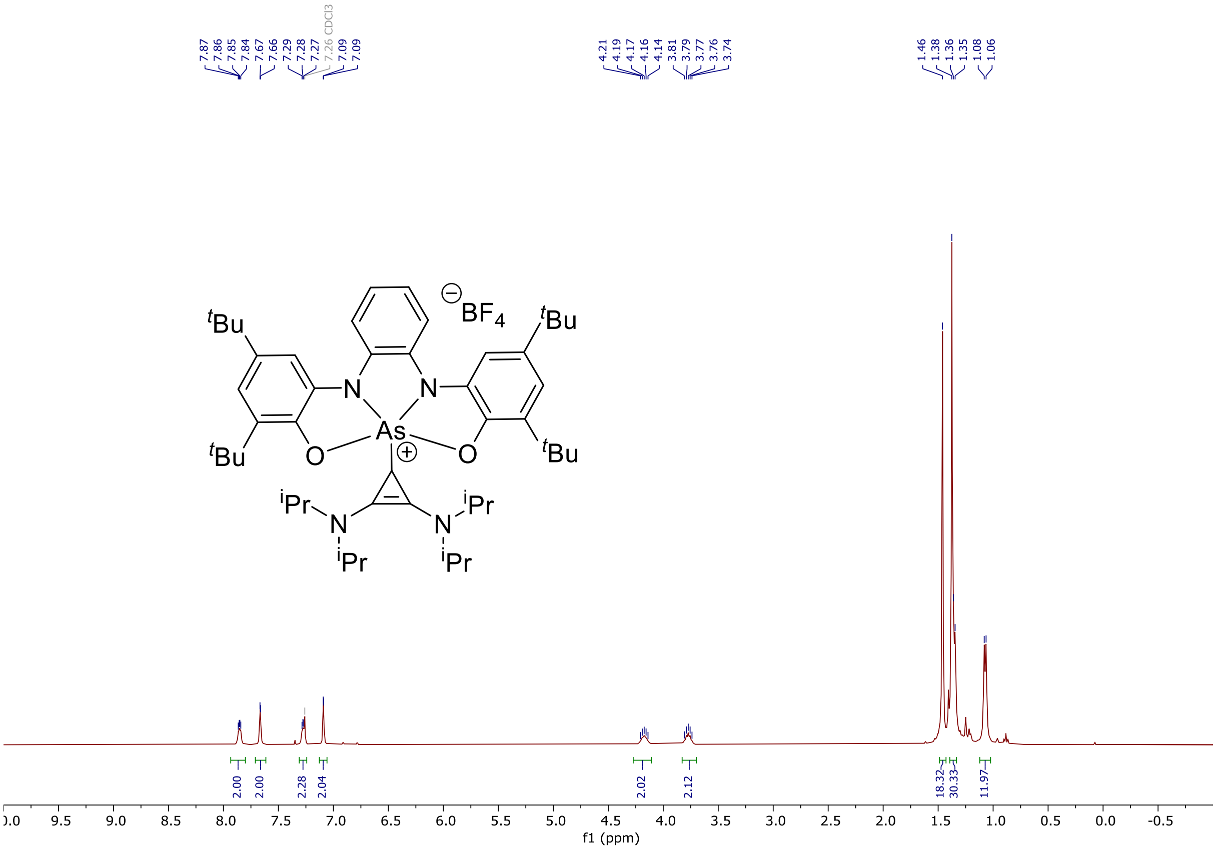

$^{11}B\{^{1}H\}$ NMR (96 MHz, $CDCl_3$)

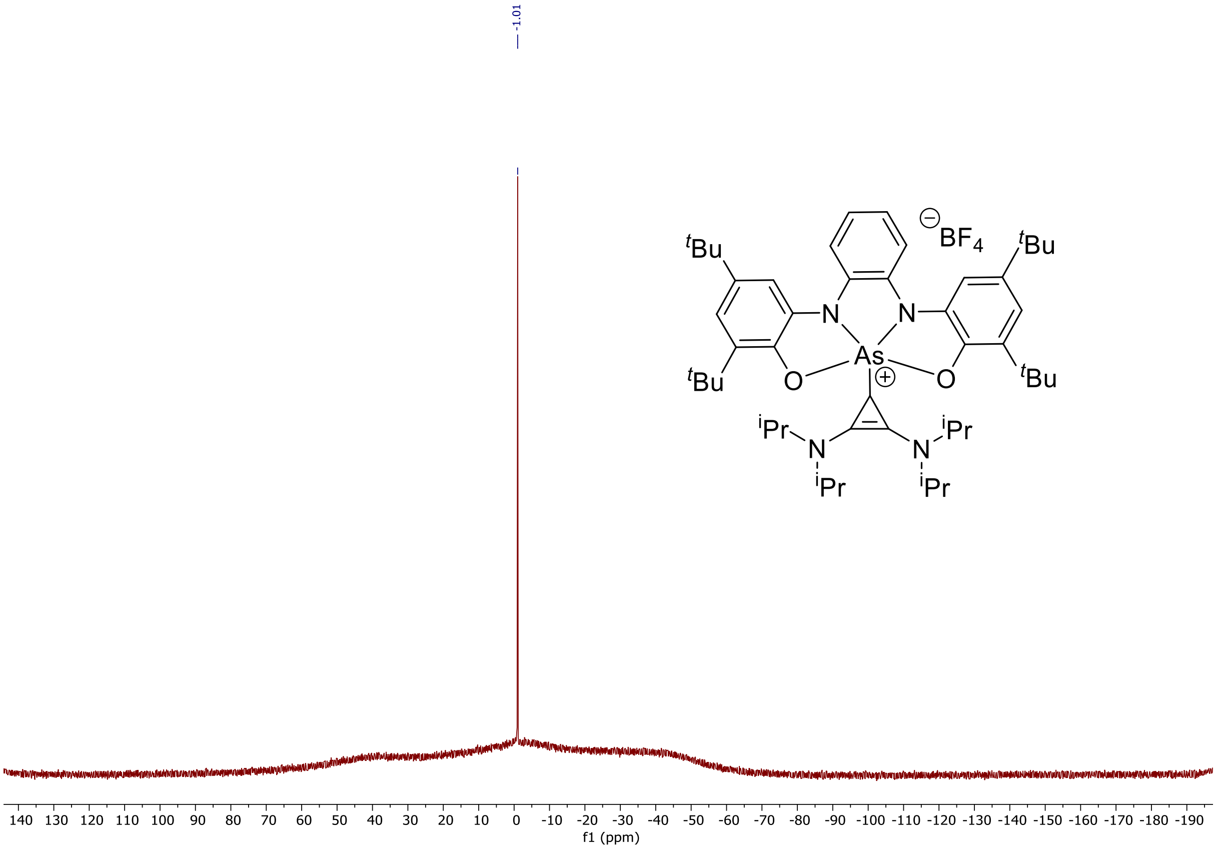

$^{13}C\{^{1}H\}$ NMR (101 MHz, $CDCl_3$)

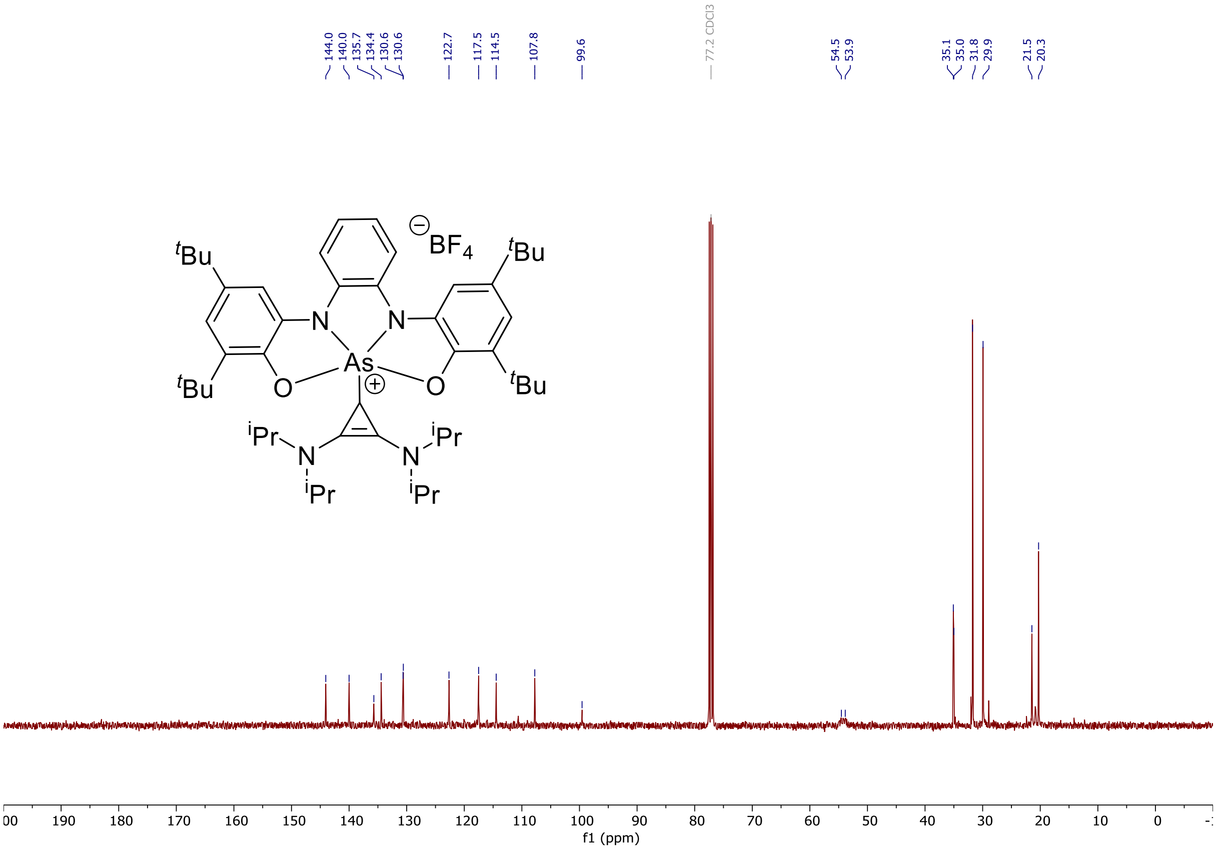

$^{19}F\{^1H\}$ NMR (282 MHz, $CDCl_3$)

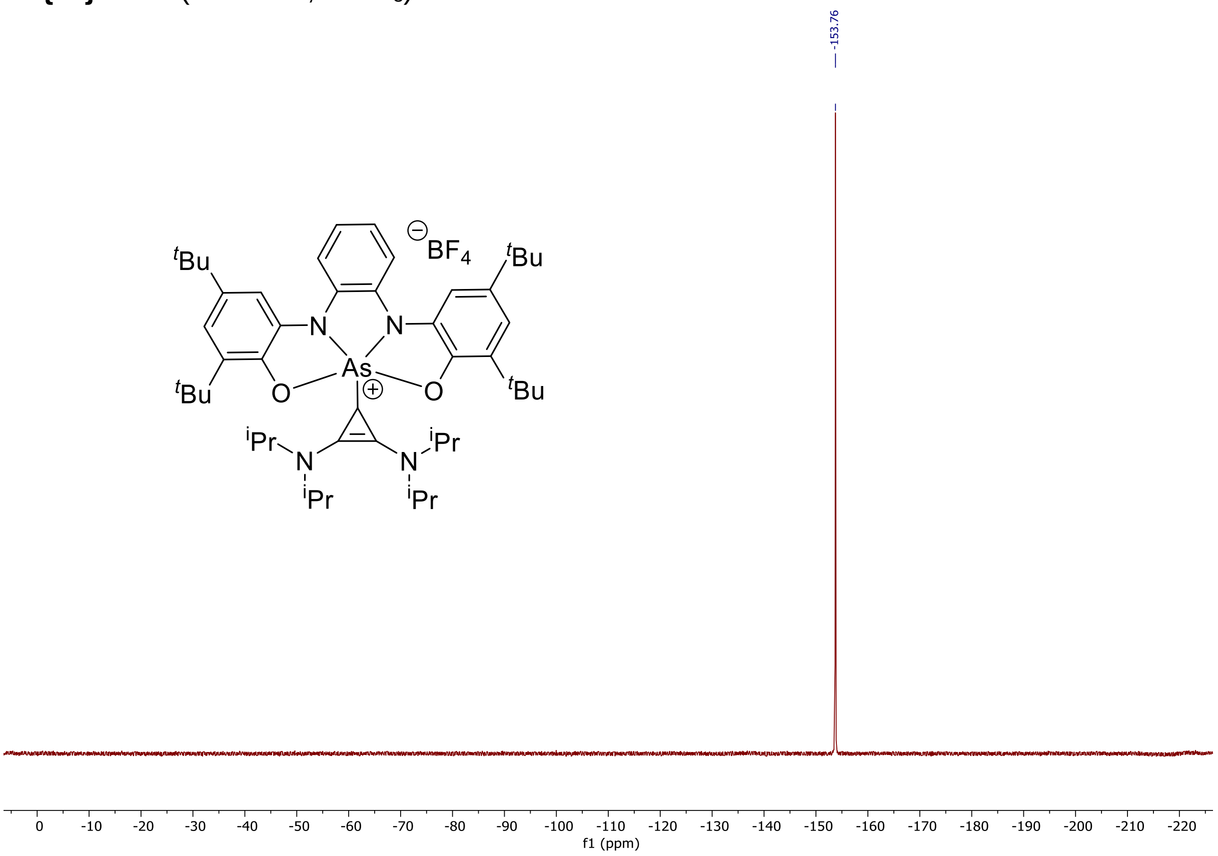

Compound **10**

^1H NMR (400 MHz, CD_2Cl_2, 233 K)

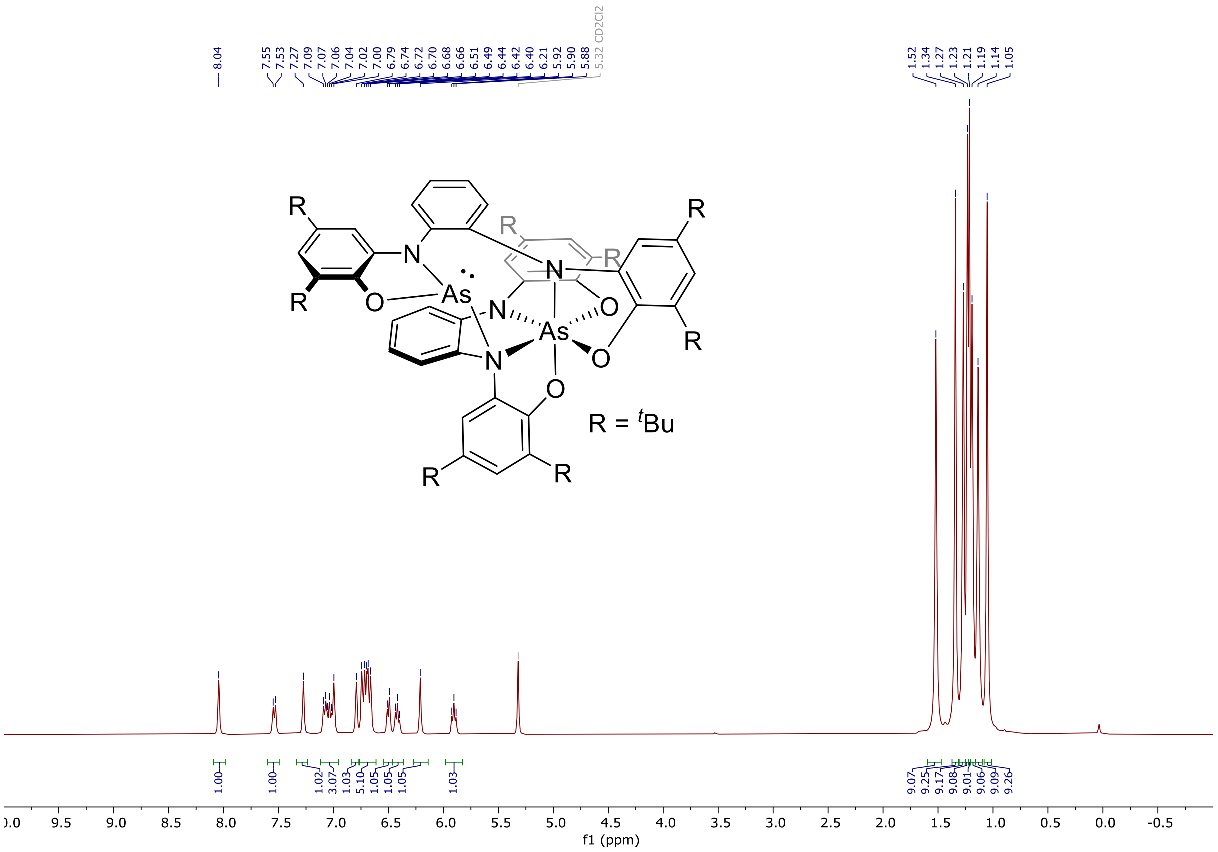

$^{13}C\{^{1}H\}$ NMR (101 MHz, CD_2Cl_2, 233 K)

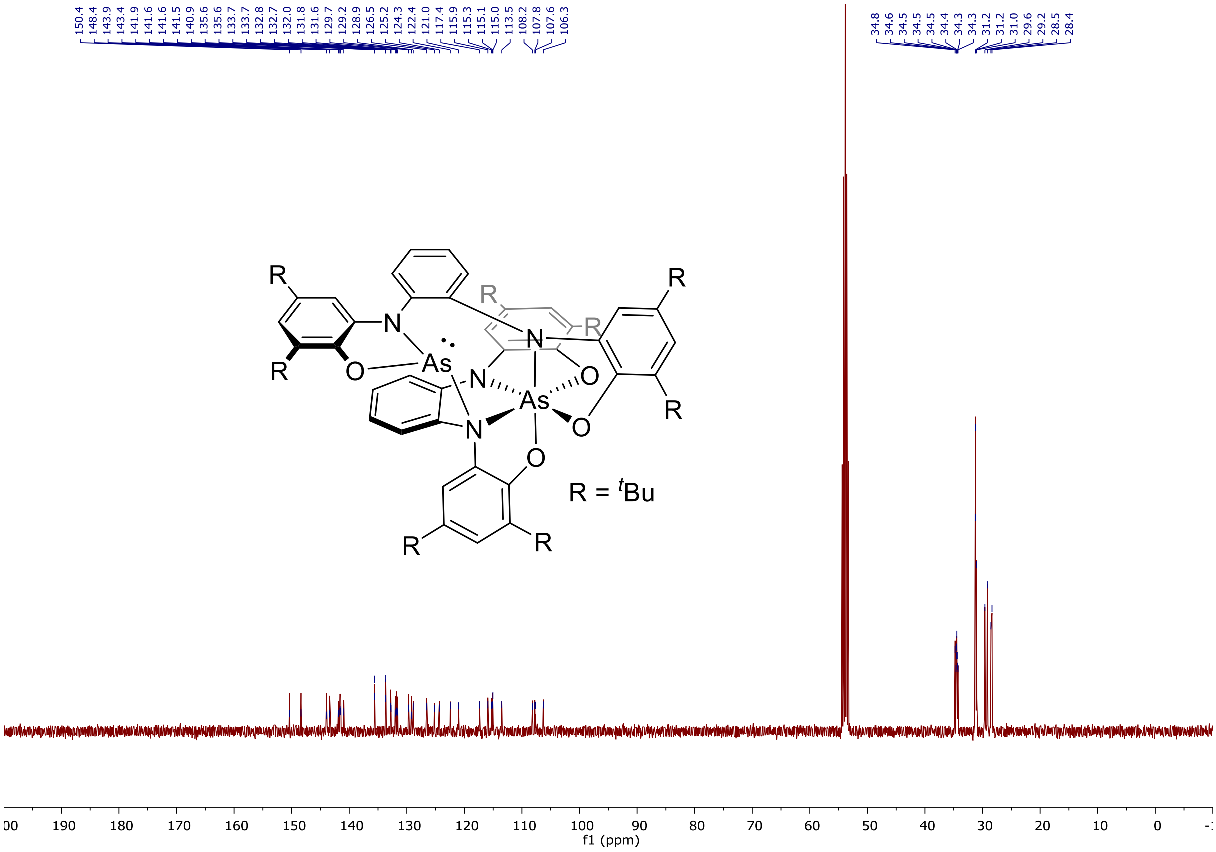

References

[1] P. Chaudhuri, M. Hess, J. Müller, K. Hildenbrand, E. Bill, T. Weyhermüller, K. Wieghardt, *J. Am. Chem. Soc.* **1999**, *121*, 9599.
[2] N. Kuhn, T. Kratz, *Synthesis* **1993**, *1993*, 561.
[3] V. Lavallo, Y. Ishida, B. Donnadieu, G. Bertrand, *Angew. Chem. Int. Ed.* **2006**, *45*, 6652.
[4] a) A. Jerschow, N. Müller, *J. Magn. Reson.* **1996**, *123*, 222; b) A. Jerschow, N. Müller, *J. Magn. Reson.* **1997**, *125*, 372.
[5] a) S. Bachmann, B. Gernert, D. Stalke, *Chem. Commun.* **2016**, *52*, 12861; b) S. Bachmann, R. Neufeld, M. Dzemski, D. Stalke, *Chem. Eur. J.* **2016**, *22*, 8462; c) R. Neufeld, D. Stalke, *Chem. Sci.* **2015**, *6*, 3354.
[6] I. Chatterjee, Z.-W. Qu, S. Grimme, M. Oestreich, *Angew. Chem. Int. Ed.* **2015**, *54*, 12158.
[7] J. C. L. Walker, M. Oestreich, *Org. Lett.* **2018**, *20*, 6411.
[8] G. M. Sheldrick, *Acta Cryst. A* **2008**, *64*, 112.
[9] O. V. Dolomanov, L. J. Bourhis, R. J. Gildea, J. A. K. Howard, H. Puschmann, *J. Appl. Crystallogr.* **2009**, *42*, 339.
[10] D. Kratzert, *FinalCif*, Kratzert, D.
[11] S. N. Brown, *Inorg. Chem.* **2012**, *51*, 1251.
[12] M. J. Frisch, G. W. Trucks, H. B. Schlegel, G. E. Scuseria, M. A. Robb, J. R. Cheeseman, G. Scalmani, V. Barone, G. A. Petersson, H. Nakatsuji et al., *Gaussian 16 Rev. A.03*, Wallingford, CT, **2016**.
[13] a) A. D. Becke, *Phys. Rev. A* **1988**, *38*, 3098; b) A. D. Becke, *J. Chem. Phys.* **1993**, *98*, 5648.
[14] a) A. Schäfer, H. Horn, R. Ahlrichs, *J. Chem. Phys.* **1992**, *97*, 2571; b) F. Weigend, R. Ahlrichs, *Phys. Chem. Chem. Phys.* **2005**, *7*, 3297; c) F. Weigend, *Phys. Chem. Chem. Phys.* **2006**, *8*, 1057.
[15] a) S. Grimme, J. Antony, S. Ehrlich, H. Krieg, *J. Chem. Phys.* **2010**, *132*, 154104; b) S. Grimme, S. Ehrlich, L. Goerigk, *J. Comput. Chem.* **2011**, *32*, 1456.
[16] G. Knizia, J. E. M. N. Klein, *Angew. Chem. Int. Ed.* **2015**, *54*, 5518.
[17] P. Erdmann, J. Leitner, J. Schwarz, L. Greb, *ChemPhysChem* **2020**, *21*, 987.
[18] Y. Zhao, D. G. Truhlar, *J. Phys. Chem.* **2005**, *109*, 5656.
[19] a) J. P. Perdew, K. Burke, M. Ernzerhof, *Phys. Rev. Lett.* **1997**, *78*, 1396; b) Perdew, Burke, Ernzerhof, *Phys. Rev. Lett.* **1996**, *77*, 3865.
